# Influence of Donor/Withdrawing Groups in an 3,5-Aryl-Substituted Pyrazole Organocatalyst for the Chemical Fixation of CO_2_

**DOI:** 10.1021/acsomega.4c11307

**Published:** 2025-06-06

**Authors:** Gabriel Elias Taliateli Oliveira Prado, Karine Braga Enes, Álvaro Farias Arruda da Mata, Gabriel Cerqueira, Marcone Augusto Leal de Oliveira, Luiz Antônio Sodré Costa, Felipe Terra Martins, Meiry Edivirges Alvarenga, Rafael Pavão das Chagas, Mara Rubia Costa Couri, Jorge Luiz Sônego Milani

**Affiliations:** a Chemistry Institute, 67824 Universidade Federal de Goiás, Av. Esperança, s/n - Campus Samambaia, Goiânia, GO 74690-900, Brazil; b Chemistry Department, Exacts Science Institute, 28113 Universidade Federal de Juiz de Fora, Rua José Lourenço Kelmer, s/n - São Pedro, Juiz de Fora, MG 36036-900, Brazil; c NEQC − Núcleo de Estudos em Química Computacional, Chemistry Department, Exacts Science Institute, 28113 Universidade Federal de Juiz de Fora, Rua José Lourenço Kelmer, s/n - São Pedro, Juiz de Fora, MG 36036-900, Brazil; d CEHTES - Centro de Excelência em Hidrogênio e Tecnologias Energéticas Sustentáveis, Instituto de Química, Universidade Federal de Goiás - UFG, Av. Esperança, s/n - Chácaras de Recreio Samambaia, Goiânia, GO 74690-900, Brazil

## Abstract

A series of 13 pyrazole derivatives, each featuring varied aryl groups in the 3,5-positions, were synthesized and characterized, including the determination of crystalline structures for compounds **11** and **13**. These pyrazoles, in the presence of tetrabutylammonium bromide (TBAB), selectively produced propylene cyclic carbonate (PC), with conversions reaching up to 90% and turnover frequency (TOF) = 75 h^–1^ under optimal conditions (0.4 mol % of **13** and TBAB, 120 °C, 3 h, 30 bar). A 3^3^ Box–Behnken experimental design, with triplicate in the central point, was employed to evaluate the effects of temperature, catalyst, and cocatalyst loading. The nature of the aryl substituent significantly influenced the conversion rates, with electron-withdrawing groups (e.g., NO_2_) yielding higher conversion than electron-donating groups (e.g., Me, MeO). Notably, pyrazoles featuring strong electron-donating *p*-OH-C_6_H_4_ (**11**–**13**) achieved the highest conversions, suggesting that the hydroxyl group also acts as a catalytic site. Density functional theory (DFT) calculations provided insight into the reaction mechanism and energy profiles, highlighting the roles of both the hydroxyl (OH) and amino (NH) groups in the catalytic cycle of compound **13**.

## Graphical abstract

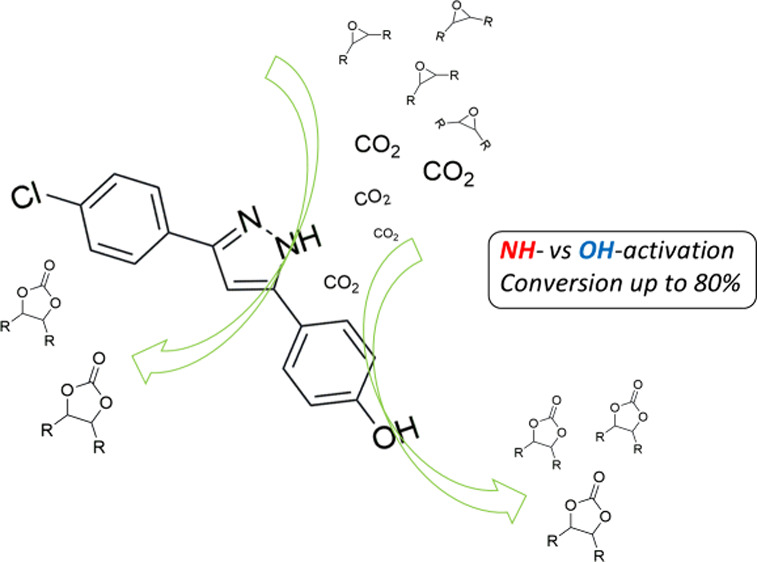

## 1 Introduction

The use of carbon dioxide (CO_2_) as sustainable feedstock has been becoming one of the most challenging tasks in the past years.^1,2^ Therefore, many processes have been developed in order to use CO_2_ as raw material for the production of potentially industrial chemicals and avoid releasing the carbon dioxide at the end of these processes.^3^ This greenhouse, inexpensive and nontoxic gas is already used to produce high-value products such as methanol, formic acid, dimethyl carbonate, and poly and cyclic carbonates.^3,4^ However, the chemical conversion of CO_2_ into valuable chemicals is hampered by its kinetic inertness and thermodynamic stability, which implies, in general, the use of high temperatures and pressures, the employment of high-energy substrates, and/or its need to be used as a catalyst.^4–6^ The production of cyclic organic carbonates (COCs) (Scheme 1) is one of the most viable processes that use CO_2_ efficiently as a feedstock. The COCs are five- or six-membered cyclic carbonates that can be mono- or disubstituted in α and/or β carbons; they can be widely used as polar aprotic solvents, solvents for electrolytes in lithium-ion batteries, monomers for the production of polymers, intermediates in the production of fine chemicals, etc.^7,8^

**Scheme 1:**
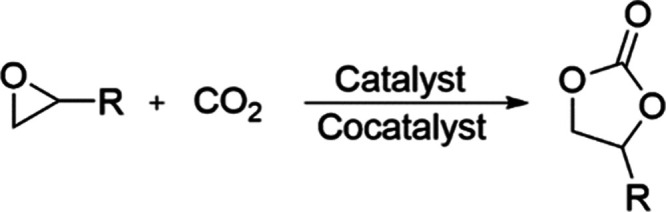
Synthesis of Cyclic Organic Carbonates

Several catalytic systems for CO_2_ cycloaddition reaction are reported in the literature using binary or bifunctional metal-derived catalysts;^4,9–12^ such systems are known due to their high conversion and selectivity. However, to improve sustainability, the development of novel metal-free systems is still a challenge due to the low conversion and, sometimes, poor selectivity and high reactional times when compared to metal catalysts. Based on this, hydrogen bond donor (HBD) catalysts have been attracting the interest of researchers due to the ability of these compounds present to interact with epoxide oxygen, making the epoxide more reactive. In this sense, a large number of HBD has already been investigated, some of them achieving excellent results, such as the ionic liquid derivatives using imidazoles,^13,14^ bis-benzimidazolium,^15^ phosphonium,^16^ triazoles,^17^ or pyrazoles^18^ as the cation portion with different counterions; or in binary systems such as (poly)­phenolic compounds/TBAI,^19^ ascorbic, lactic acids/TBAI,^20,21^ pyridine methanol/TBAX,^22^ and silanediol/TBAI,^23^ which usually present long periods and high catalyst/cocatalyst load reactions.

In light of these, we report the development of a new binary catalytic system using a series of 3,5-*p*-aryl-disubstituted pyrazoles as HBD catalysts. Their catalytic activities were evaluated according to the substitution in both aryl groups and using experimental design studies focused on temperature, catalyst, and cocatalyst amount as variables to be investigated. Also, a theoretical approach was conducted to understand the energy profile of all intermediates and transition states throughout the catalytic cycle.

## 2 Experimental Section

### 2.1 Pyrazole Synthesis

Initially, chalcones were synthesized using commercial aromatic aldehydes and respective commercial acetophenones under basic conditions, using the Claisen–Schmidt reaction (Scheme 2). Then, in a round-bottom flask containing 10 mL of EtOH were added 0.83 mmol of the respective chalcone, 1.00 mmol (0.1862 g) of TsNHNH_2_ and 20 mol % iodine. The mixture was left under magnetic stirring and reflux for 10 min. After that time, 1.25 mmol (0.1725 g) of K_2_CO_3_ was added. The reaction was monitored by TLC (eluent: 100% DCM, revelator: ultraviolet light and I_2_ vapor). After completion, the solvent was removed under reduced pressure, and the mixture was extracted with AcOEt and 10% Na_2_S_2_O_3_ solution. The organic layer was dried with Na_2_SO_4_, filtered, and concentrated under reduced pressure. To the crude was added diethyl ether, diethyl ether/DCM, DCM, DCM/hexane, hexane, or petroleum ether (depends on the pyrazole) to precipitate the compounds. Once precipitated, the products were vacuum filtered affording the desired compounds as pure solids.

**Scheme 2:**
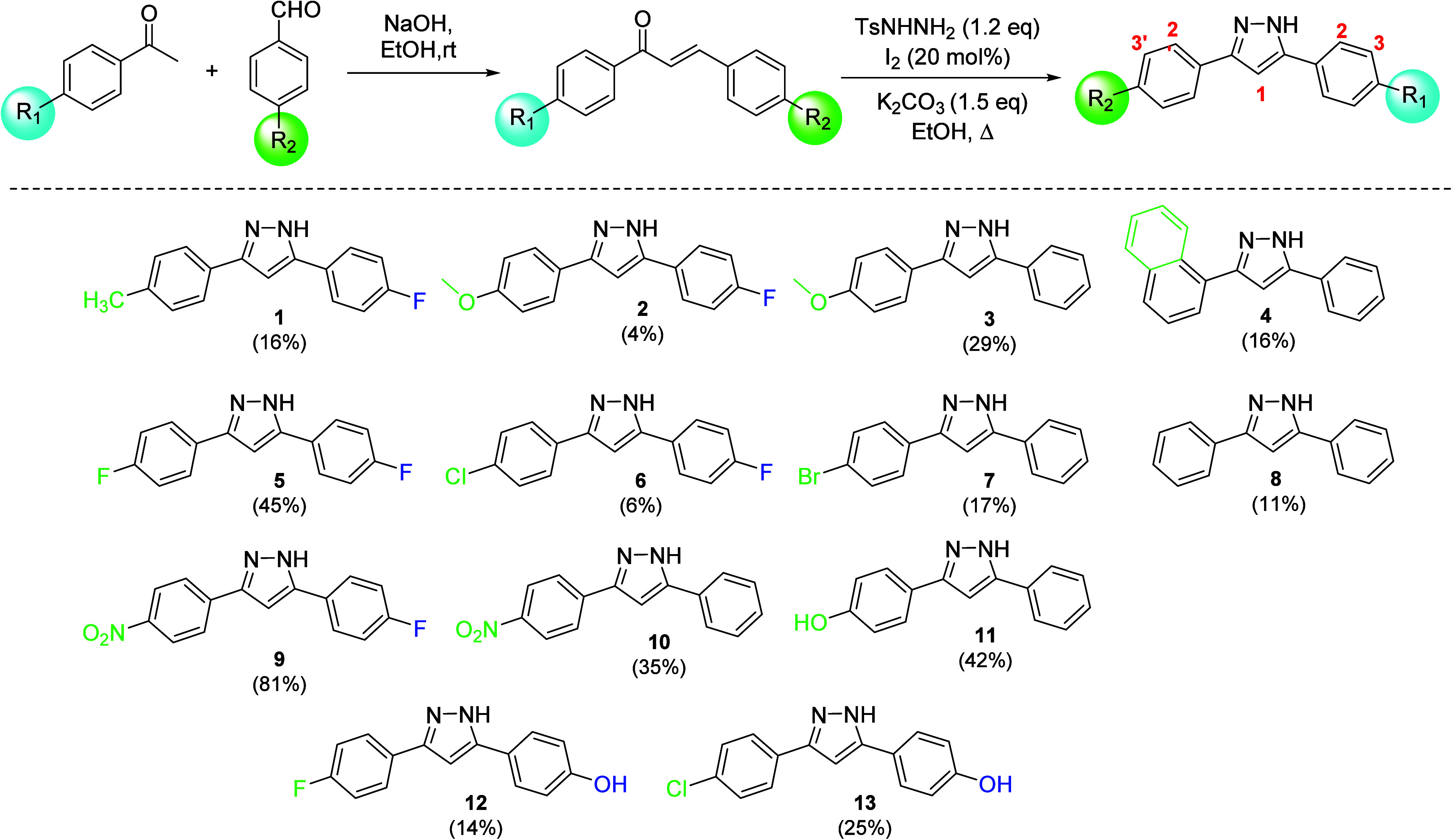
Synthesis of 3,5-Disubstituted Pyrazoles (Yields)

#### 5-(4-Fluorophenyl)-3-(4-methylphenyl)-1*H*-pyrazole (**1**)^24^

^1^H NMR (DMSO-*d*_*6*_; 500 MHz) δ ppm: 13.30 (s, 1H, NH); 7.87 (bs, 2H, H2’); 7.70 (bs, 2H, H2); 7.26 (bs, 4H, H3′ and H2); 7.10 (s, 1H, H1); 2.32 (s, 3H, C*H*_*3*_). ^13^C NMR (DMSO-*d*_*6*_; 125 MHz) δ ppm: 162.7–115.4 (Ar); 99.2 (C1); 20.8 (*C*H_3_).

#### 5-(4-Fluorophenyl)-3-(4-methoxyphenyl)-1*H*-pyrazole (**2**)^25^

^1^H NMR (DMSO-*d*_*6*_; 500 MHz) δ ppm: 13.22 (s, 1H, NH); 7.88–7.85 (m, 2H, H2′); 7.76–7.74 (m, 2H, H2); 7.30–7.26 (m, 2H, H3′); 7.06 (s, 1H, H1); 7.03–7.02 (m, 2H, H3); 3.80 (s, 3H, OC*H*_*3*_). ^13^C NMR (DMSO-*d*_*6*_; 125 MHz) δ ppm: 162.6–114.2 (Ar); 98.8 (C1); 55.2 (O*C*H_3_).

#### 5-Phenyl-3-(4-methoxyphenyl)-1*H*-pyrazole (**3**)^26^

^1^H NMR (DMSO-*d*_*6*_; 500 MHz) δ ppm: 13.22 (s, 1H, NH); 7.82–7.54 (m, 4H, H2′ and H2); 7.43 (bs, 2H, H3′); 7.32 (bs, 1H, H4′); 7.06 (s, 1H, H1); 7.02–7.01 (m, 2H, H3); 3.79 (s, 3H, OC*H*_*3*_). ^13^C NMR (DMSO-*d*_*6*_; 125 MHz) δ ppm: 159.0–114.2 (Ar); 98.9 (C1); 55.2 (O*C*H_3_).

#### 5-Phenyl-3-(2-naphthyl)-1*H*-pyrazole (**4**)^27^

^1^H NMR (DMSO-*d*_*6*_; 500 MHz) δ ppm: 13.51–13.46 (m, 1H, NH); 8.38 (s, 1H, H2); 7.96–7.87 (m, 6H, H2′, H3, H6, H7, and H-8); 7.52–7.33 (m, 7H, H3′, H4′, H4, H-5, and H1). ^13^C NMR (DMSO-*d*_*6*_; 125 MHz) δ ppm: 133.2–123.5 (Ar); 100.0 (C1).

#### 3,5-Di-(4-fluorophenyl)-1*H*-pyrazole (**5**)^28^

^1^H NMR (DMSO-*d*_*6*_; 500 MHz) δ ppm: 13.36 (s, 1H, NH); 7.87 (m, 4H, H2′ and H2); 7.70 (m, 4H, H3′ and H3); 7.20 (s, 1H, H1). ^13^C NMR (DMSO-*d*_*6*_; 125 MHz) δ ppm: 162.7–115.6 (Ar); 99.6 (C1).

#### 3-(4-Chlorophenyl)-5-(4-fluorophenyl)-1*H*-pyrazole (**6**)^28^

^1^H NMR (DMSO-*d*_*6*_; 500 MHz) δ ppm: 13.43 (s, 1H, NH); 7.87 (bs, 4H, H2′ and H2); 7.54–7.49 (m, 2H, H3′); 7.32–7.26 (m, 2H, H3); 7.19 (s, 1H, H1). ^13^C NMR (DMSO-*d*_*6*_; 125 MHz) δ ppm: 162.9–115.4 (Ar); 99.9 (C1).

#### 3-(4-Bromophenyl)-5-phenyl-1*H*-pyrazole (**7**)^29^

^1^H NMR (DMSO-*d*_*6*_; 500 MHz) δ ppm: 13.45 (s, 1H, NH); 7.82–7.79 (m, 4H, H2′ and H2); 7.64–7.63 (m, 2H, H3); 7.45 (t, 2H, *J* = 7.1 Hz, H3′); 7.44 (m, 1H, H4′); 7.21 (s, 1H, H1). ^13^C NMR (DMSO-*d*_*6*_; 125 MHz) δ ppm: 131.7–125.1 (Ar); 99.9 (C1).

#### 3,5-Diphenyl-1*H*-pyrazole (**8**)^30^

^1^H NMR (DMSO-*d*_*6*_; 500 MHz) δ ppm: 13.36 (s, 1H, NH); 7.86–7.81 (m, 4H, H2′ and H2); 7.46 (bs, 4H, H3′ and H3); 7.34 (bs, 2H, H4′ and H4); 7.18 (s, 1H, H1). ^13^C NMR (DMSO-*d*_*6*_; 125 MHz) δ ppm: 157.3–125.0 (Ar); 98.4 (C1).

#### 5-(4-Fluorophenyl)-3-(4-nitrophenyl)-1*H*-pyrazole (**9**)^24^

^1^H NMR (DMSO-*d*_*6*_; 500 MHz) δ ppm: 13.71 (s, 1H, NH); 8.31 (d, 2H, *J* = 8.5 Hz, H3); 8.10 (d, 2H, *J* = 8.5 Hz, H2); 7.88–7.85 (m, 2H, H2′); 7.39 (s, 1H, H1); 7.32 (t, 2H, *J*_’_ = 8.6 Hz, H3′). ^13^C NMR (DMSO-*d*_*6*_; 125 MHz) δ ppm: 162.9–115.8 (Ar); 101.3 (C1).

#### 5-Phenyl-3-(4-nitrophenyl)-1*H*-pyrazole (**10**)^27^

^1^H NMR (DMSO-*d*_*6*_; 500 MHz) δ ppm: 13.72 (s, 1H, NH); 8.31–8.29 (m, 2H, H3); 8.14–8.08 (m, 2H, H2); 7.87–7.81 (m, 2H, H2′); 7.49 (t, 1H, *J* = 7,0 Hz, H3′); 7.44–7.40 (m, 2H, H1 and H4′). ^13^C NMR (DMSO-*d*_*6*_; 125 MHz) δ ppm: 149.4–124.2 (Ar); 101.1 (C1).

#### 5-Phenyl-3-(4-hydroxyphenyl)-1*H*-pyrazole (**11**)^31^

^1^H NMR (DMSO-*d*_*6*_; 500 MHz) δ ppm: 13.11 (s, 1H, NH); 9.61 (s, 1H, OH); 7.81 (d, 2H, *J* = 7.0 Hz, H2′); 7.63 (d, 2H, *J* = 7.8 Hz, H2); 7.43 (t, 2H, *J* = 6.9 Hz, H3′); 7.31 (t, 2H, *J* = 7.1 Hz, H4’); 6.99 (s, 1H, H1); 6.83 (d, 2H, *J*_._= 8.3 Hz, H3). ^13^C NMR (DMSO-*d*_*6*_; 125 MHz) δ ppm: 157.3–125.0 (Ar); 98.4 (C1).

#### 3-(4-Fluorophenyl)-5-(4-hydroxyphenyl)-1*H*-pyrazole (**12**)

^1^H NMR (DMSO-*d*_*6*_; 500 MHz) δ ppm: 13.22 (s, 1H, NH); 9.65 (s, 1H, OH); 7.85 (bs, 2H, H2); 7.62–7.61 (m, 2H, H2′); 7.25 (bs, 2H, H3); 6.97 (s, 1H, H1); 6.84 (d, 2H, *J* = 8.1 Hz, H3′). ^13^C NMR (DMSO-*d*_*6*_; 125 MHz) δ ppm: 162.6–115.6 (Ar); 98.4 (C1). ESI-HRMS: ESI-HRMS: [M + H]^+^: *m*/*z* calculated: 255,0934, found: 255,0595. mp = 209–211 °C.

#### 3-(4-Chlorophenyl)-5-(4-hydroxyphenyl)-1*H*-pyrazole (**13**)

^1^H NMR (DMSO-*d*_*6*_; 500 MHz) δ ppm: 13.18 (s, 1H, NH); 9.72 (s, 1H, OH); 7.84 (bs, 2H, H2); 7.61 (bs, 2H, H2′); 7.47 (bs, 2H, H3); 7.01 (s, 1H, H1); 6.85–6.84 (m, 2H, H3′). ^13^C NMR (DMSO-*d*_*6*_; 125 MHz) δ ppm: 157.5–115.7 (Ar); 98.4 (C1). ESI-HRMS: [M + H]^+^: *m*/*z* calculated: 271,0638, found: 271,1225. mp = 210–211 °C.

### 2.2 Cycloaddition Reactions

Catalytic reactions for the cycloaddition of CO_2_ with epoxides were conducted in a Parr reactor system (model 4560 with controller model 4848) equipped with a 300 mL stainless-steel vessel. For standard cycloaddition reactions, the catalyst (0.2 mmol, 0.4 mol %), cocatalyst (0.2 mmol, 0.4 mol %), and epoxide (50 mmol) were initially placed in the vessel, which was closed and heated to 120 °C before being pressurized with 99.99% CO_2_ to 30 bar. The reaction mixture was then stirred for 3 h and then cooled to 0 °C before releasing the pressure. The conversions were determined by dissolving a sample of the reaction mixture in CDCl_3_ and analyzing it by ^1^H NMR spectroscopy. The total conversion was calculated from the ratio of the integrals of both cyclic carbonate and epoxide methylene signals in the ^1^H NMR spectra (Figures S27–S44).

### 2.3 Computational Details

Kinetics and thermochemical properties are commonly accessed by calculations using density functional theory (DFT). In this work, we have performed different steps of calculations for a full comparison among them. First, a less empirical method has been used, PBEh-3c, by Grimme and collaborators.^32^ Developed in the past decade, this method has been built including short-range effects and providing a better explanation for results encountered in systems like the one studied here. Weigend and Ahlrich’s modified def2-mSVP basis set has also been used for all atoms as included in this 3c method.^33^ Optimization and frequency calculations have been performed for all species throughout a schematic reaction coordinate for 3-(4-chlorophenyl)-5-(4-hydroxyphenyl)-1*H*-pyrazole (**13**) to its final product. Intrinsic reaction calculations (IRC) have also been conducted to connect the transition states with the local minimum structures of the potential energy surface.

Then, Grimme’s dispersion correction effects were included in the calculations. A B3LYP D3 method^34,35^ has been used along with the def2-TZVPP basis set. Optimization and frequency calculations were once again carried out for all species. The results presented in Section 3.3 are based on these calculations.

After optimizations and frequency calculations, a benchmark has been performed to find a better match of electronic energies to validate the method applied above. DFT functionals B3LYP,^36^ TPSSh,^37^ BP86,^38^ and B3PW91 ^39^ were investigated as well as the Pople’s basis set 6-311+G­(2d,p)^40,41^ and Ahlrich’s def2-TZVP. The best one was the Mϕller-Plesset MP2/cc-pVTZ^33,42,43^ level of calculation, which provided a very good match for energies with the inclusion of the CPCM continuum solvation model using the dielectric constant of the epoxide as 2.25.^44^ This whole benchmark and electronics energy analysis is included in the Supporting Information. All calculations have been performed using Orca 5.0.2 release^45^ on Dell servers from NEQC located at the Department of Chemistry, UFJF.

### 2.4 X-ray Data Collection and Structure Determination

The crystallographic data for **9** were collected on a SuperNova, Dual, Cu at home/near, AtlasS2 diffractometer. A well-shaped single crystal was selected for the X-ray diffraction data collection at 293 K under exposition to Cu Kα (λ = 1.54184) radiation. The crystallographic data for **13** were collected on a Bruker AXS Kappa Duo diffractometer with an APEX II CCD detector. A well-shaped single crystal was selected for the X-ray diffraction data collection at 296 K under exposition to MoKα (λ = 0.71073) radiation. The programs SAINT and SADAB^46,47^ were employed for indexing, integrating, and scaling. The crystal structure was solved by direct methods and refined with full-matrix least-squares techniques on *F* ^2^ using SHELXS^47^ programs, respectively, included in the WinGX software package.^48^ All atoms, except hydrogen atoms, were clearly identified and refined by least-squares full-matrix *F* ^2^ with anisotropic thermal parameters. All hydrogen atoms were located in different maps and included as fixed contributions according to the riding model. A molecular graphic was produced with the ORTEP.^48^

## Results and Discussion

The chalcones previously obtained were treated with *p*-toluenesulfonyl hydrazide with molecular iodine as the catalyst. After that, potassium carbonate was further added to the reaction mixture in order to obtain pyrazole derivatives (Scheme 1). All the compounds were precipitated using a suitable solvent or a mixture of solvents.^24^ After these procedures, pyrazoles were characterized by ^1^H and ^13^C NMR and monocrystal diffraction of **9** and **13**.

### 3.1 Crystal Structures of **9** and **13**

Compound **9** was crystallized by slow evaporation of an ethanol solution. Its crystal structure was resolved in orthorhombic space group *Pna*21, with one molecule in the asymmetric unit (Figure 1a). Compound **13** was crystallized by slow evaporation of an ethanol solution. Its crystal structure was solved in monoclinic space group C 2/c with one molecule in the asymmetric unit and one ethanol solvent molecule (Figure 1b). In summary, data on the crystal forms **9** and **13** as well as measurement and processing are shown in Table S1 (SI).

**Figure 1:**
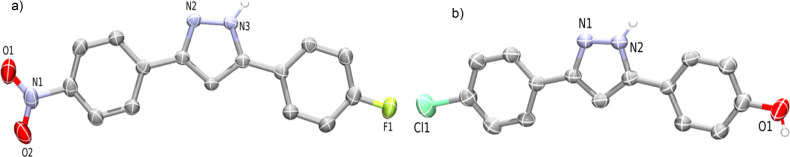
A plot of the asymmetric unit of the crystal forms **9** (a) and **13** (b) with 30% probability anisotropic atomic displacement parameters, with label for fluorine, chlorine, oxygen, and nitrogen atoms (hydrogens were not depicted for drawing clarity).

### 3.2 Experimental Planning

In order to understand the role of variables (temperature, catalyst, and cocatalyst load) in the cycloaddition reaction, a 3^3^ Box–Behnken design with genuine triplicate in a central point was performed. Table 1 depicts the contrast coefficients with coded factors, levels and responses, TON, TOF (h^–1^), and conversion (%). The statistical analysis involving design of experiments was performed in Microsoft Office Excel 2010 software, being considered significance level equal to 0.05 for all calculations carried out.

**Table 1:** 3^3^ Box–Behnken Design with Triplicate in Central Point, Containing Contrasts Coefficients, Factors, Levels, and Responses^a^ ^,^ ^b^

entry	mean	contrast coefficients										responses	
		*X*_1_	*X*_2_	*X*_3_	*X*_1_^2^	*X*_2_^2^	*X*_3_^2^	*X*_1_ *X*_2_	*X*_1_ *X*_3_	*X*_2_ *X*_3_	TON^c^	TOF (h^–1^)^d^	conv. (%)^e^
**1**	1	–1	–1	0	1	1	0	1	0	0	259.1	86.4	25.9
**2**	1	1	–1	0	1	1	0	–1	0	0	662.2	220.8	66.2
**3**	1	–1	1	0	1	1	0	–1	0	0	91.6	30.5	36.6
**4**	1	1	1	0	1	1	0	1	0	0	128.5	60.8	73.0
**5**	1	–1	0	–1	1	0	1	0	1	0	68.7	22.9	17.2
**6**	1	1	0	–1	1	0	1	0	–1	0	166.0	55.3	41.5
**7**	1	–1	0	1	1	0	1	0	–1	0	153.8	51.3	38.5
**8**	1	1	0	1	1	0	1	0	1	0	325.2	108.4	81.3
**9**	1	0	–1	–1	0	1	1	0	0	1	267.4	89.1	26.7
**10**	1	0	1	–1	0	1	1	0	0	–1	85.32	28.4	34.1
**11**	1	0	–1	1	0	1	1	0	0	–1	549.4	183.2	55.0
**12**	1	0	1	1	0	1	1	0	0	1	190.8	63.6	76.3
**13**	1	0	0	0	0	0	0	0	0	0	245.4	81.8	61.4
**14**	1	0	0	0	0	0	0	0	0	0	243.9	81.3	61.0
**15**	1	0	0	0	0	0	0	0	0	0	236.7	78.9	59.2

a *X*_1_—temperature (°C): (−1) 80 °C; (0) 100 °C; (1) 120 °C. *X*_2_—[**13**] (mol %): (−1) 0.10 mol; (0) 0.25 mol %; (1) 0.40 mol %. *X*_3_—[TBAB] (mol%): (−1) 0.10 mol%; (0) 0.25 mol%; (1) 0.40 mol%.

b Reaction conditions: PO (50.00 mmol), *P*_[CO2]_ = 30 bar, *t* = 3 h.

c Turnover number (mol of carbonate produced/mol catalyst).

d Turnover frequency (TON·h^–1^).

e Conversion determined on the basis of ^1^H NMR analysis.

As expected, the experimental design results evidenced the strong temperature dependence (entries 1 (26%) and 2 (66%)) and only moderate dependence with respect to catalyst and cocatalyst load. This trend may be noted in entries 9, 10, and 11 when the temperature was set at 100 °C, and the catalyst and cocatalyst loads were varied from 0.1 to 0.4 mol %. In entry 9, a lower amount of catalyst and cocatalyst (0.1 mol %) was used, producing only 27% of PC. However, by increasing the catalyst amount to 0.4 mol % (entry 10) holding the TBAB load in 0.1 mol %, the system presented a slight positive effect, achieving 34% of conversion; however, when 0.4 mol % of TBAB was used and 0.1 mol % of catalyst, a remarkable positive effect (55% of PC) was noted. These data support the temperature conferred the strongest impact on conversion, followed by cocatalyst load and catalyst load.

After the statistical calculations, taking into account the global responsibility for the catalytic process, the following model was built for response conversion (%), shown in Table S2. All coefficients were significant, except the coefficient *b*_12_, because *p*-value >0.05.

The built model does not present a lack-of-fit evidence within 95% confidence interval, since the *p*-value calculated for the ratio between the sum of squares due to lack of fit and the sum of squares due to pure error was equal to 0.099. The surface response suggests that the high catalyst and cocatalyst load (0.4 mol %) at high temperature would be the best reactional condition, suggesting 89% of conversion, as shown in Figure 2a. To prove this hypothesis, the reaction was carried out using catalyst **13** (0.4 mol % of catalyst and cocatalyst at 120 °C), leading to 90% of conversion, in agreement with those predicted by the surface response suggested. In Figure 2b, the surface response shows that the higher the temperature and cocatalyst amount and the lower the catalyst load, the higher the TOF obtained. The experimental conversion and TOF in *X*_1_ = 1, *X*_2_ = −1, and *X*_3_ = 1 conditions were 74% and 247 h^–1^, which is in good agreement with the predictions in Figure 2b.

**Figure 2:**
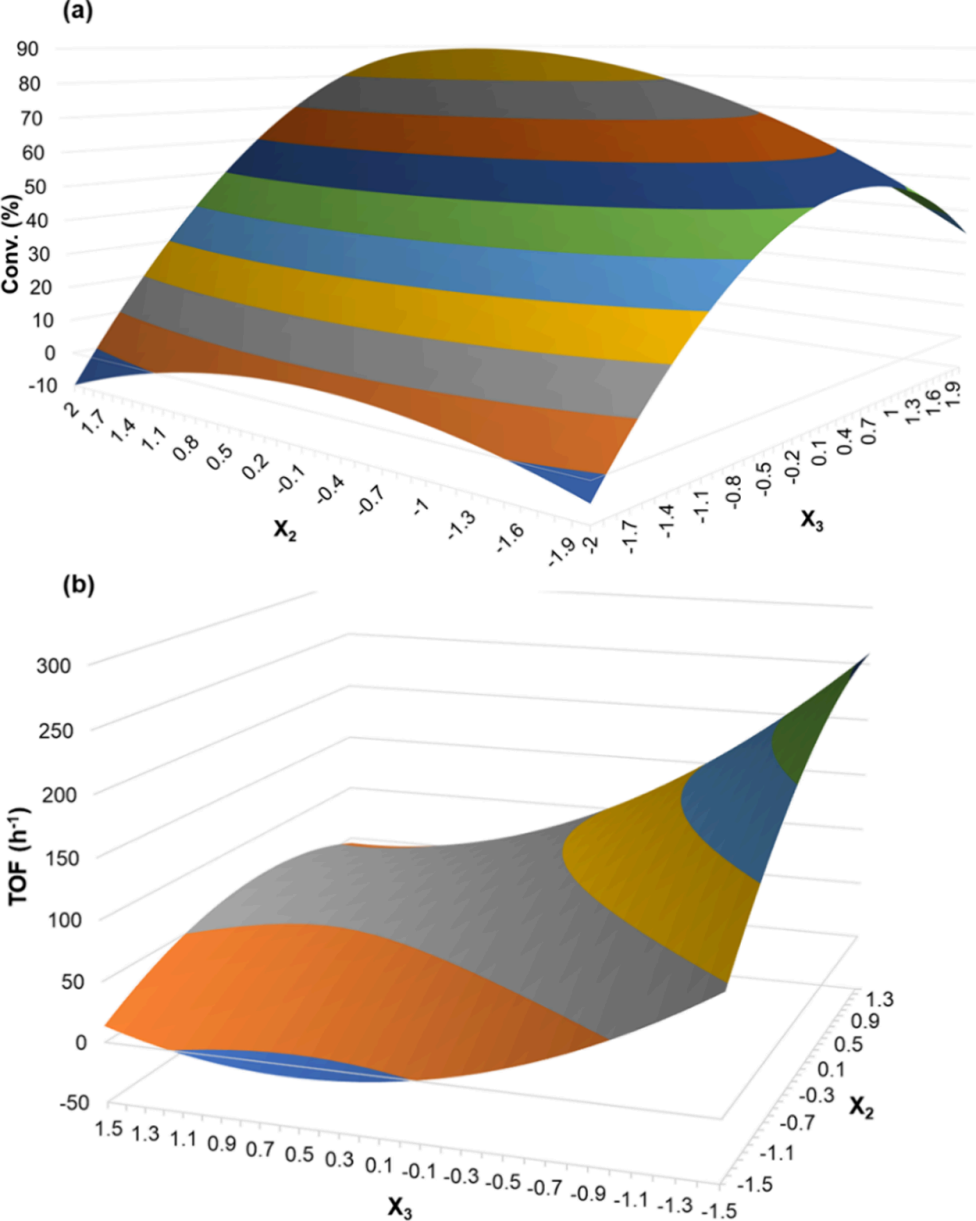
Surface response fitted for the 3^3^ Box–Behnken design for (a) Conv (%) and (b) TOF (h^–1^) considering catalyst (*X*_2_) and cocatalyst (*X*_3_) in temperature fixed at 120 °C.

In compliance with the experimental design testing, the optimized conditions (catalyst (0.4 mol %, TBAB (0.4 mol %) at 120 °C under 30 bar) were defined and various 3,5-aryl-dissubstituted pyrazoles were applied as catalysts in a binary system with TBAB. The results summarized in Table 2 showed that all pyrazoles synergistically with tetrabutylammonium bromide were able to convert propylene oxide (PO) selectively into propylene carbonate (PC) in good to excellent yields. The presence of 3,5-aryl-substituents in the pyrazole heterocycle significantly affected the catalytic performance; pyrazoles bearing donor aryl substituents present lower conversion than pyrazoles bearing electron-withdrawing groups. Such behavior can be rationalized according to *pK*_a_ values of N–H proton; lower *pK*_a_ values interact strongly with epoxide oxygen, being more effective in the epoxide activation.^7,21,49,50^

**Table 2:** Cycloaddition of PO and CO_2_ Catalyzed by **1**–**13**/TBAB^b^

entry	catalyst	conversion (%)^c^	TON^d^	TOF (h^–1^)^e^
1	[Table-fn t2fn6]	19		
2	**1**	44	108	38
3	**2**	64	161	54
4	**3**	70	176	59
5	**4**	74	184	61
6	**5**	75	186	62
7	**6**	76	189	63
8	**7**	78	195	65
9	**8**	79	198	66
10	**9**	82	205	68
11	**10**	85	214	71
12	**11**	87	217	72
13	**12**	90	225	75
14	**13**	90	227	75

a Reaction condition: 0.2 mmol of catalyst and TBAB (0.4 mol %), PO (50.00 mmol), *T* = 120 °C, *P* = 30 bar, *t* = 3 h.

b Reaction condition: conversion determined on the basis of ^1^H NMR analysis.

c Reaction condition: turnover number (mol of carbonate produced/mol catalyst).

d Reaction condition: turnover frequency (TON·h^–1^).

e Reaction condition: 0.4 mol % of TBAB.

Even though the conversions presented a modest difference among the catalysts, it was evidenced that the donor groups, such as Me or OMe, had the worse conversions (entries 2, 3 and 4); catalyst **1** was poorly soluble in reactional media, and its conversion was only 44%, while catalysts **2** and **3** converted 64 and 70% of PO, respectively. Replacing the donor groups with anthracene (**4**) or hydrogen (**8**), the conversion increased moderately to 74 and 79%, respectively. Aryl substituents bearing electronegative halogens (**5**, **6**, or **7**) afforded an intermediate conversion ranging from 75 to 78%, suggesting their weak influence on reaction conversion. However, when the NO_2_ group was tested, a notable positive effect was observed, increasing the conversion rates to 82 (**9**) and 85% (**10**) of PC, which supports the hypothesis that lower *pK*_a_ values correlate with higher conversion rates. This behavior is evident when comparing entries 2 (Me/F), 3 (OMe/F), and 4 (OMe/H) with entries 9 (NO_2_/F) and 10 (NO_2_/H). Additionally, three other pyrazoles (**11**, **12**, and **13**) bearing a *p*-HO-aryl group were prepared. Their catalytic performance unexpectedly showed the highest conversions (87, 90, and 90%) even with the strong mesomeric donor hydroxyl group, suggesting that the phenol group can also activate the epoxide.^19,22,50,51^ Theoretical investigations elucidated the mechanism when these two active groups (NH and OH) are present (Section 3.3 and Figure 3).

**Figure 3:**
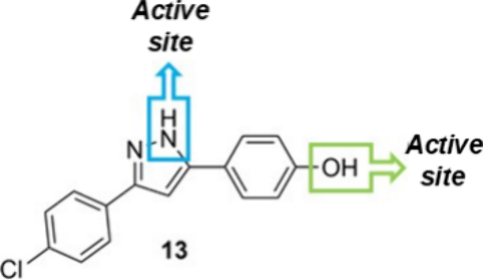
Bro̷nsted-active sites in catalyst **13**.

Due to the industrial significance of various cyclic carbonates, we evaluated the versatility of the system with different epoxides using **13**/TBAB (Figure 4). All epoxides achieved high conversions (>80%) to their respective cyclic carbonates without any loss of selectivity. Epoxides bearing alkyl chains exhibited lower conversions compared to those with electron-withdrawing groups. This difference may be attributed to the poor solubility of the catalyst in alkane-bearing epoxides and/or the high electronegativity of heteroatoms favoring the ring-opening step.

**Figure 4:**
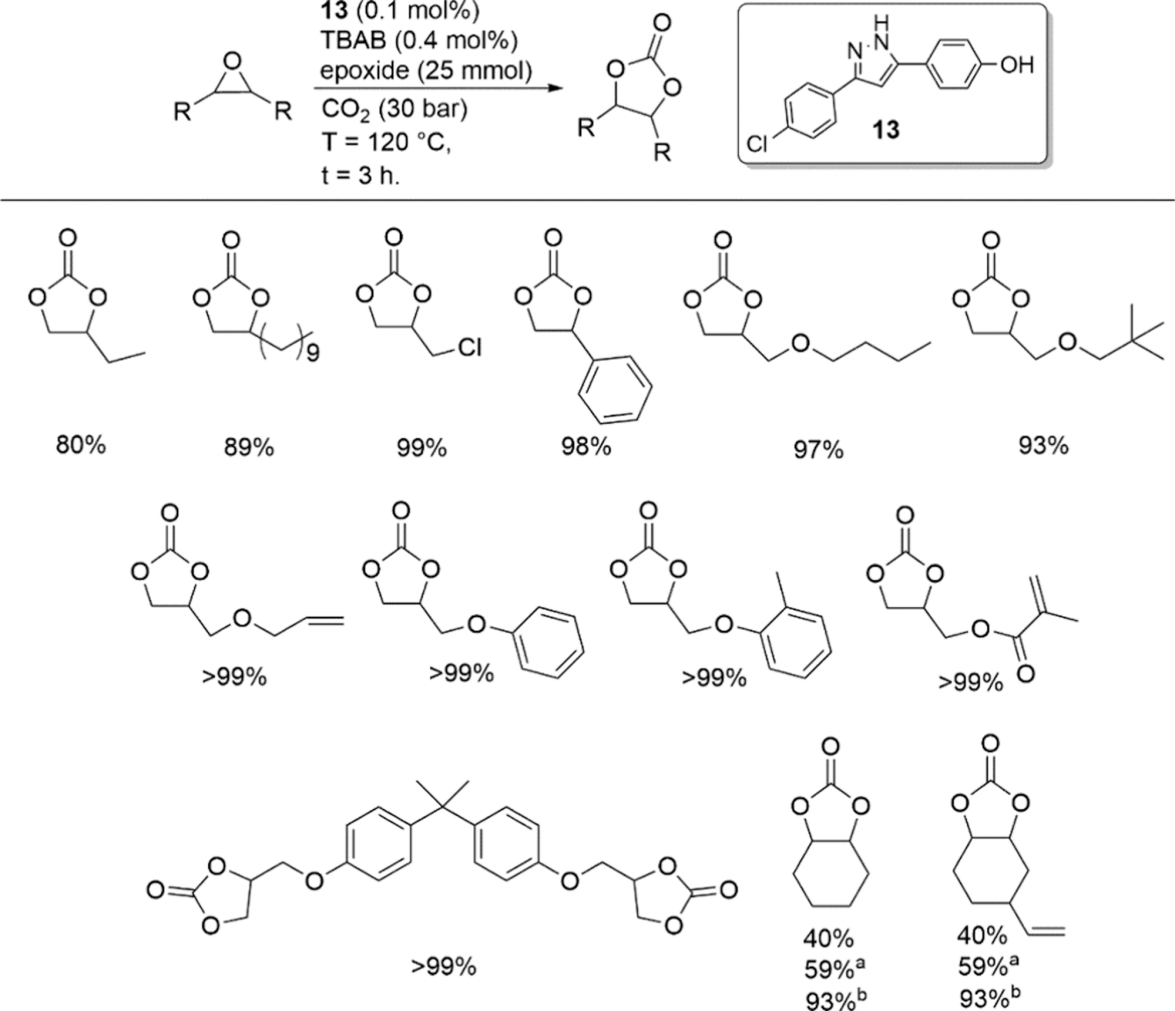
Synthesis of various carbonates using compound **13**. ^a^ *t* = 6 h. ^b^ *t* = 24 h.

Furthermore, under the same conditions, cyclohexene oxide yielded 40% cyclohexene carbonate initially, with extended reaction times of 6 and 24 h resulting in 59 and 93% yields, respectively. Comparatively, our system demonstrated superior performance compared to reactions using polyphenol lignin/KI^51^ (20%, 140 °C, 36 h) or ascorbic acid/TBAI (88%, 100 °C, 23 h),^20^ while employing lower amounts of catalyst and cocatalyst (1.3 mol % lignin (−OH)/4 mol % TBAI and 2 mol % ascorbic acid/4 mol % TBAI). These high conversions motivated further investigation into other disubstituted epoxides, such as 4-vinyl-1-cyclohexene-1,2-epoxide, which achieved 76% conversion in 24 h.

### 3.3 Theoretical Investigation

The understanding of the catalytic mechanism pathway can only be fully investigated by theoretical calculations since there are many species like transition states that may not be experimentally detected. D’Elia et al.^21^ have previously conducted an extensive study linking p*K*_a_ values to conversion rates in cycloaddition reactions, exploring a range of hydroxyl derivative catalysts. Their work established that within the p*K*_a_ range of 9 to 11, the highest conversions were observed. Stronger acids were found to promote proton transfer from the hydrogen bond donor (HBD) to intermediate species, whereas weaker acids exhibited lesser ability to effectively activate the epoxide. In our work, from experimental data, the presence of two active sites (NH and OH, 3) capable of activating the epoxide ring was hypothesized. Then, DFT calculations were conducted to provide a comprehensive understanding of the mechanistic pathway, particularly regarding OH or NH activation during the reaction. A plausible catalytic cycle is depicted in Figure 5, starting from a more active 3-(4-chlorophenyl)-5-(4-hydroxyphenyl)-1*H*-pyrazole (**13**) catalyst. Figure 5 displays the OH-activation pathway to the left and that via NH to the right. Both have the same corresponding structures.

**Figure 5:**
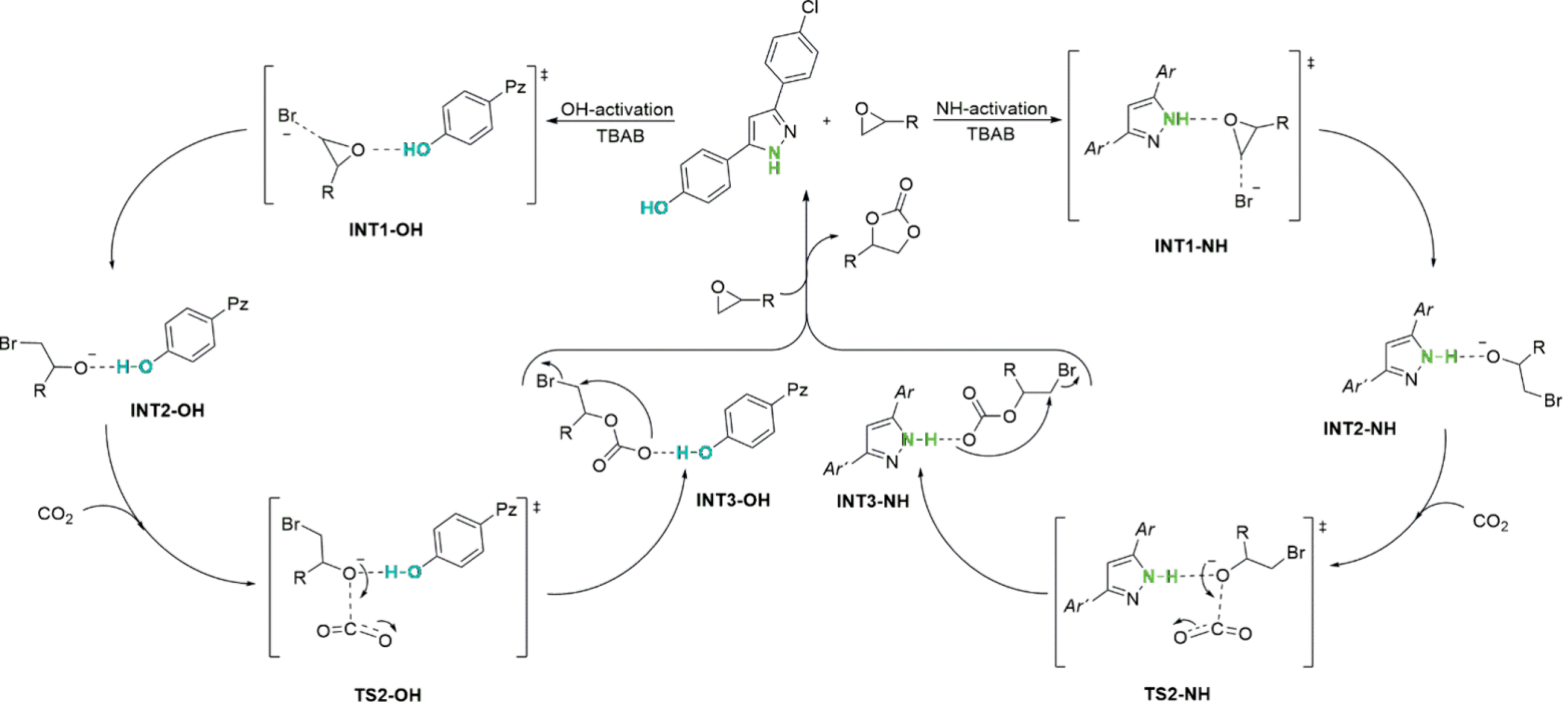
General conceptualization of the catalytic cycle of pyrazole **13**.

A theoretical analysis was conducted based on DFT calculations using compound **13** as a model for studying the potential energy surface (PES) throughout the reaction pathway. The Grimme’s modified functional PBEh-3c as displayed by Grimme and co-workers^32^ has been first used as previously stated on Section 2.3. Then, we have included the dispersion correction effects for a better description of the PES; Gibbs free energy of each species has been evaluated and provided in the profile shown in Figure 6.

**Figure 6:**
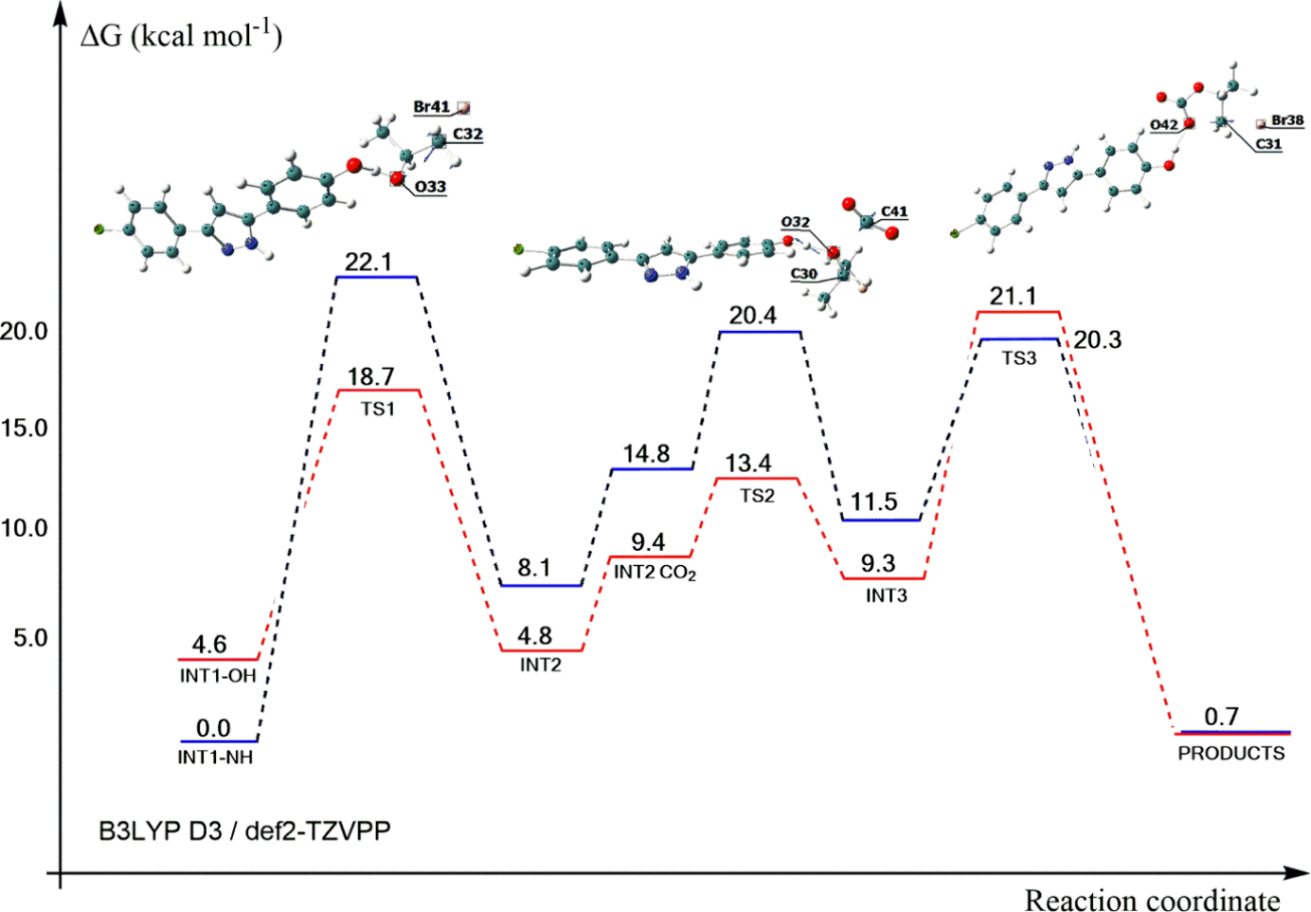
Potential energy surface given by Gibbs free energy variation along the reaction coordinate. NH active site reaction is displayed in blue, while the OH active site is in red. The optimized structures of the transition states for the OH pathway is displayed. Each plateau corresponds to one species in the catalytical cycle originally related to **13**.

In Figure 6, the first step describes the reaction from intermediate INT1 through transition state TS1 (the first barrier). The bromide anion (from TBAB) interacts with epoxide in the less substituted carbon to open the ring, generating the TS1 structure (see Figure S59). It is possible to notice that the energy barrier for the reaction via the OH active site (14.1 kcal mol^–1^) is remarkably smaller than the one via the NH active site (22.1 kcal mol^–1^), suggesting a faster ring opening by the OH site. Both TS1-OH and TS1-NH show the proton transfer, generating an equilibrium between the alkoxide and the less reactive halohydrin at INT2-OH and INT2-NH, respectively. By molecular approaching of CO_2_, catalyst **13** is reestablished by capturing the proton back from halohydrin; then, alkoxide can interact with CO_2_ to achieve intermediate species INT2-CO_2_. From INT2-CO_2_ to INT3, carbonate is formed. It is possible to note that TS2 has the smallest barriers.

The last step shows a barrier of 11.8 kcal mol^–1^ from INT3 to TS3, illustrating the ring closure as the carbon gets closer to the oxygen (O42) and the bromide anion goes away, leading to the formation of the product. The very same path has also been investigated for the NH active site; in this case, the Δ*G* ^‡^ for INT3-NH → TS3-NH is 8.8 kcal mol^–1^. It is plausible to affirm that the low energy difference between INT3 → TS3 (OH and NH; ΔΔ*G* ^‡^ = 3.0 kcal mol^–1^) and INT1 → TS1 (OH and NH; ΔΔ*G* ^‡^ = 8.0 kcal mol^–1^), which indicates that the first step is the driving force responsible for the NH activation being the preferable pathway for the reaction. However, the lower activation barrier from INT1-OH to TS1-OH suggests that both pathways may coexist in the system at a given temperature. All species are displayed in the Supporting Information (Figures S47–S55). Also, one can find the coordinates for each geometry optimized (PBEh-3c) in this work and the three graphics obtained from the IRC calculations (Figures S56–S58).

We have also performed MP2 single-point calculations of Δ*E* (electronic energies) for the studied barriers, indicating the same pattern observed in Figure 6. The discussion and correlation between MP2 electronic energy and the one from PBEh-3c frequency calculations are included in the Supporting Information (please refer to Figure S60 and Table S3). It is necessary to say that the agreement is good considering a comparison between a post-HF method with DFT.

Furthermore, the comparison of absolute Gibbs energies for PBEh-3c and B3LYP D3, directly obtained from frequency calculations, could also be conducted. Here, there are two major points to consider: the inclusion of solvent effects and the dispersion correction in the B3LYP D3 level of theory. For such comparison, Pearson’s determination coefficient was calculated as 0.876. This means that the inclusion of solvent effects and Grimme’s D3 correction might be responsible for a better description of the potential energy surface (Figure S60).

In addition to energetic analysis, a structural investigation of the calculated species involved in the proposed mechanism is essential. Particular attention is given to the two key steps in the mechanism corresponding to the first and third energy barriers. The structural analysis focuses on two critical bonds: (i) C32–Br, which shows the interaction between the bromide and a specific carbon atom of the epoxide during the first barrier, and (ii) C32–O33 bond, which shows the ring-opening process. These bonds were analyzed by both OH and NH active sites. Figure 7 depicts the bond behavior across the INT1, TS1, and INT2 species, providing a comparative analysis.

**Figure 7:**
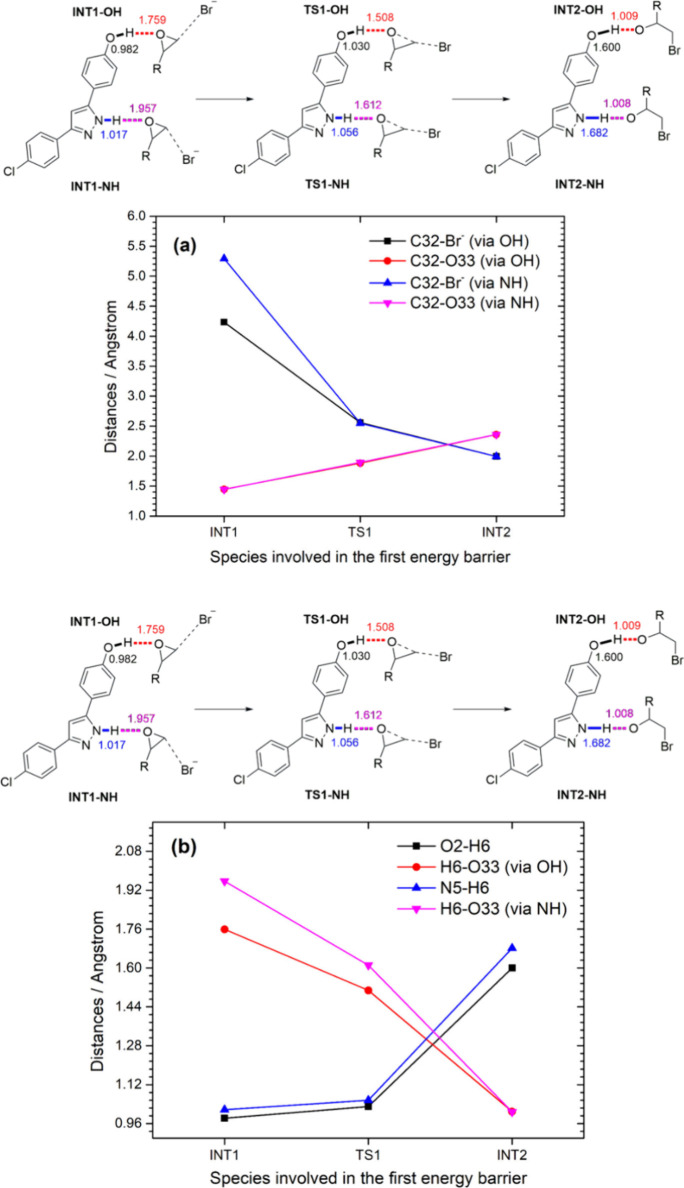
Distances comparisons (in Angstroms) for (a) both C32–Br and C32–O33 (epoxide) bonds on OH and NH active sites; the red and pink segments are quite similar and therefore superposed; (b) proton transfer via OH and NH active sites.

Figure 7 shows the approach of bromide toward epoxide, inducing its opening while the phenolic proton (or the pyrazolic one) is transferred to the alkoxide (H39). Distances between (N5–H6)···O33 and (O2–H39)···O33 (Figure 7b; pink and red lines, respectively) suggest a stronger interaction between O33 from epoxide and the phenolic proton when compared to the one from NH as the distances in INT1 and TS1 are closer to the phenolic proton than to the pyrazole one. This sequence of events is further illustrated in Figure S59, where the optimized structures of INT1, TS1, and INT2 for the OH active site pathway are displayed. The larger distance of C32–Br in INT1 is due to its conformation relative to the positions of the epoxide and bromide in TS1 and INT2. However, this rearrangement does not compromise the energetic barrier, which remains lower than that of the NH catalytic site.

## Conclusions

In summary, the effectiveness of a series of 3,5-aryl-substituted pyrazoles as hydrogen bond donor catalysts for the conversion of CO_2_ and epoxides into cyclic carbonates was reported. Employing a binary system using TBAB as a cocatalyst (at a 1:1 cat:cocat ratio), it achieved conversions ranging from good to excellent, with 100% selectivity. A 3^3^ Box–Behnken design revealed temperature as the most critical factor, followed by the cocatalyst and the catalyst itself. Through experimental optimization, the optimal conditions were defined (catalyst: 0.4 mol %, TBAB: 0.4 mol %, at 120 °C under 30 bar). Applying 13 different pyrazoles as catalysts in the CO_2_ cycloaddition reaction, the conversion rates were closely linked to the nature of aryl groups at the 3,5-positions. Electron-withdrawing groups enhanced activity by lowering the NH proton p*K*_a_, facilitating the epoxide activation, whereas electron-donating groups reduced conversion. Interestingly, pyrazoles bearing OH-donor groups (**12** and **13**) showed markedly improved conversions. DFT calculations provided further insights, confirming that the introduction of a new hydrogen bond donor (−OH) into the catalyst structure positively influenced the conversion by serving as a secondary activation site for the epoxide.

## Supplementary Material



## Notes

Characterization and all experimental procedures for pyrazole synthesis; analytical data as ^1^H and ^13^C NMR spectra of all pyrazoles and cycloaddition reactions as well; data of DRX analysis for crystalline structures of **9** and **13**; and data of theoretical data used to prove the reaction mechanism (PDF)
