# Supplementary material for: Influence of Donor/Withdrawing Groups in an 3,5-Aryl-Substituted Pyrazole Organocatalyst for the Chemical Fixation of CO2
Source: ACS Omega. 2025 Jun 6;10(23):24224–34. doi: 10.1021/acsomega.4c11307 (PMC12177759; doi:10.1021/acsomega.4c11307)
Supplement: Supplementary file 1 [file ao4c11307_si_001.pdf]

Electronic supplementary material for

Influence of donor/withdrawing groups in 3,5-aryl-substituted pyrazoles organocatalyst for the chemical fixation of CO_2

Gabriel Elias Taliateli Oliveira Prado, Karine Braga Enes, Álvaro Farias Arruda da Mata, Gabriel Cerqueira, Marcone Augusto Leal de Oliveira, Luiz Antônio Sodré Costa, Felipe Terra Martins, Meiry Edivirges Alvarenga, 2 Rafael Pavão das Chagas, Mara Rubia Costa Couri, Jorge Luiz Sônego Milani**

Figure S1. ^{1}H NMR spectrum of the compound **1** (500 MHz, DMSO-d_6)

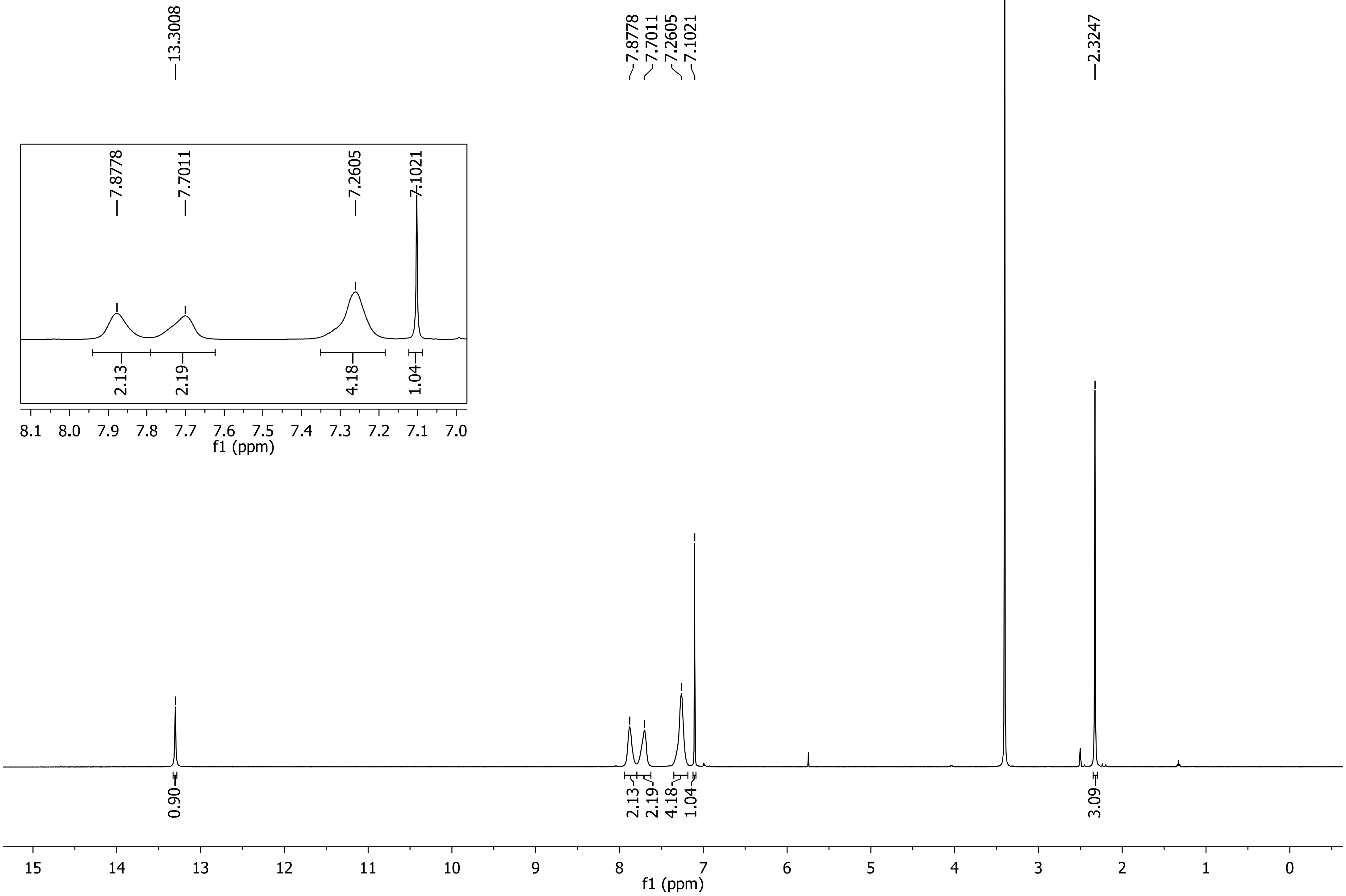

Figure S2. ^{13}C NMR spectrum of the compound **1** (125 MHz, DMSO-d_6)

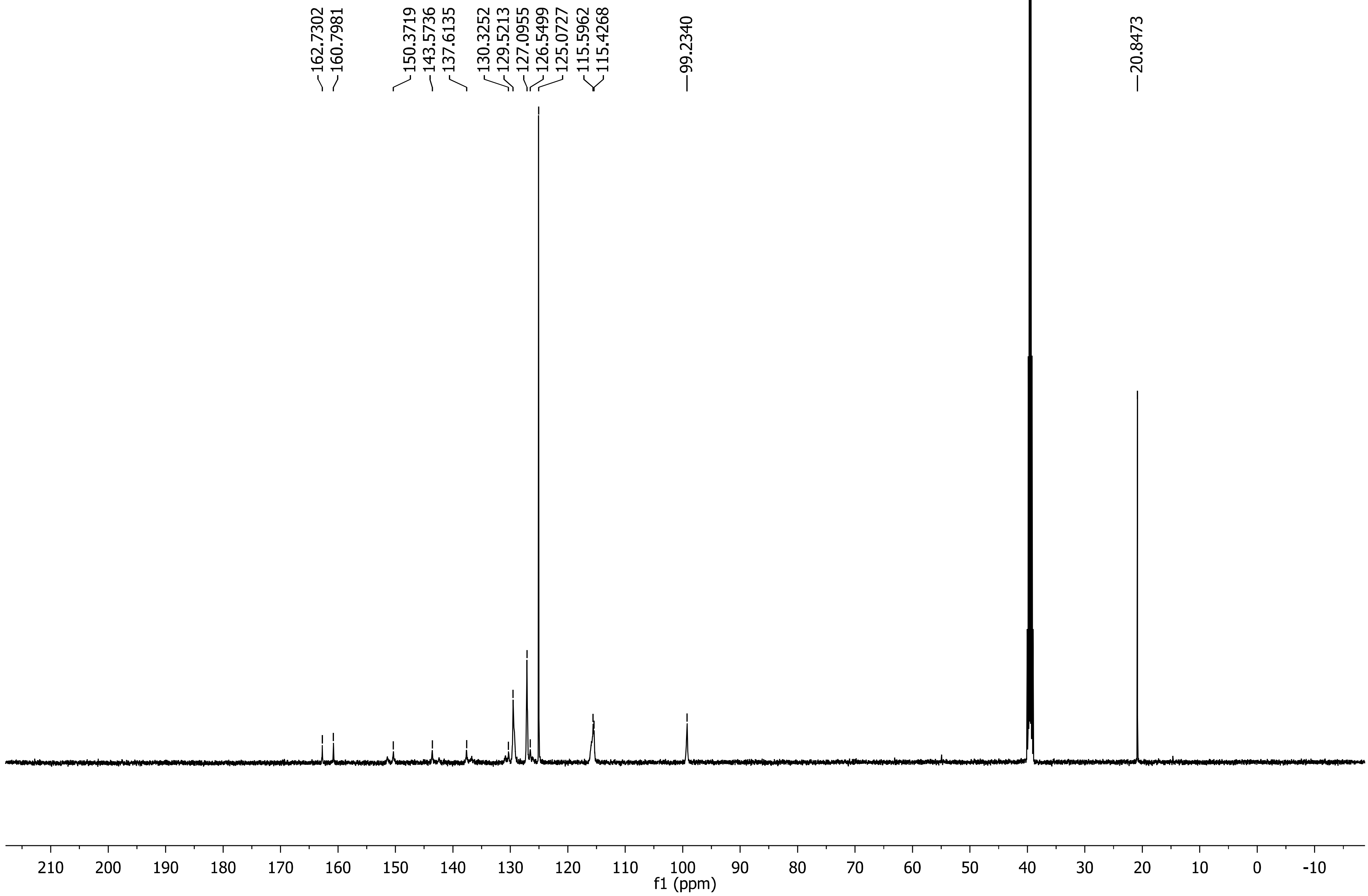

Figure S3. ^{1}H NMR spectrum of the compound **2** (500 MHz, DMSO-d_6)

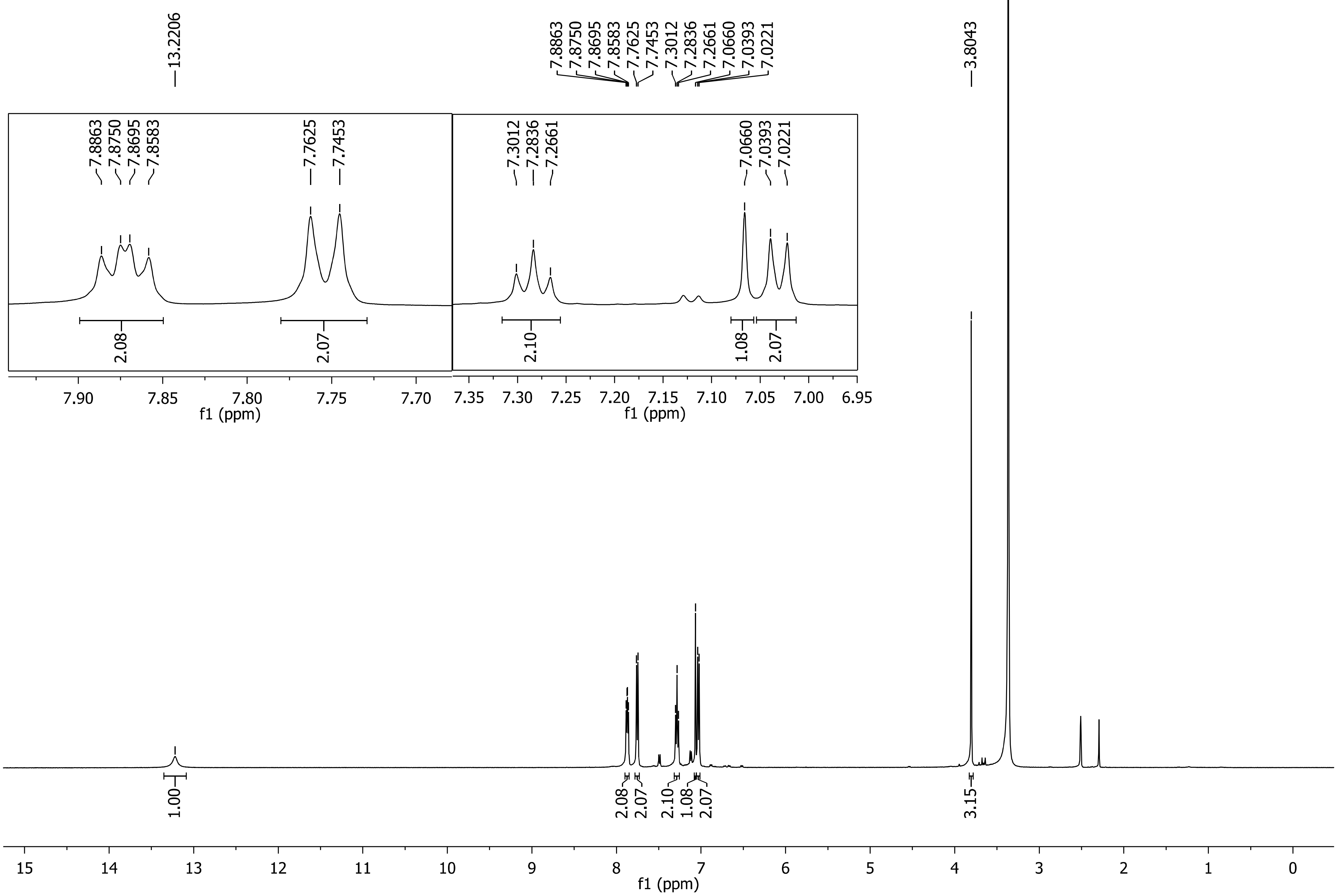

Figure S4. ^{13}C NMR spectrum of the compound **2** (125 MHz, DMSO-d_6)

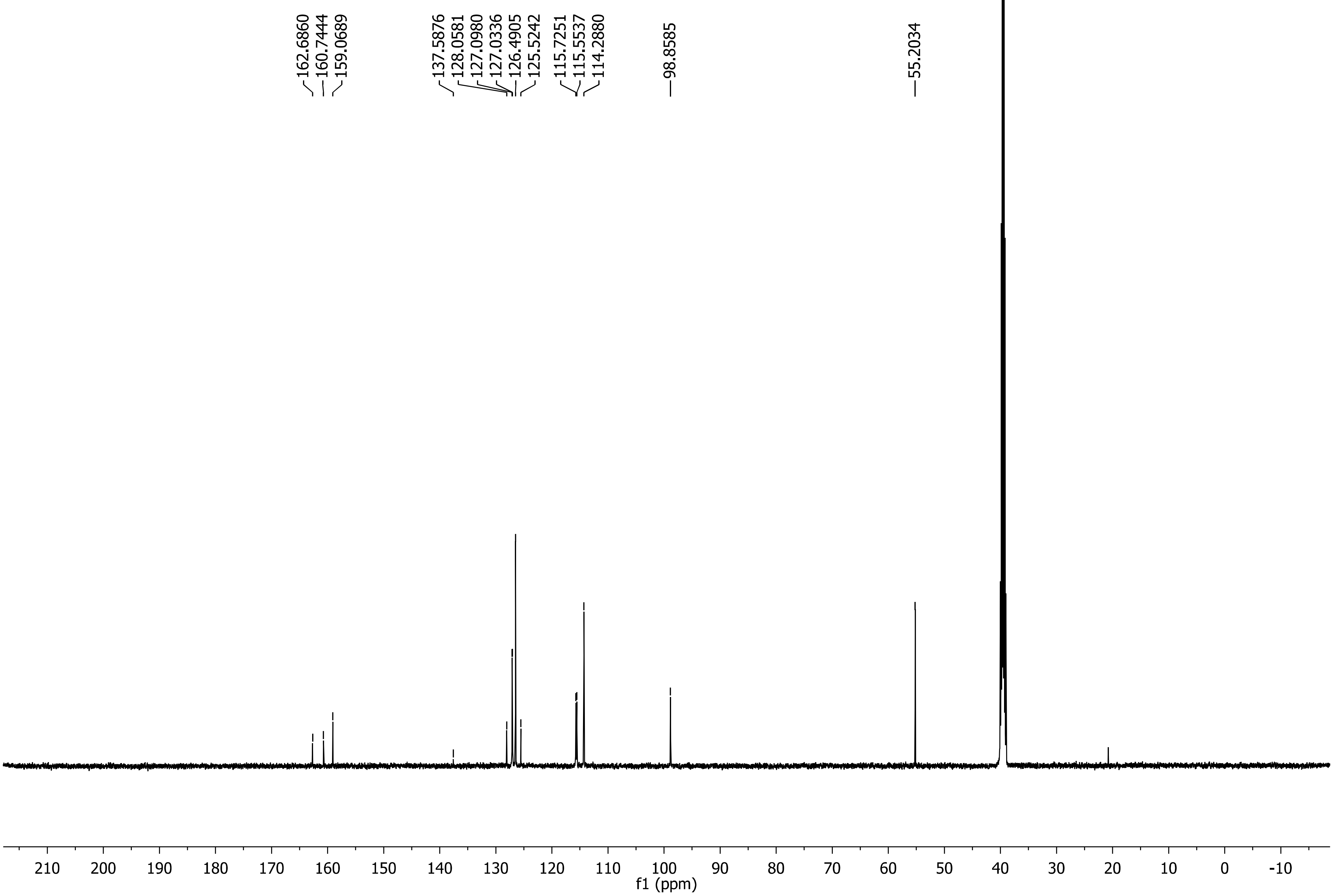

Figure S5. ^{1}H NMR spectrum of the compound **3** (500 MHz, DMSO-d_6)

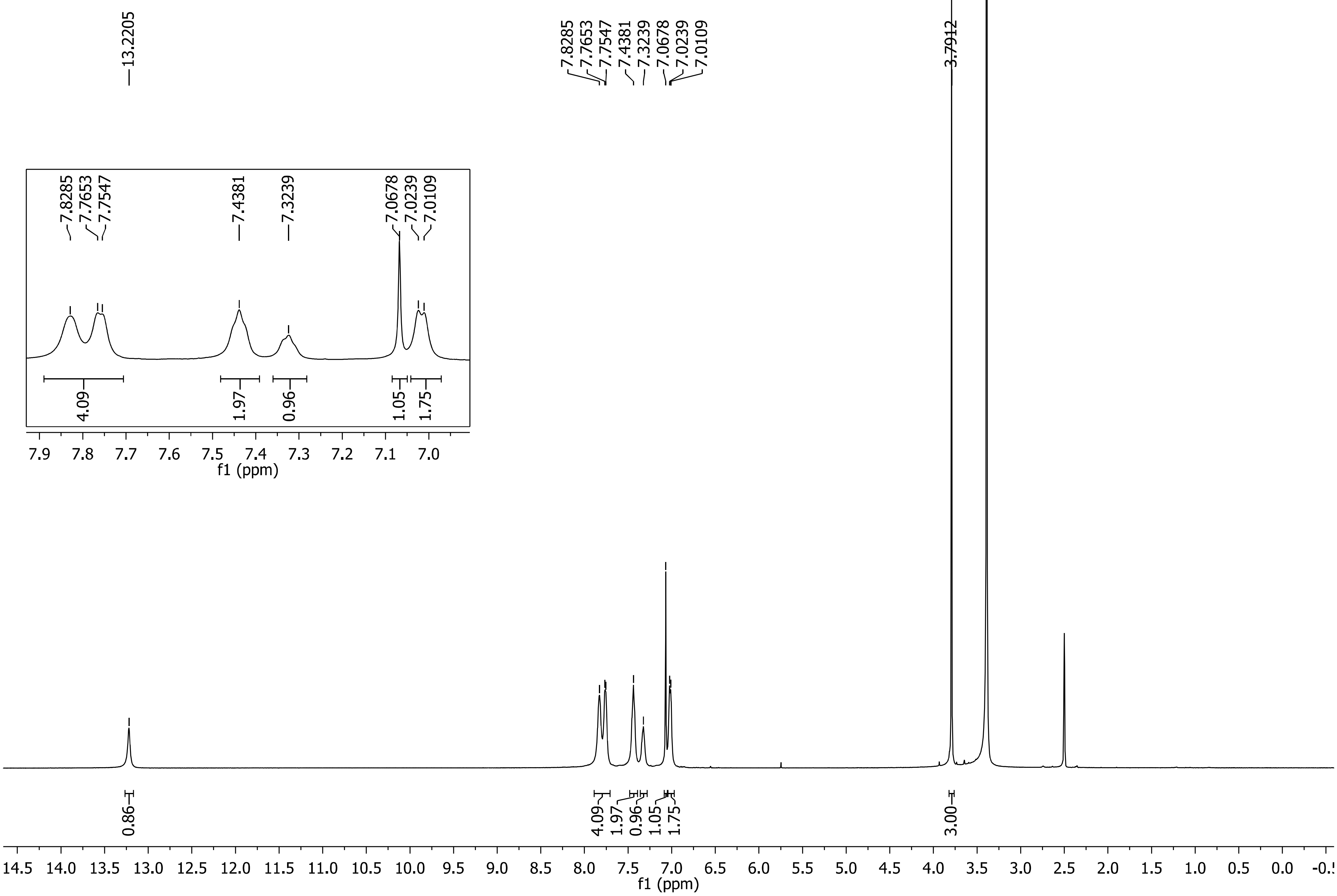

Figure S6. ^{13}C NMR spectrum of the compound **3** (125 MHz, DMSO-d_6)

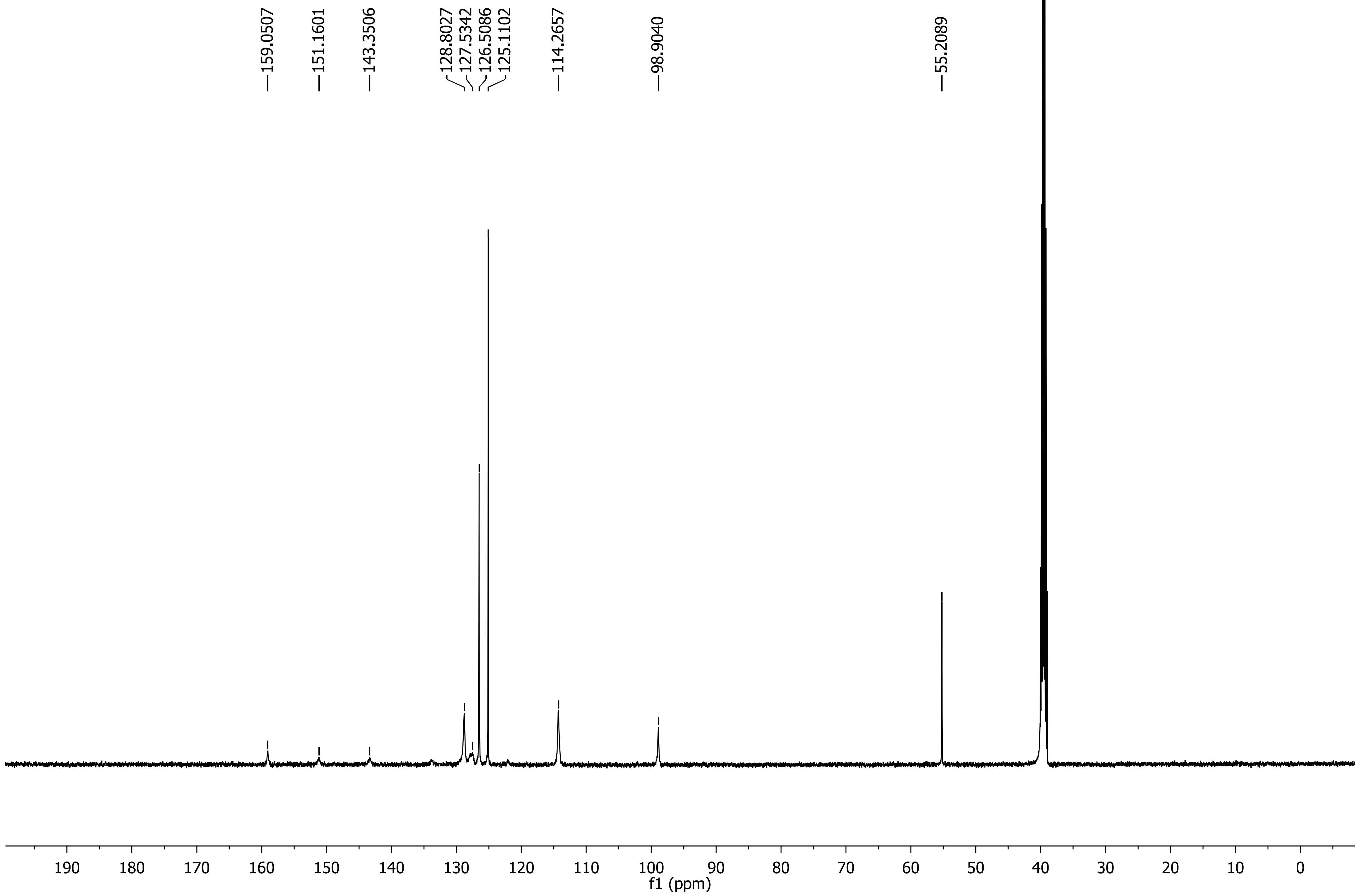

Figure S7. ^{1}H NMR spectrum of the compound **4** (500 MHz, DMSO-d_6)

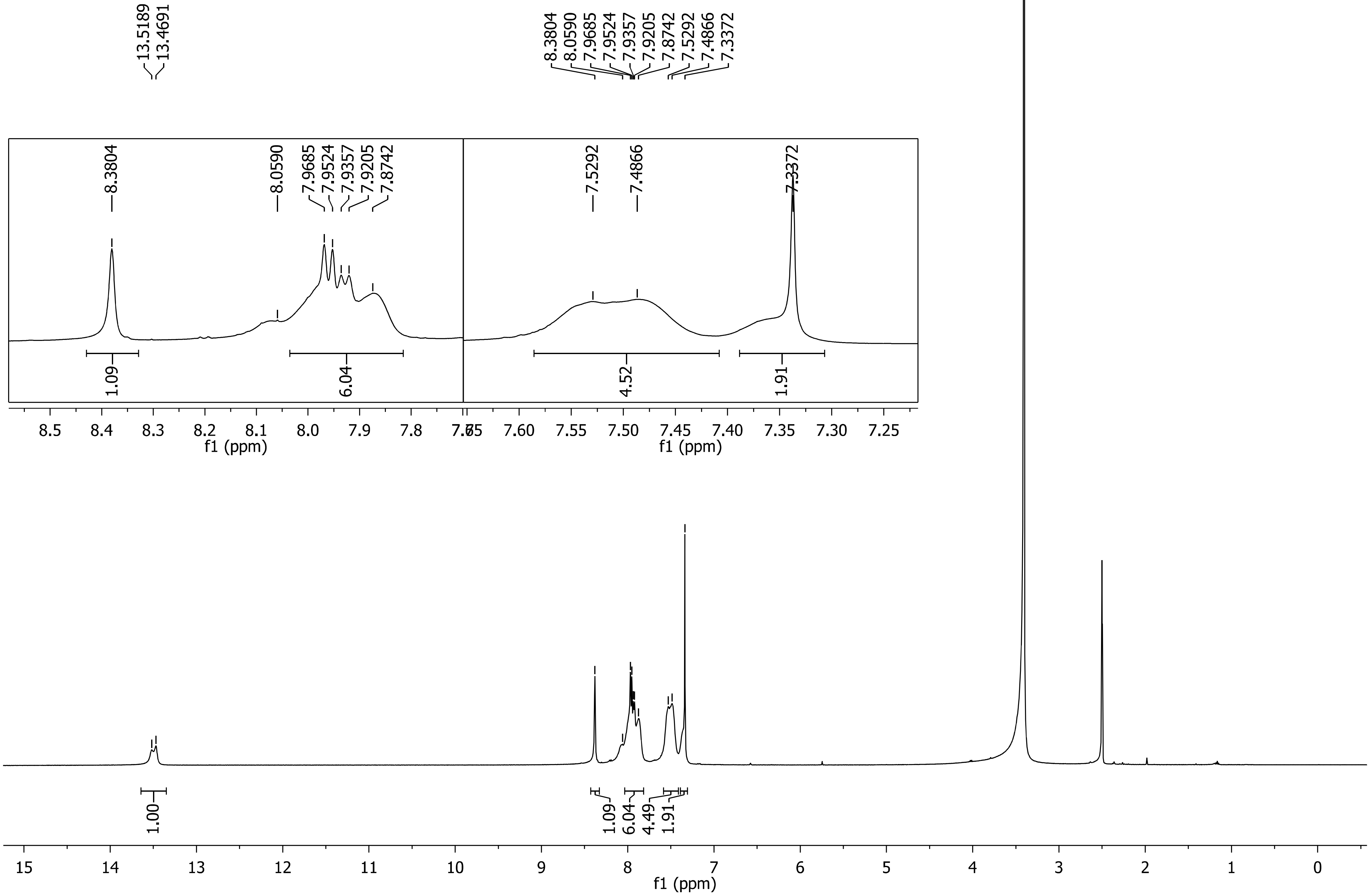

Figure S8. ^{13}C NMR spectrum of the compound **4** (125 MHz, DMSO-d_6)

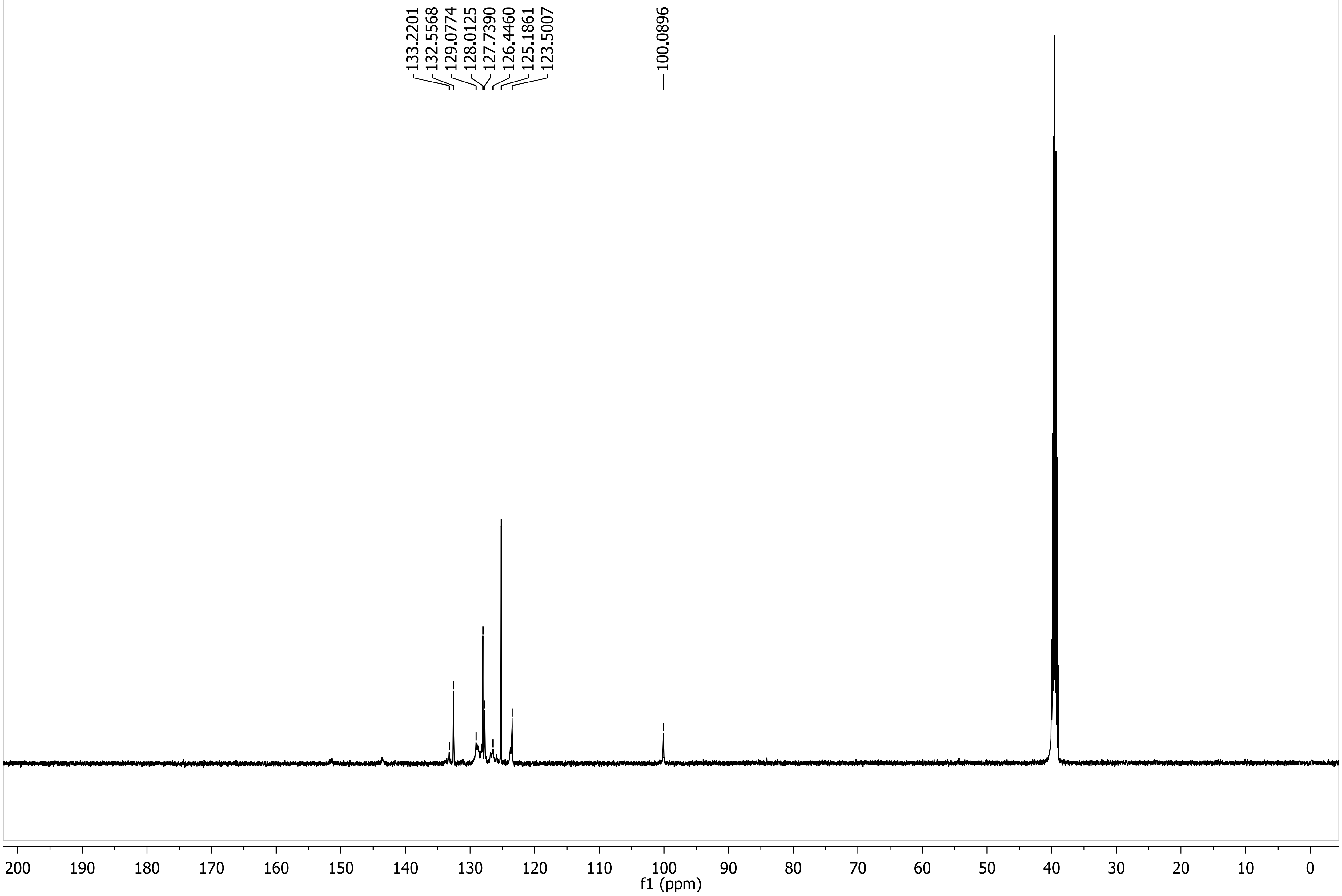

Figure S9. ^{1}H NMR spectrum of the compound **5** (500 MHz, DMSO-d_6)

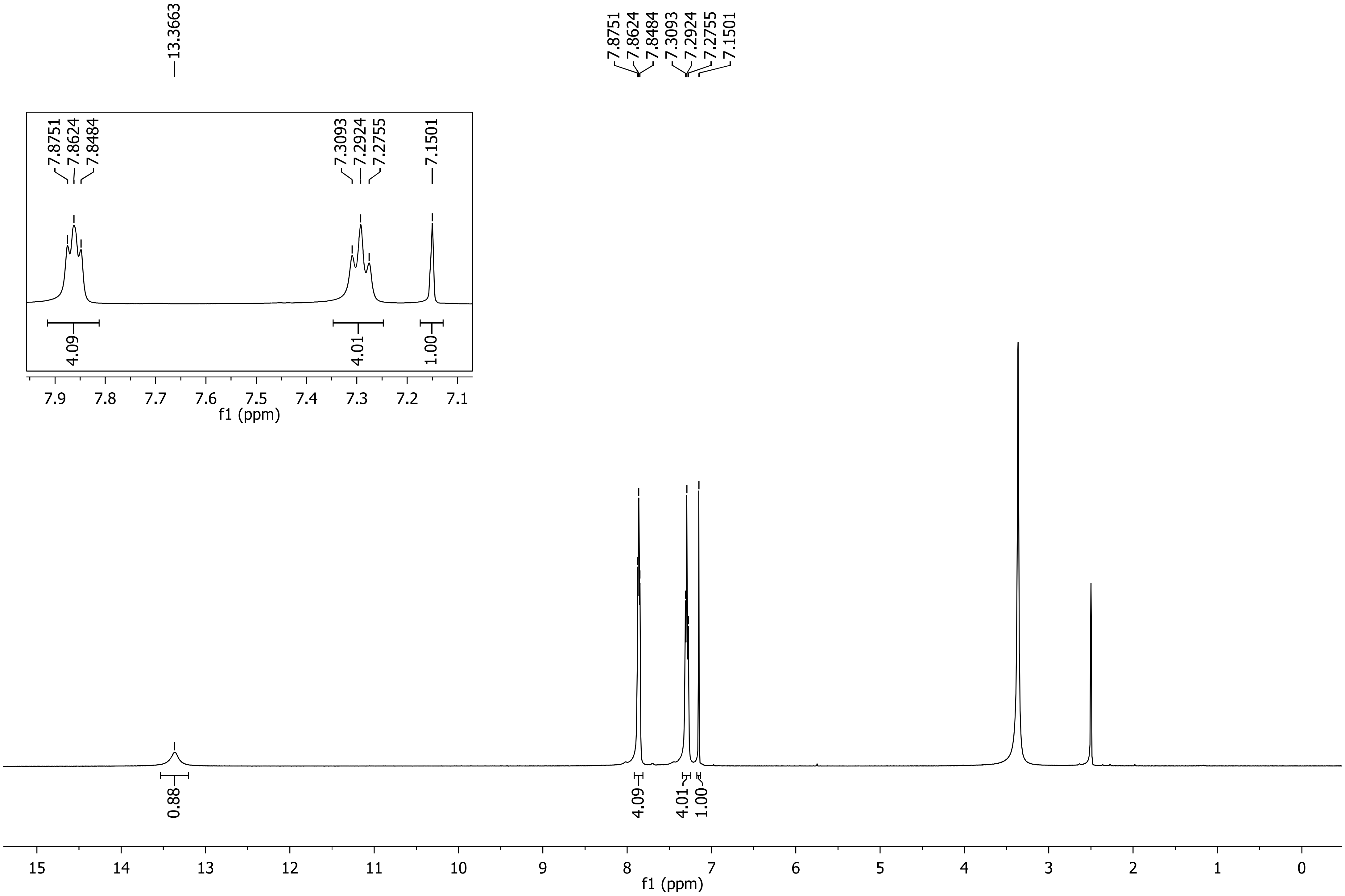

Figure S10. ^{13}C NMR spectrum of the compound **5** (125 MHz, DMSO-d_6)

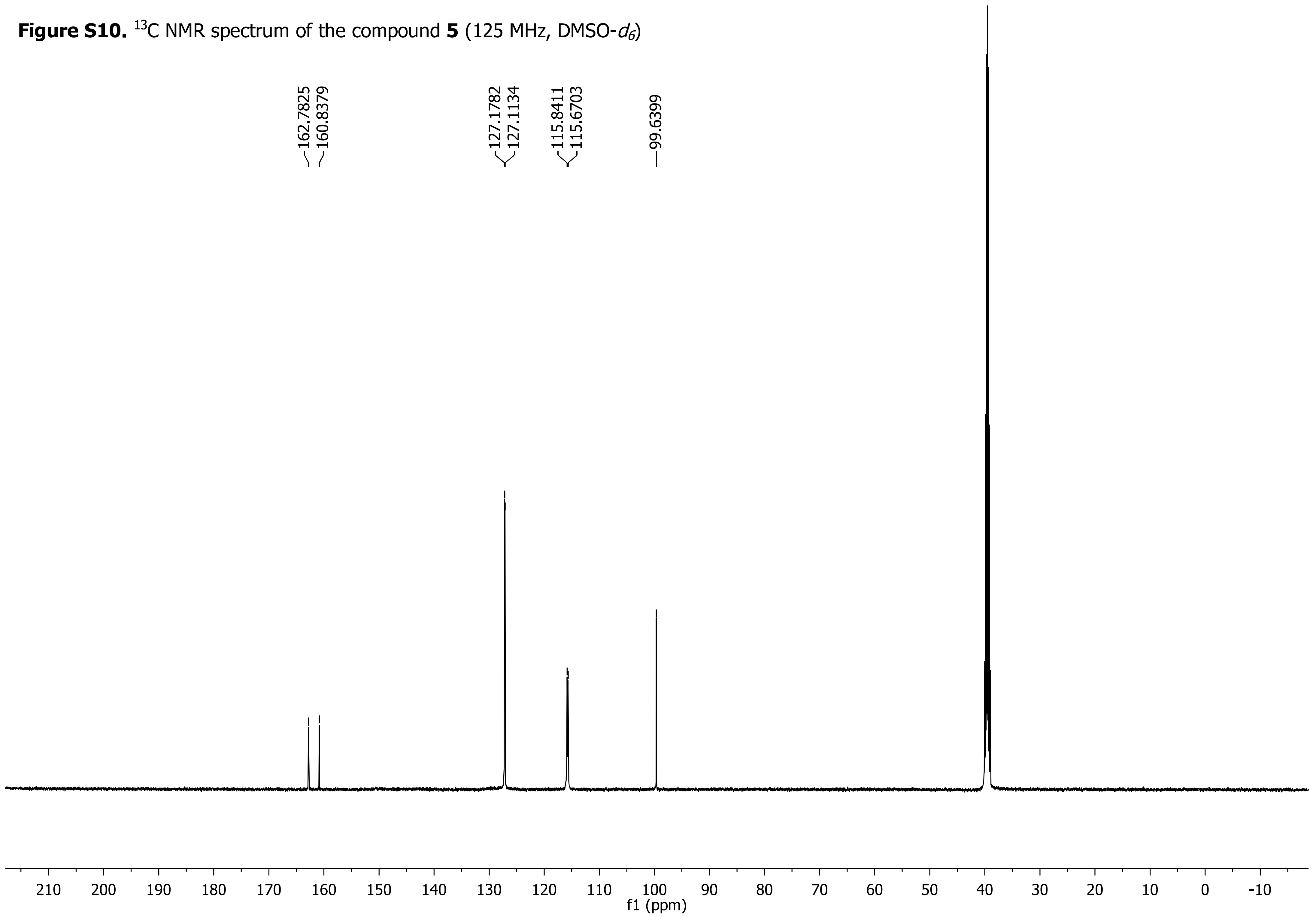

Figure S11. ^{1}H NMR spectrum of the compound **6** (500 MHz, DMSO-d_6)

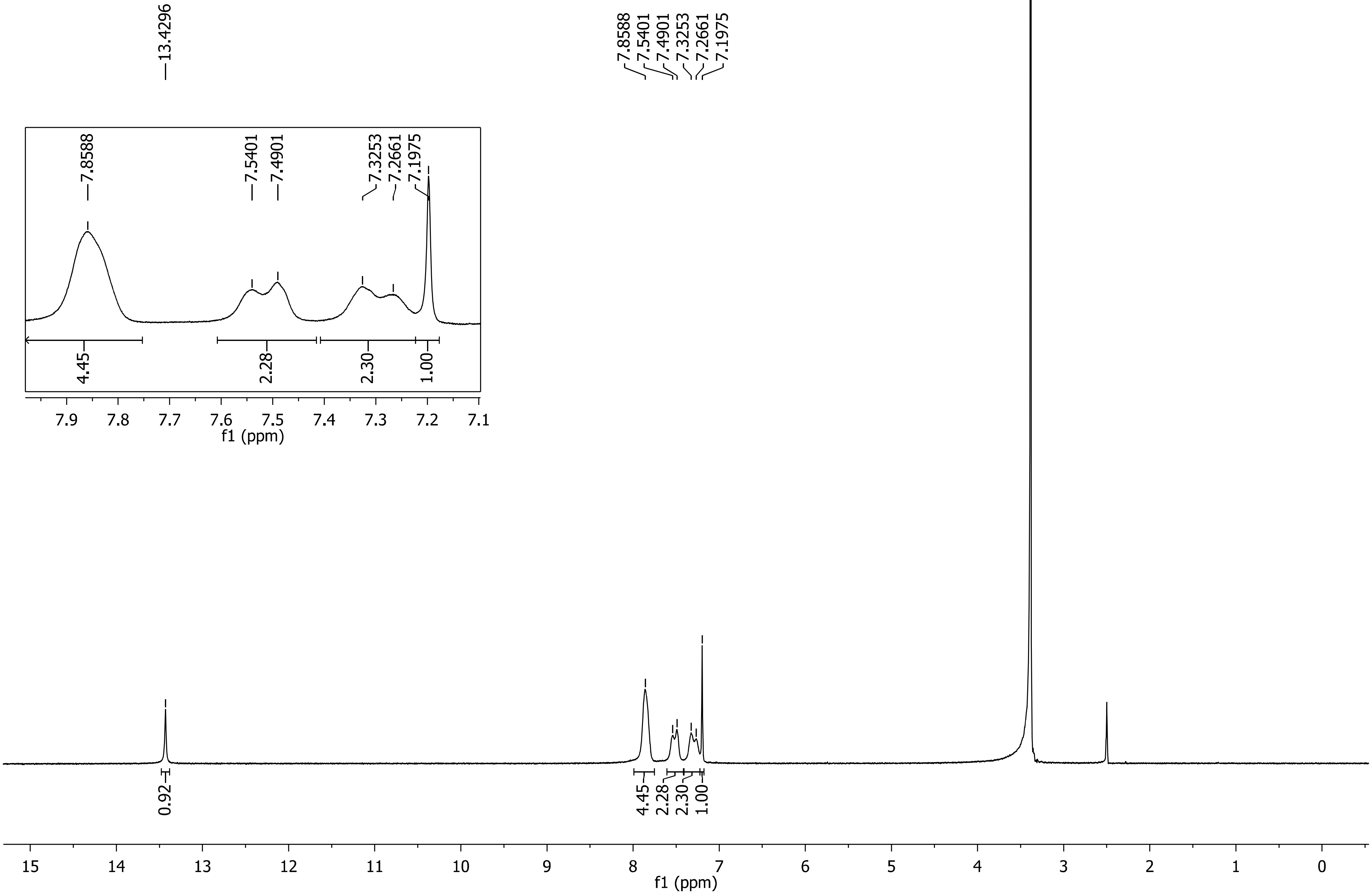

Figure S12. ^{13}C NMR spectrum of the compound **6** (125 MHz, DMSO-d_6)

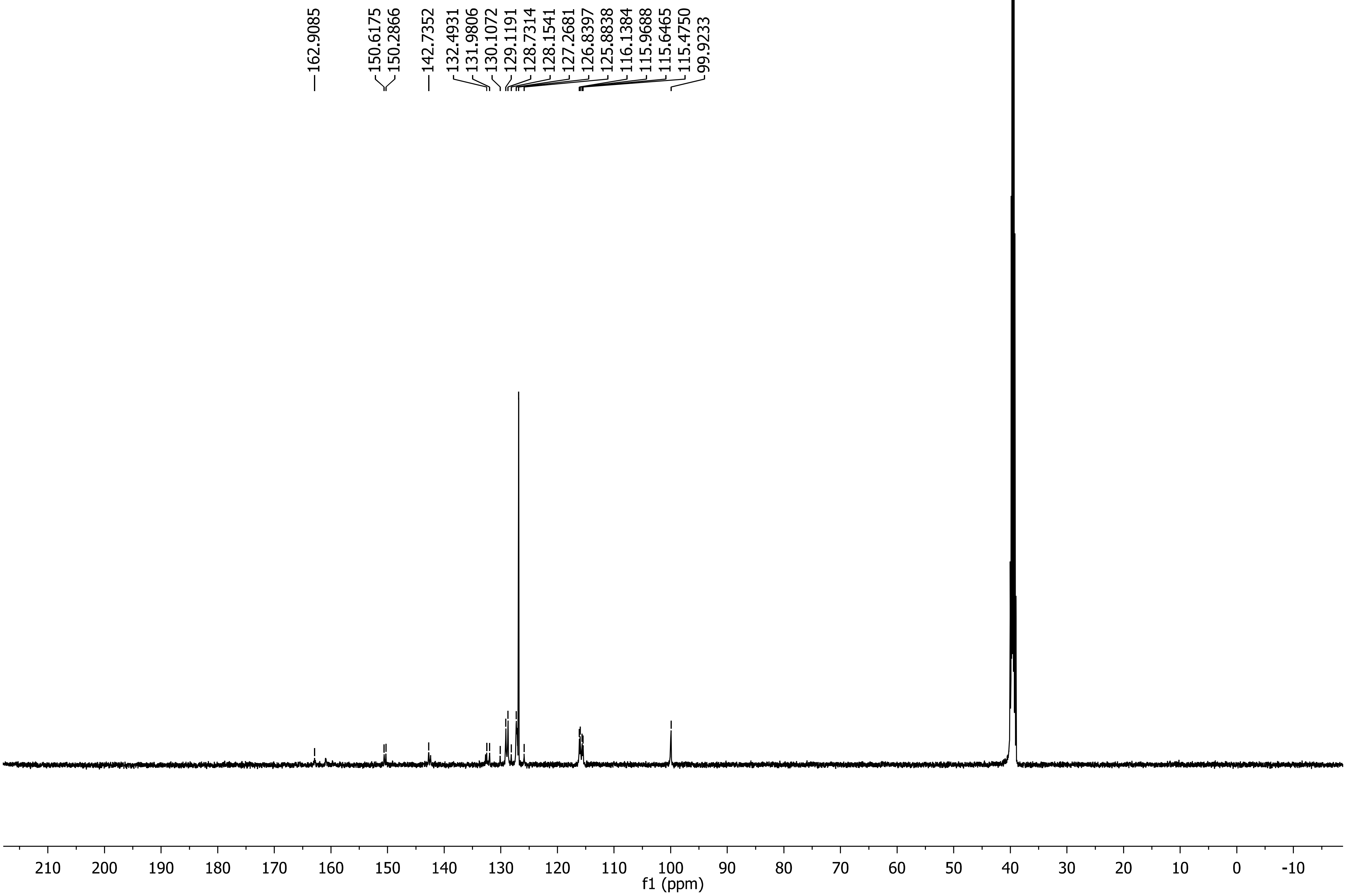

Figure S13. ^{1}H NMR spectrum of the compound **7** (500 MHz, DMSO-d_6)

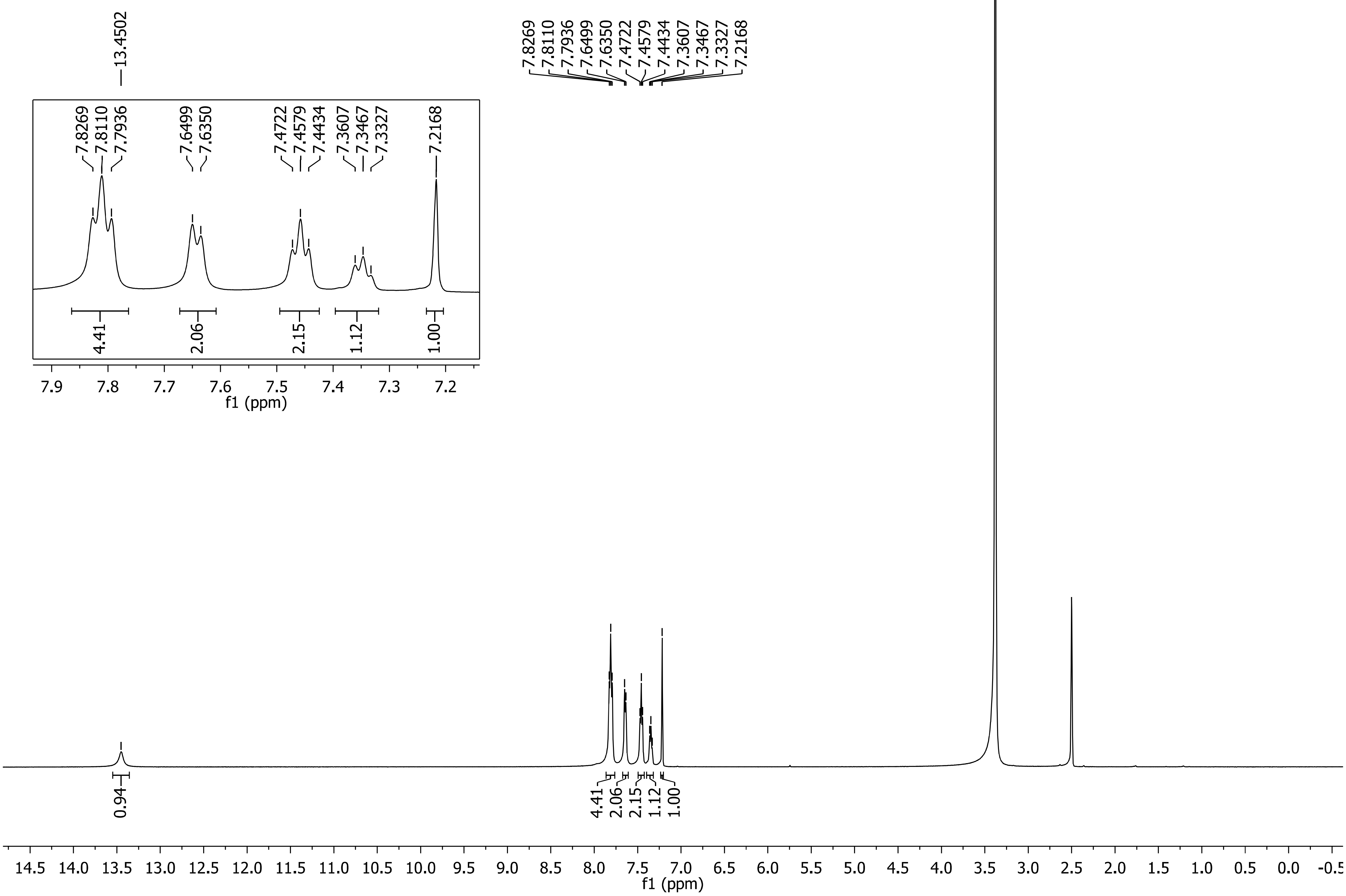

Figure S14. ^{13}C NMR spectrum of the compound **7** (125 MHz, DMSO-d_6)

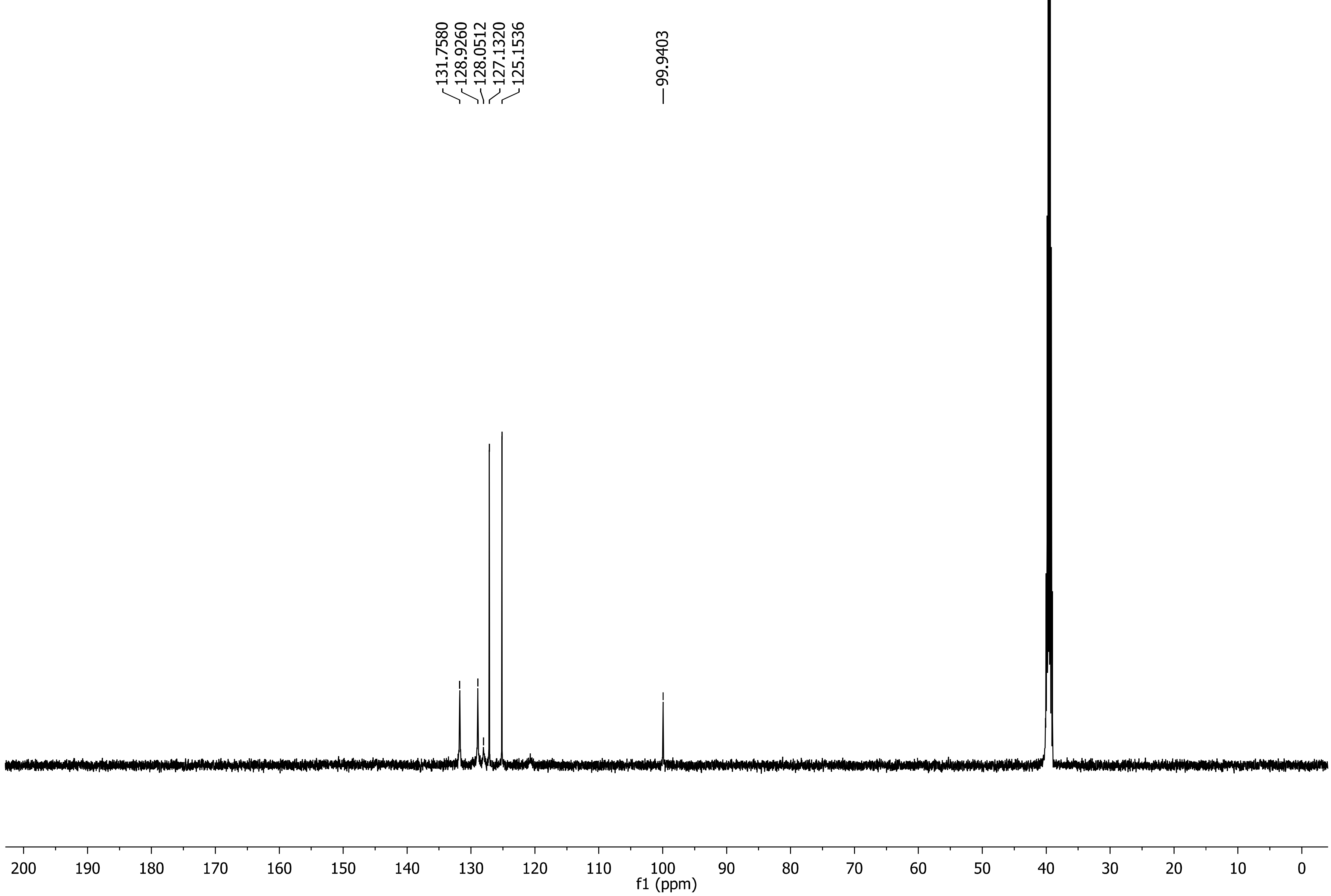

Figure S15. ^{1}H NMR spectrum of the compound **8** (500 MHz, DMSO-d_6)

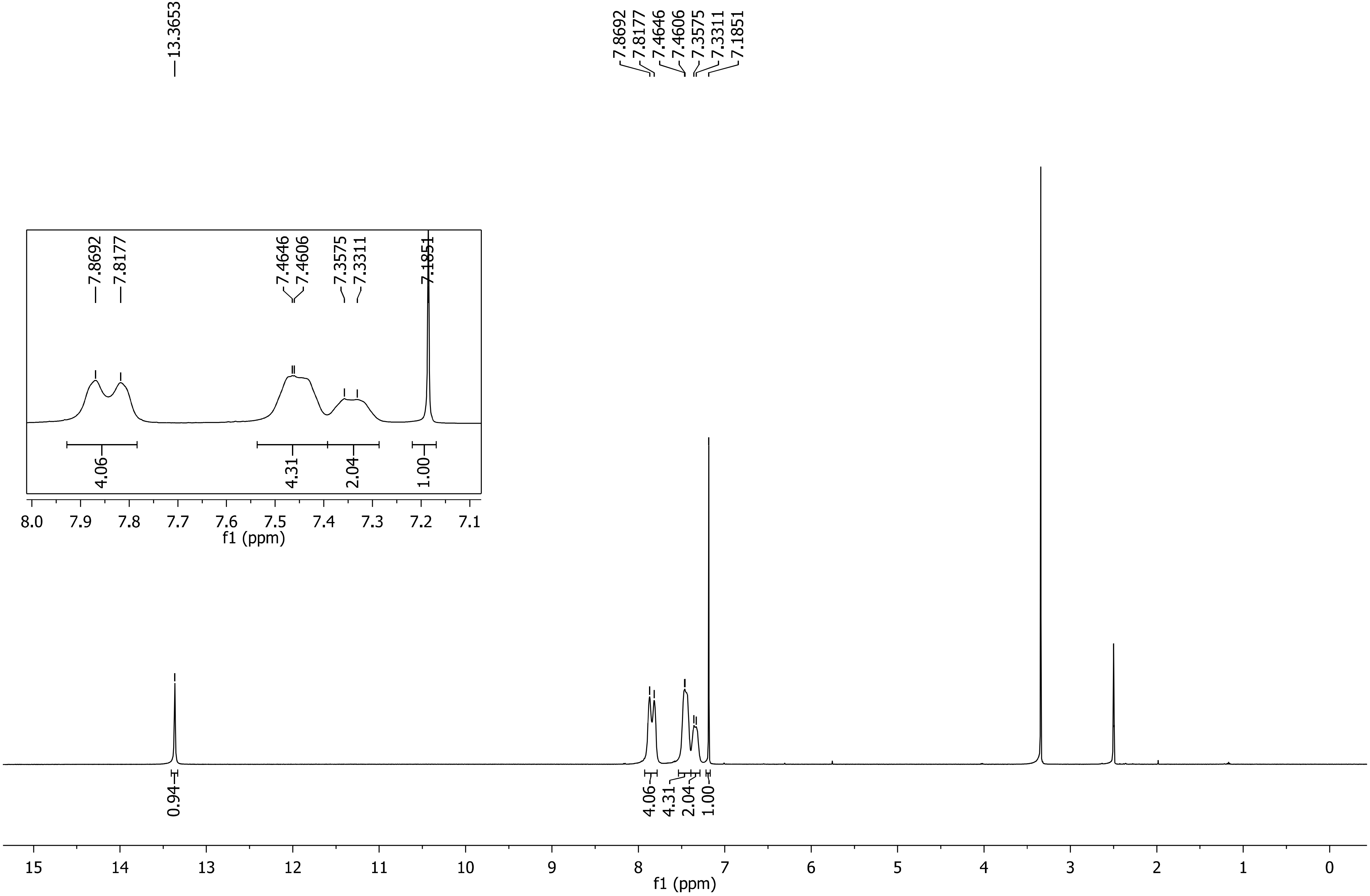

Figure S16. ^{13}C NMR spectrum of the compound **8** (125 MHz, DMSO-d_6)

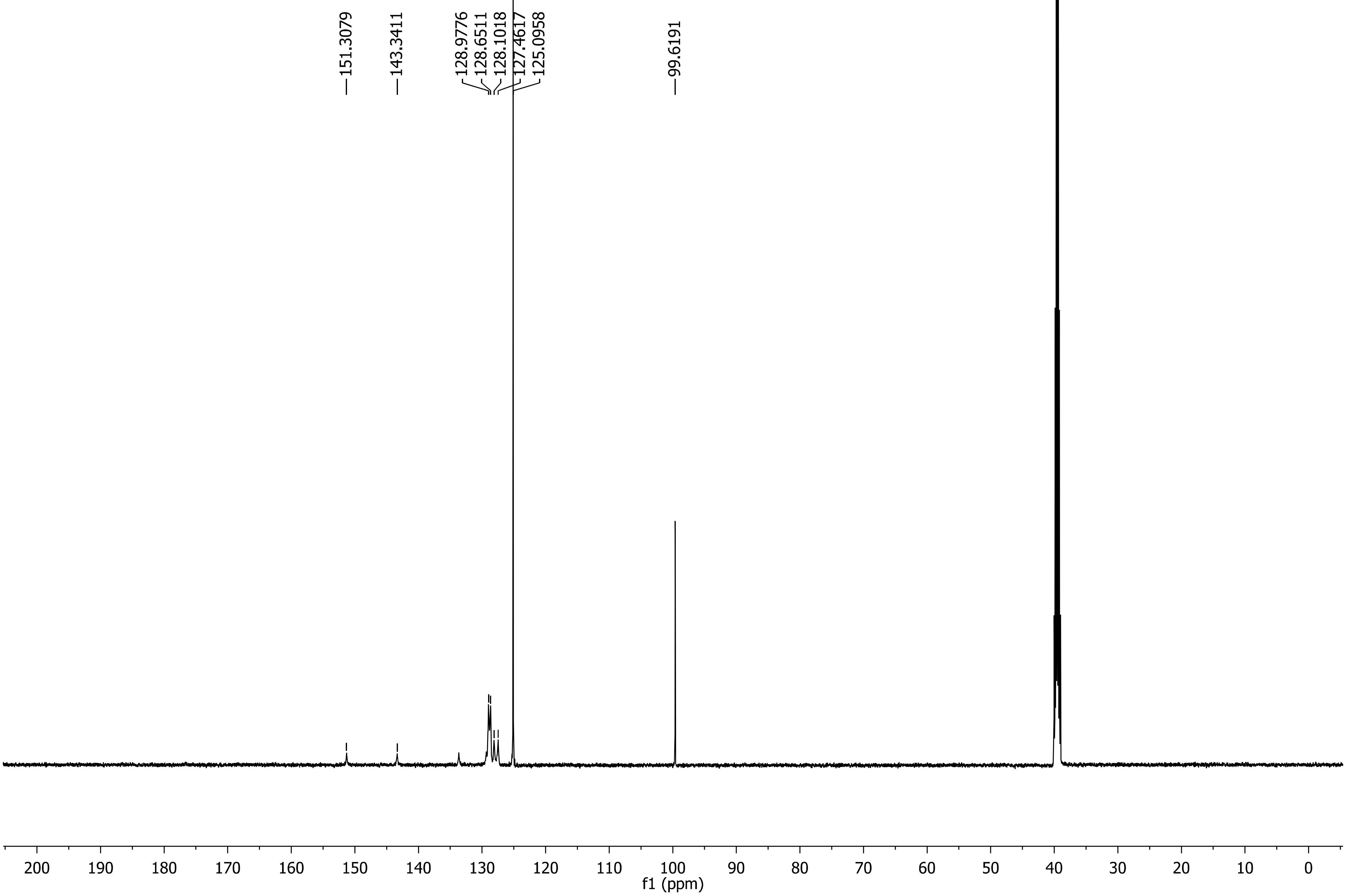

Figure S17. ^{1}H NMR spectrum of the compound **9** (500 MHz, DMSO-d_6)

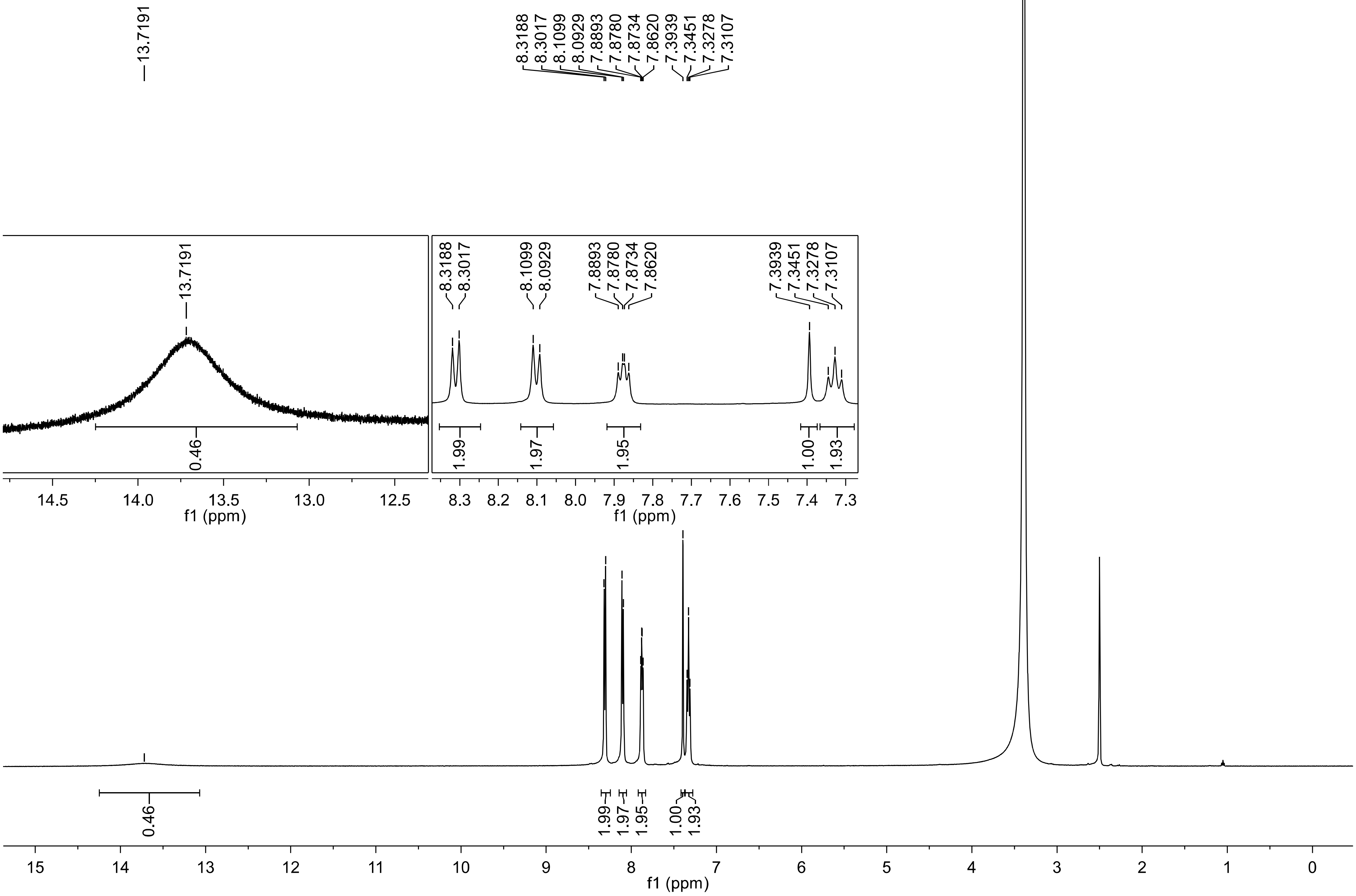

Figure S18. ^{13}C NMR spectrum of the compound **9** (125 MHz, DMSO-d_6)

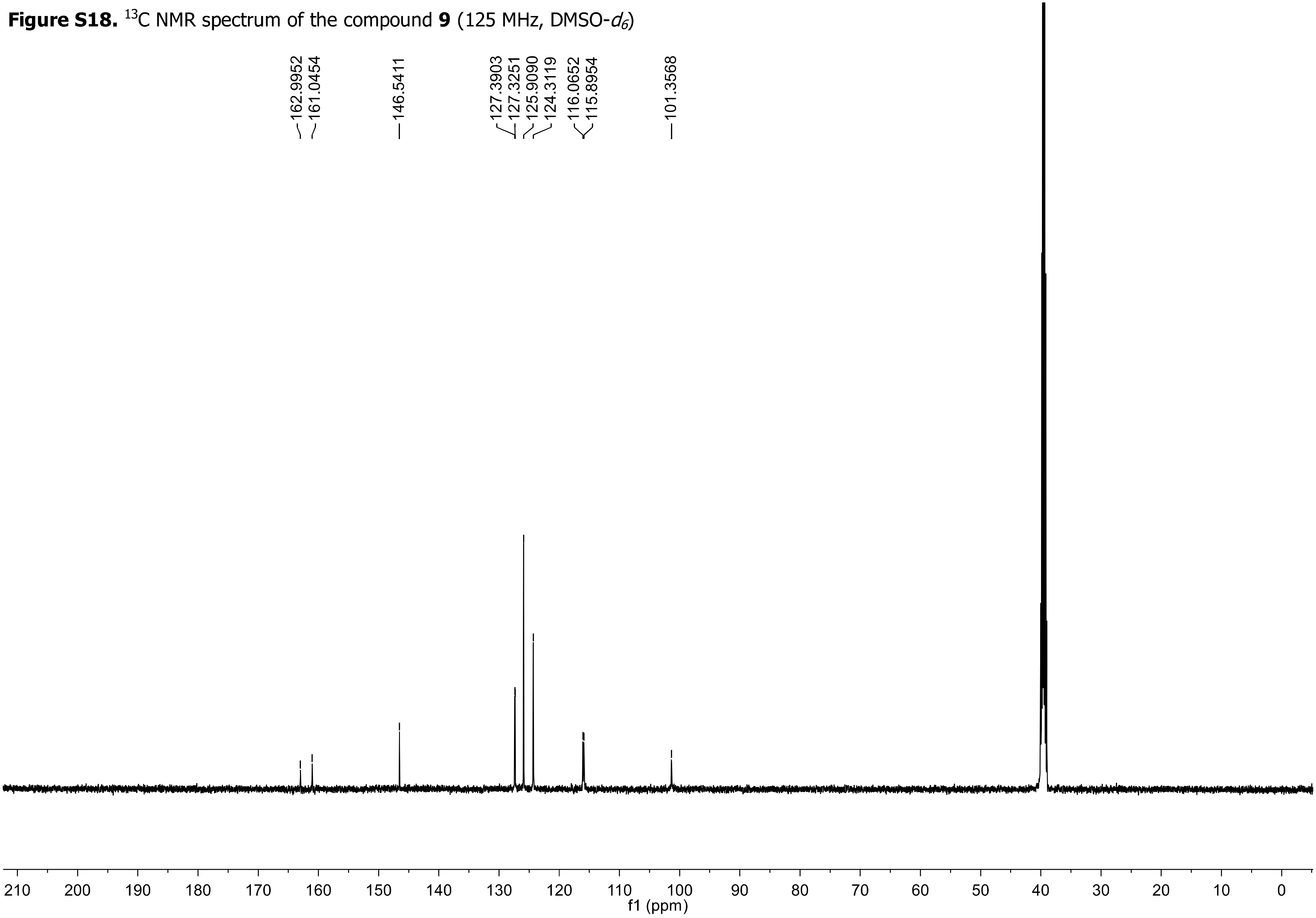

Figure S19. ^{1}H NMR spectrum of the compound **10** (500 MHz, DMSO-d_6)

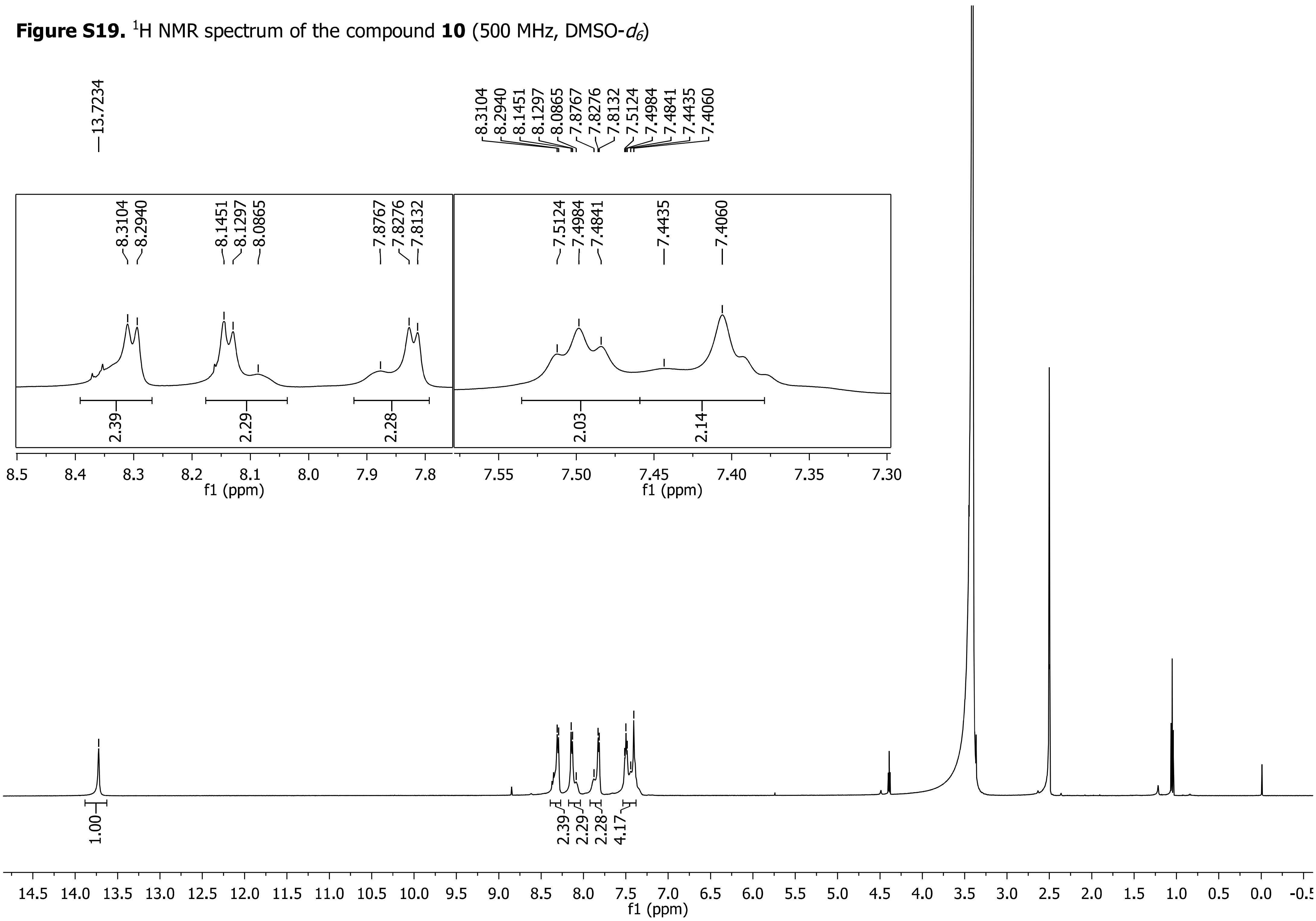

Figure S20. ^{13}C NMR spectrum of the compound **10** (125 MHz, DMSO-d_6)

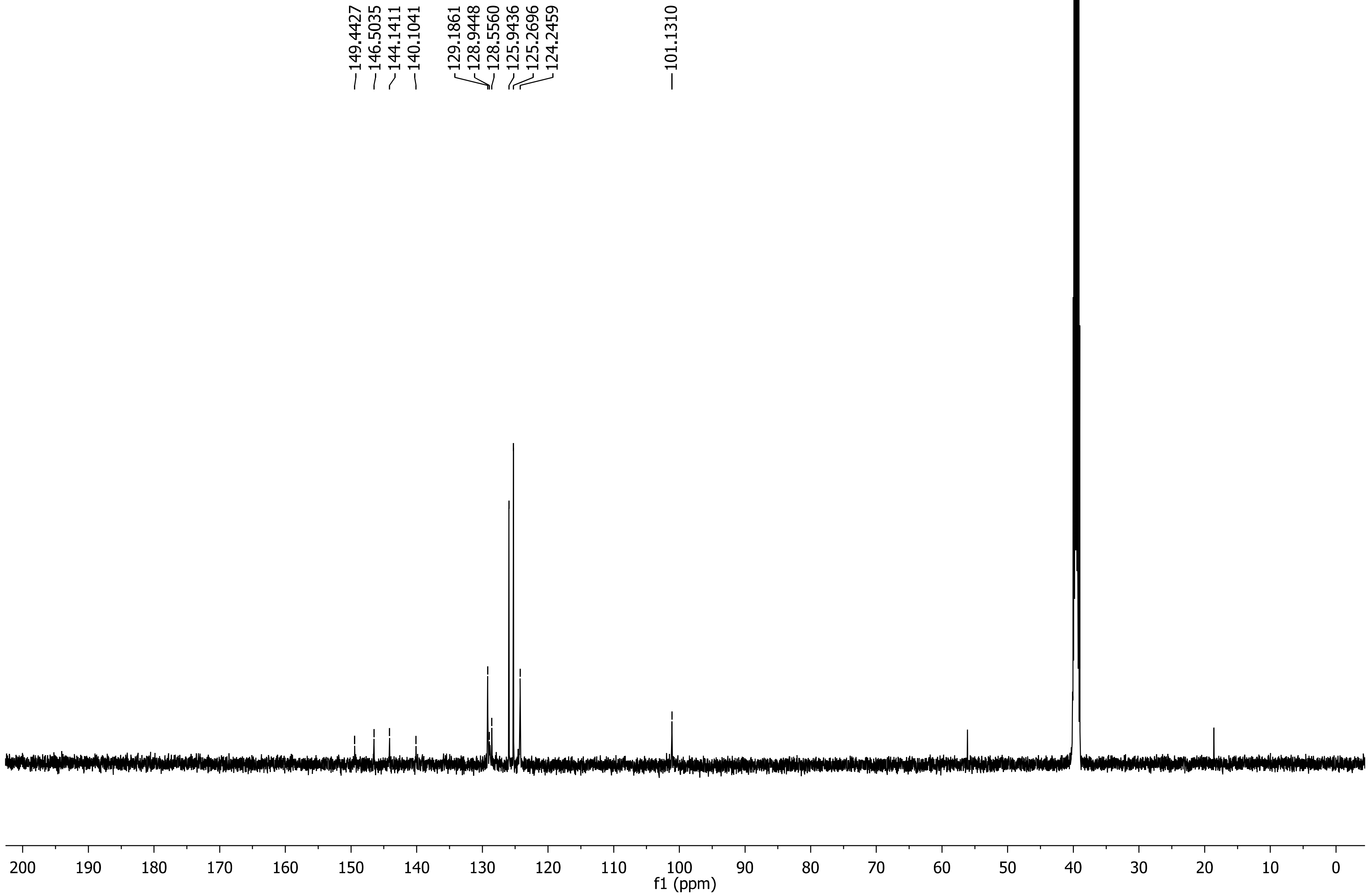

Figure S21. ^{1}H NMR spectrum of the compound **11** (500 MHz, DMSO-d_6)

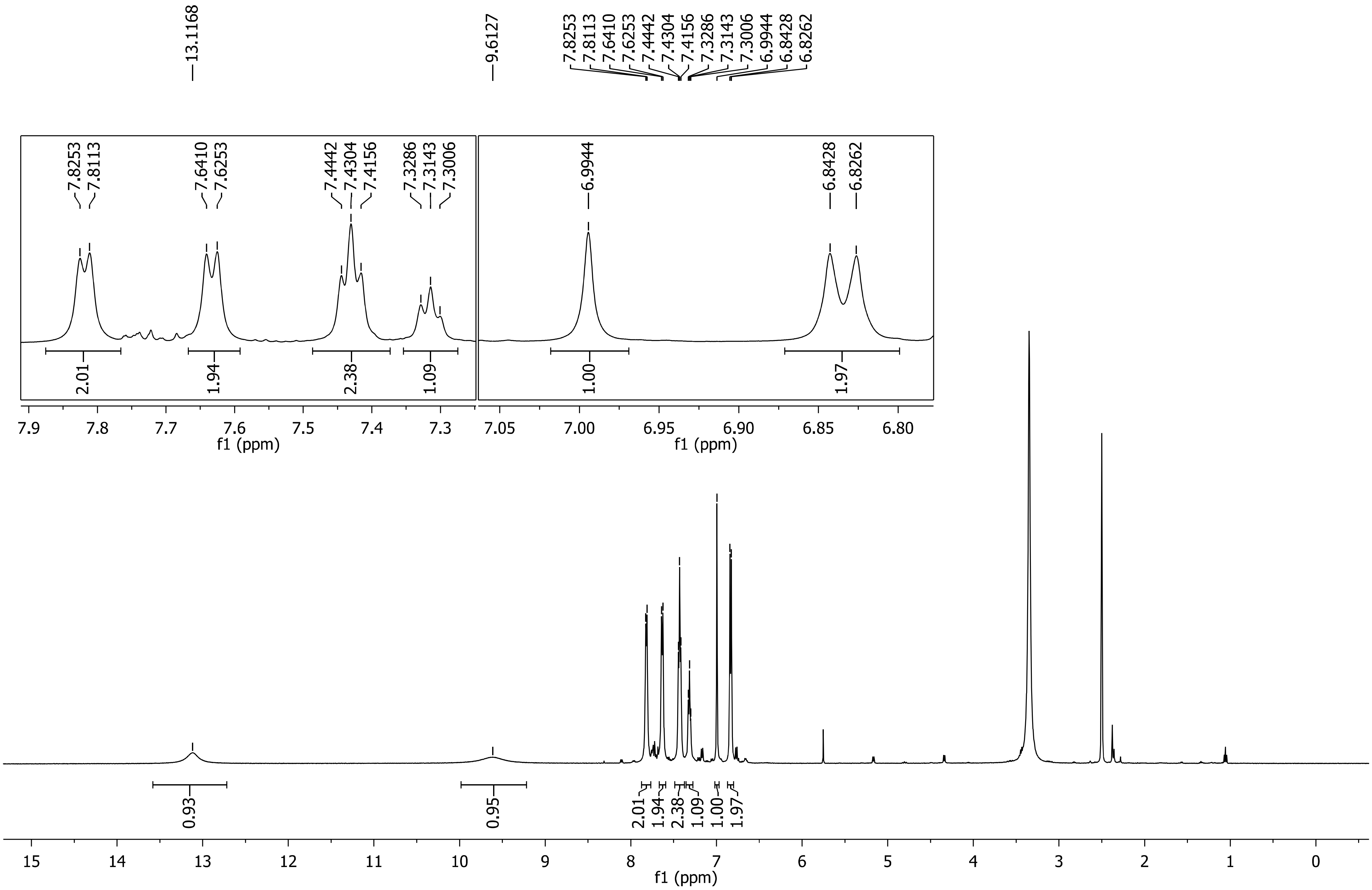

Figure S22. ^{13}C NMR spectrum of the compound **11** (125 MHz, DMSO-d_6)

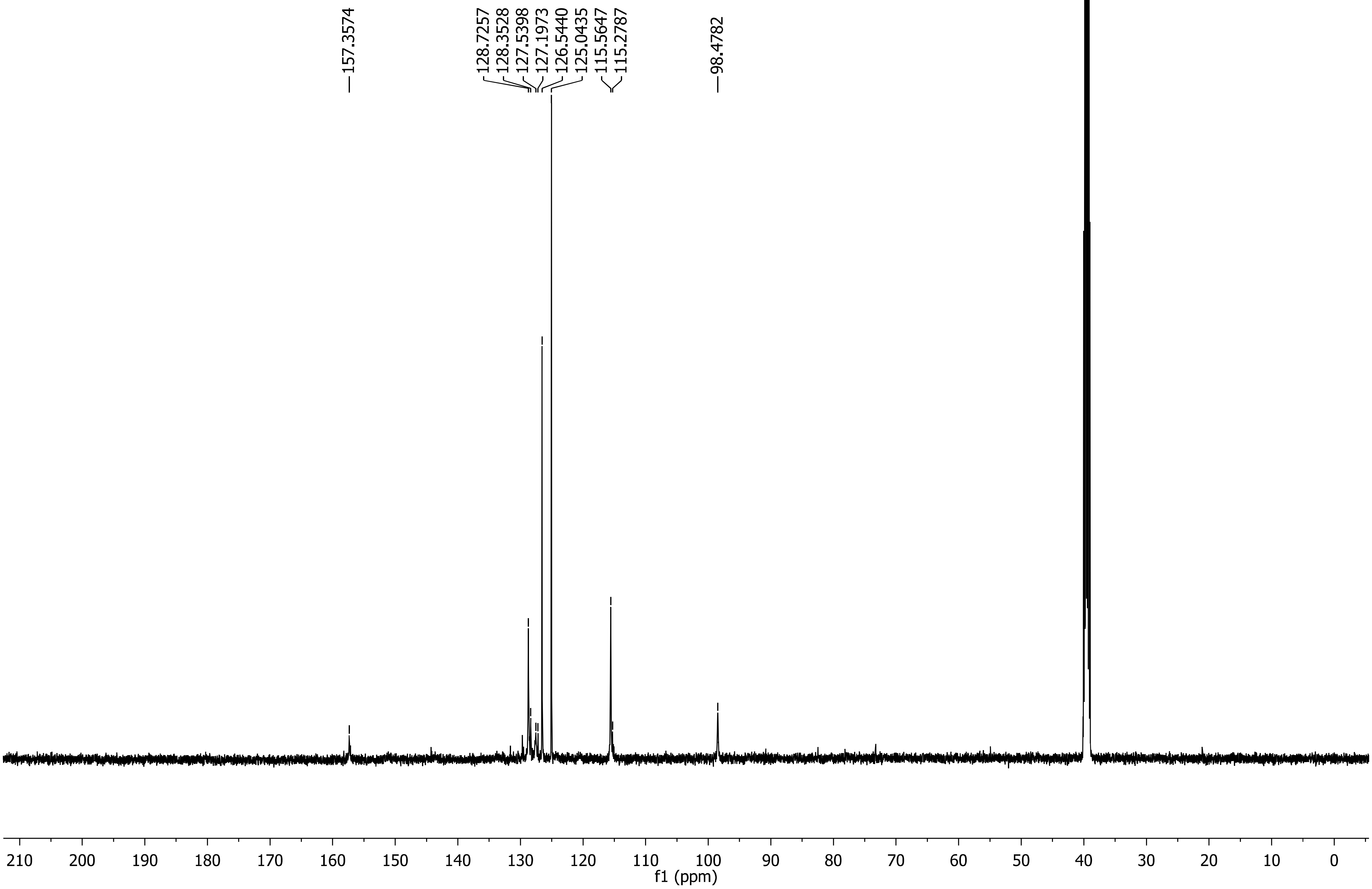

Figure S23. ^{1}H NMR spectrum of the compound **12** (500 MHz, DMSO-d_6)

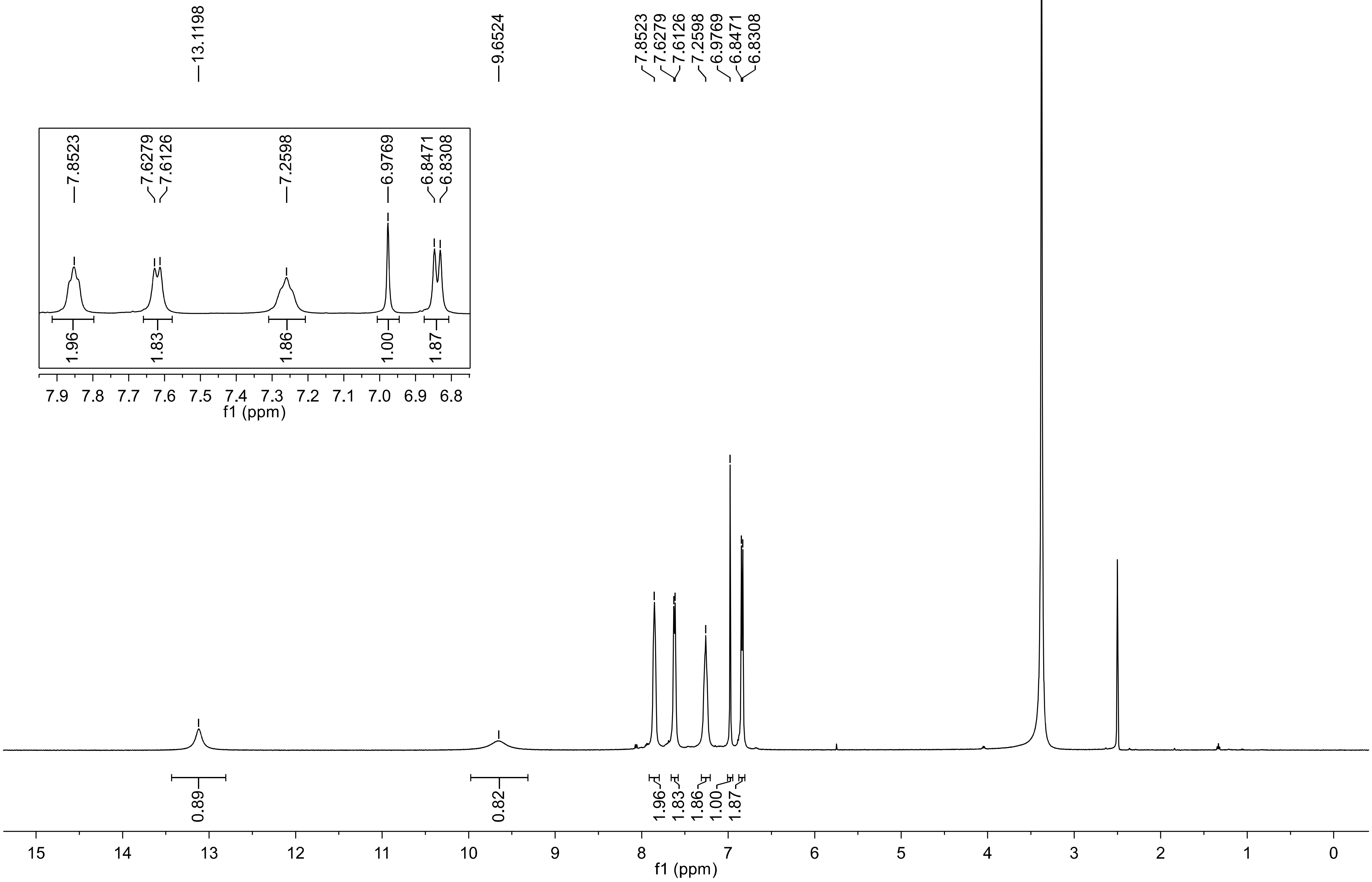

Figure S24. ^{13}C NMR spectrum of the compound **12** (125 MHz, DMSO-d_6)

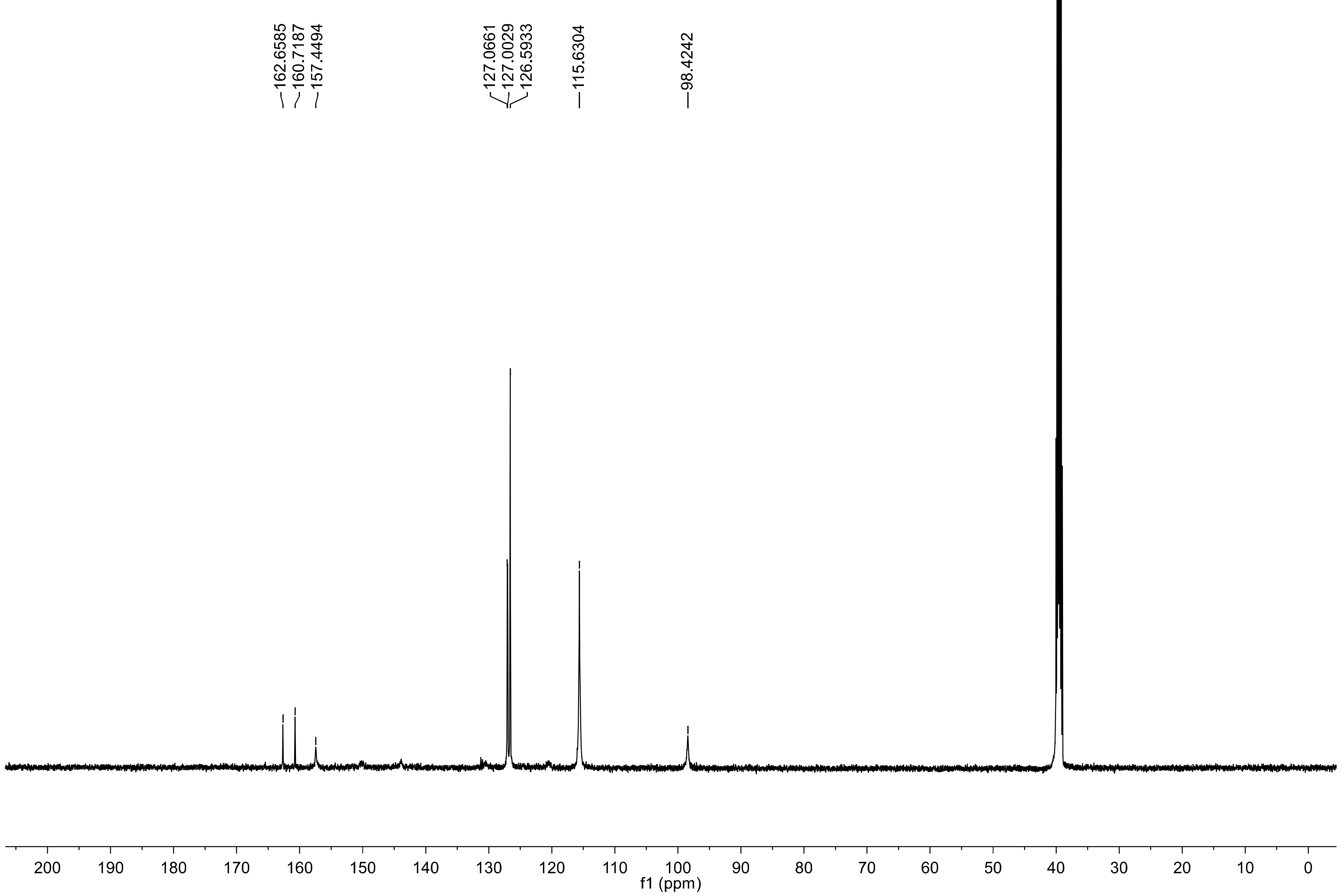

Figure S25. ^{1}H NMR spectrum of the compound **13** (500 MHz, DMSO-d_6)

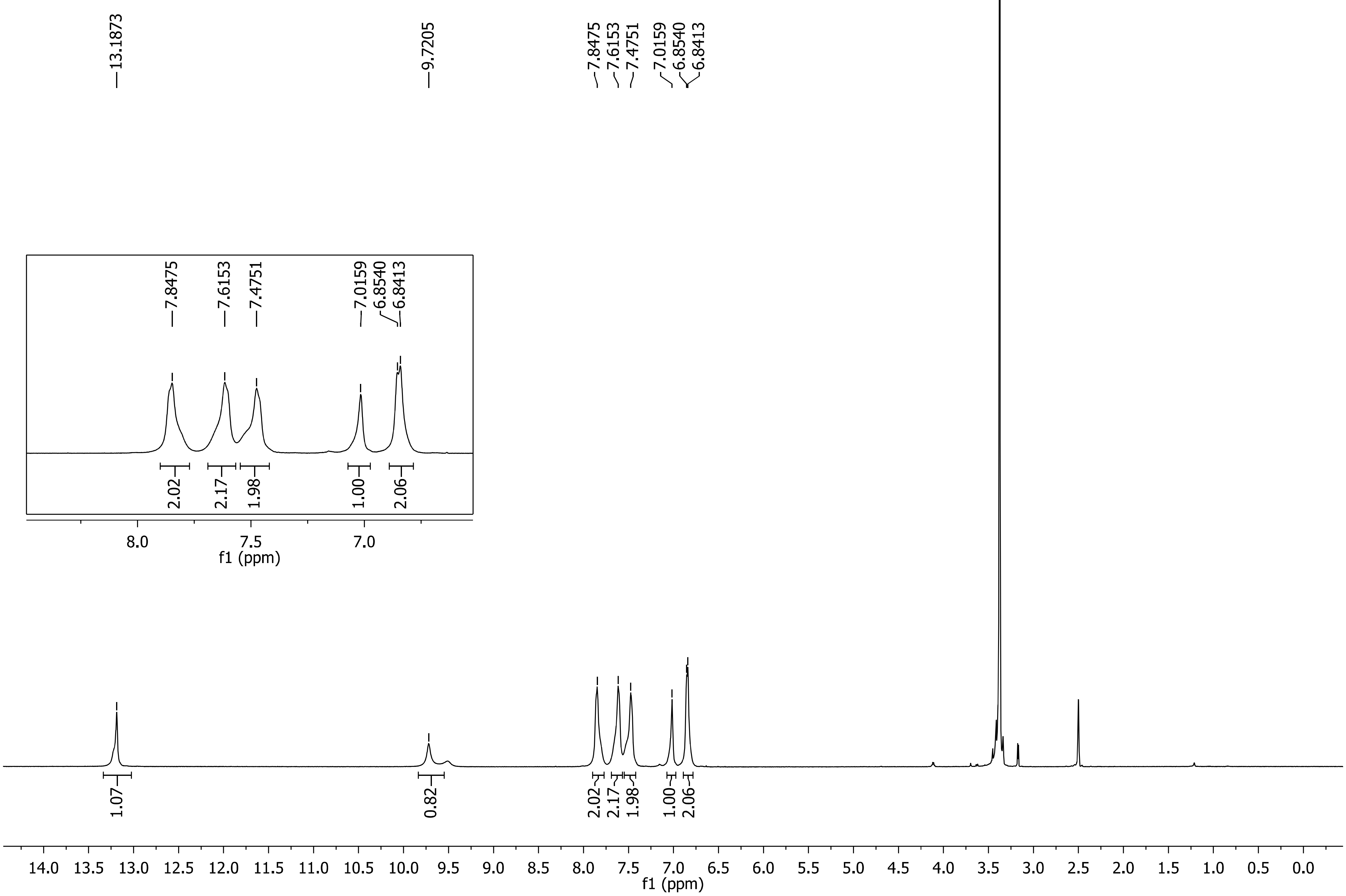

Figure S26. ^{13}C NMR spectrum of the compound **13** (125 MHz, DMSO-d_6).

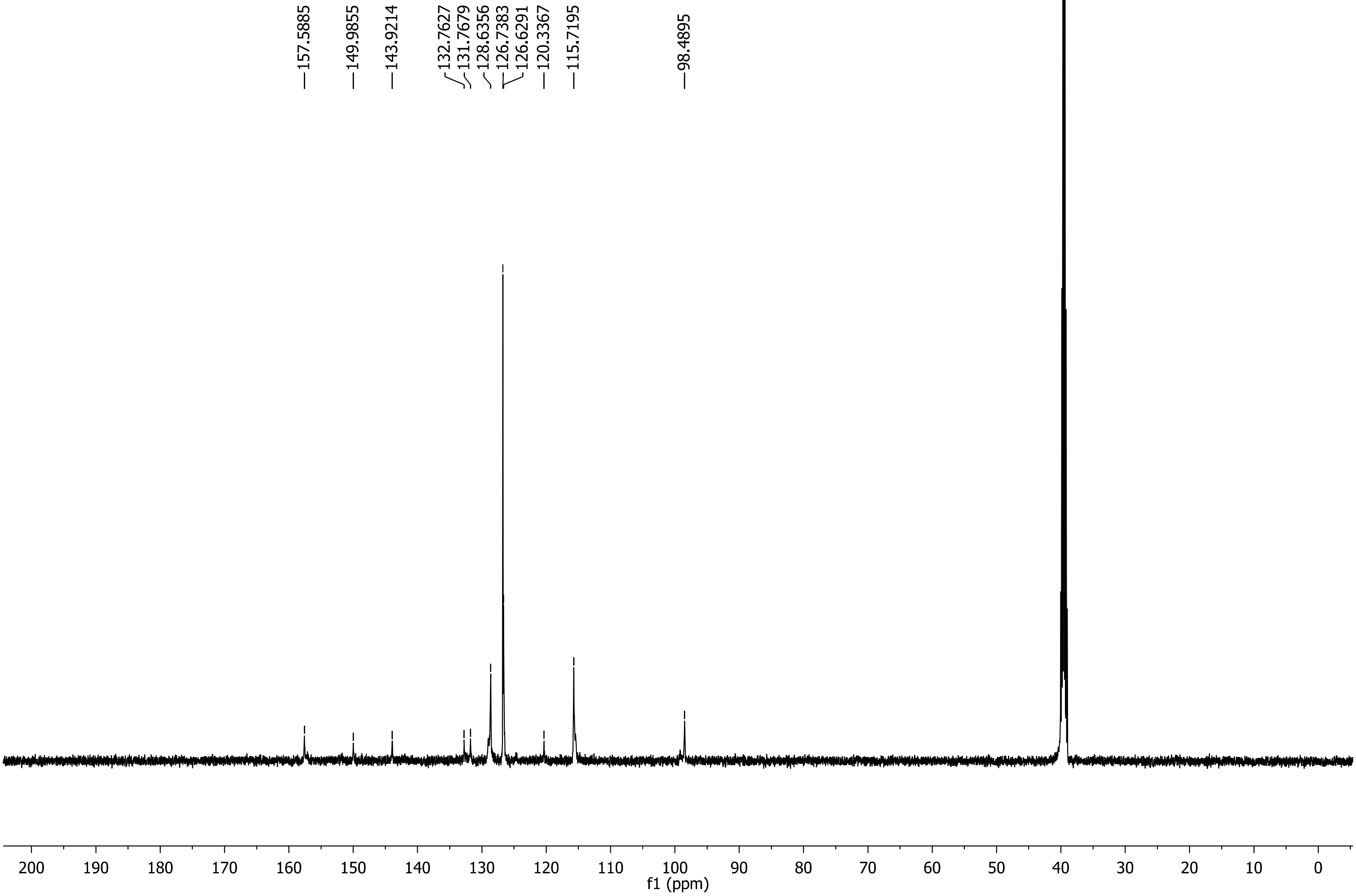

2. XRD

Table S1. Summary of the Crystal Data and Refinement Details for **9** and **13**.

	**9**	**13**
Formula	$C_{15}H_{10}F_1N_3$	$(C_{15}H_{11}ClN_2O), C_2H_6O$
Formula weight	283.26	293.74
Cryst. syst.	orthorhombic	monoclinic
Space group	P n a 21	C 2/c'
a / Å	10.8792(4)	33.14(3)
b / Å	25.5696(7)	14.749(13)
c / Å	4.64630(10)	6.248(6)
□/°	90.00	92.64(4)
V / $Å^3$	1292.49(7)	3050(5)
Z	4	4
Reflns. Collected	17038	52379
Independent reflns.	2307	2638
reflns. with [I > 2□(I)]	2136	1530
Rint	0.0539	0.0899
No. of parameters	190	200
GOF on F^2	1.087	1.166
R1 [I > 2□(I)]	0.0539	0.0592
wR2 (all data)	0.1547	0.2042
Largest diff. peak and hole (e $Å^{-3}$)	0.161/ -0.220	0.363/-0.355
CCDC deposit	2333182	2333183

3. ESI-HRMS

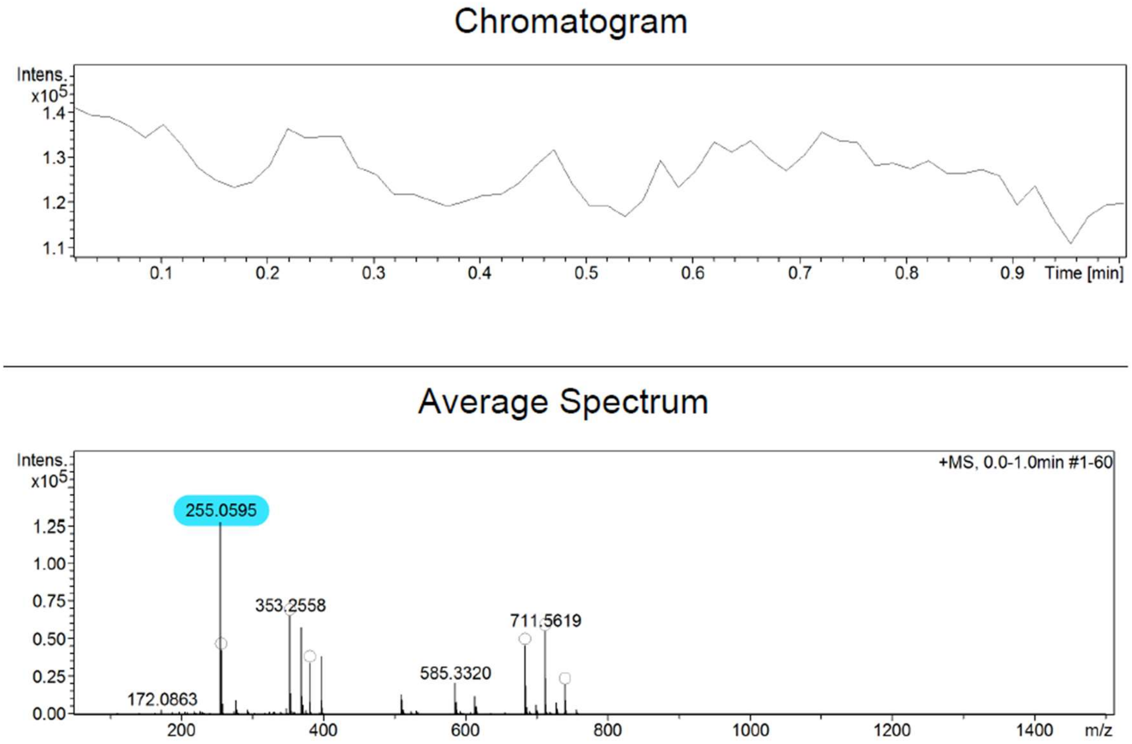

Figure S27. ESI-HRMS of **12**.

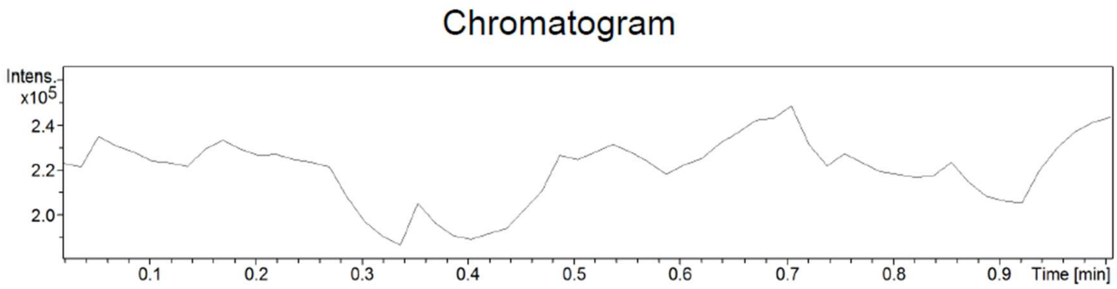

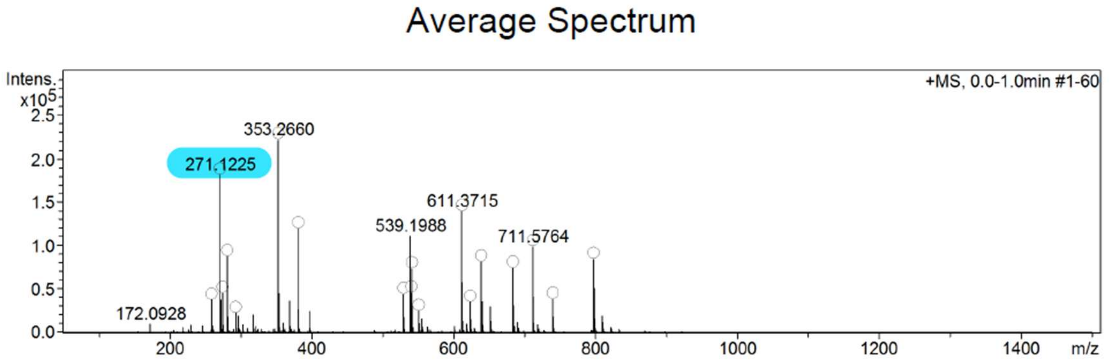

Figure S28. ESI-HRMS of **13**.

4. Experimental planning

Table S2. 3^3 Box-Behnken design regression model fitted for conversion (%).

Factors	Regression Coefficients	Standard error	t(2)	*p*-value
b_0	60.53	0.67	89.47	0.000125
b_1	17.97	0.41	43.38	0.000531
b_2	5.77	0.41	13.93	0.005108
b_3	16.45	0.41	39.70	0.000634
b_{11}	-6.75	0.61	-11.07	0.008055
b_{22}	-3.35	0.61	-5.50	0.031506
b_{33}	-9.15	0.61	-15.01	0.004409
b_{12}	-0.97	0.58	-1.66	0.238023
b_{13}	4.62	0.58	7.89	0.015674
b_{23}	3.47	0.58	5.93	0.027274

Sum of squares due to lack of fit: 12.75; sum of squares due to pure error: 1.37; freedom degrees: 2.

4. Cycloaddition reactions

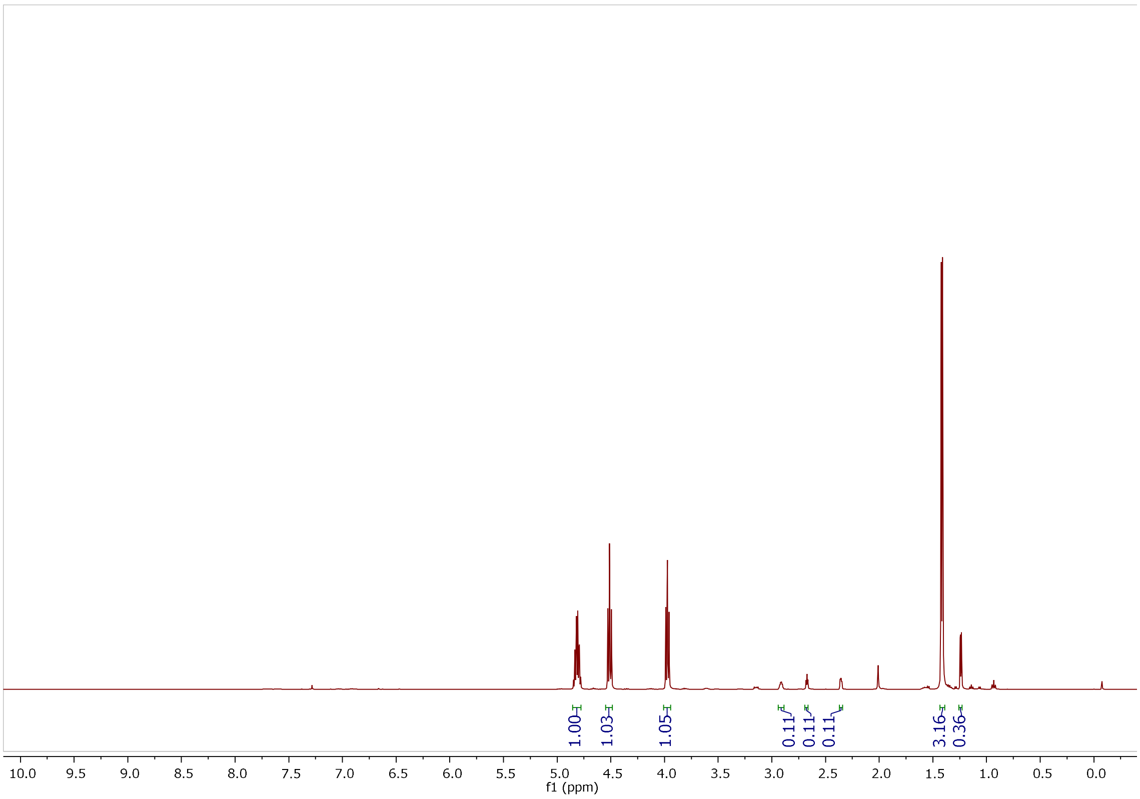

Figure S29. ^1H NMR spectrum of the reaction mixture using catalyst **13** and **PO** (Entry 8, Table 1) (500 MHz, $CDCl_3$).

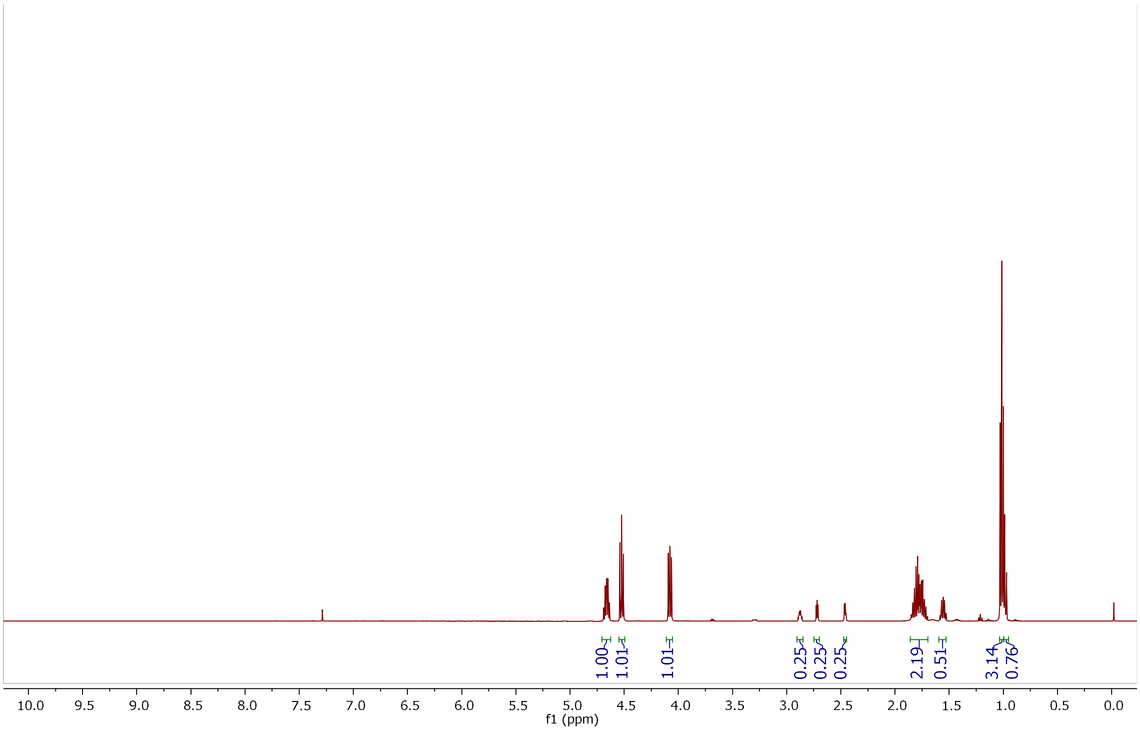

Figure S30. ^{1}H NMR spectrum of the reaction mixture using catalyst **13** and **EB** (Figure 4) (500 MHz, $CDCl_3$).

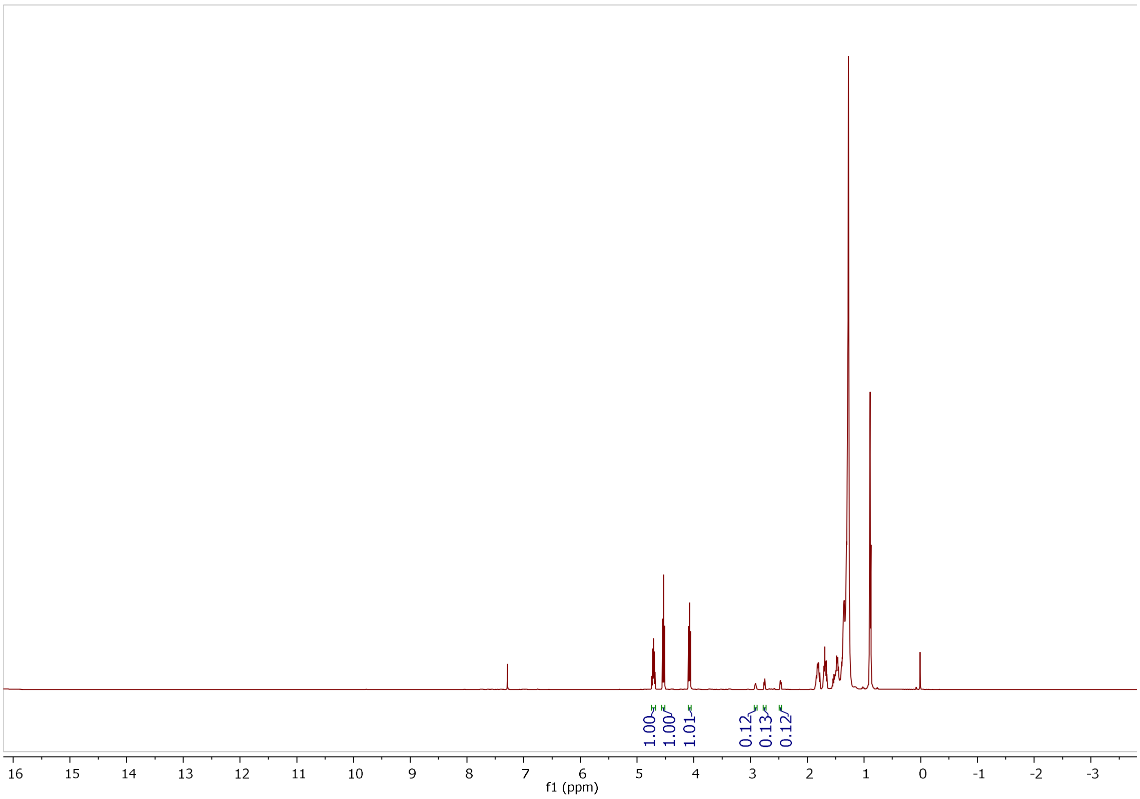

Figure S31. ^{1}H NMR spectrum of the reaction mixture using catalyst **13** and **ED** (Figure 4) (500 MHz, $CDCl_3$).

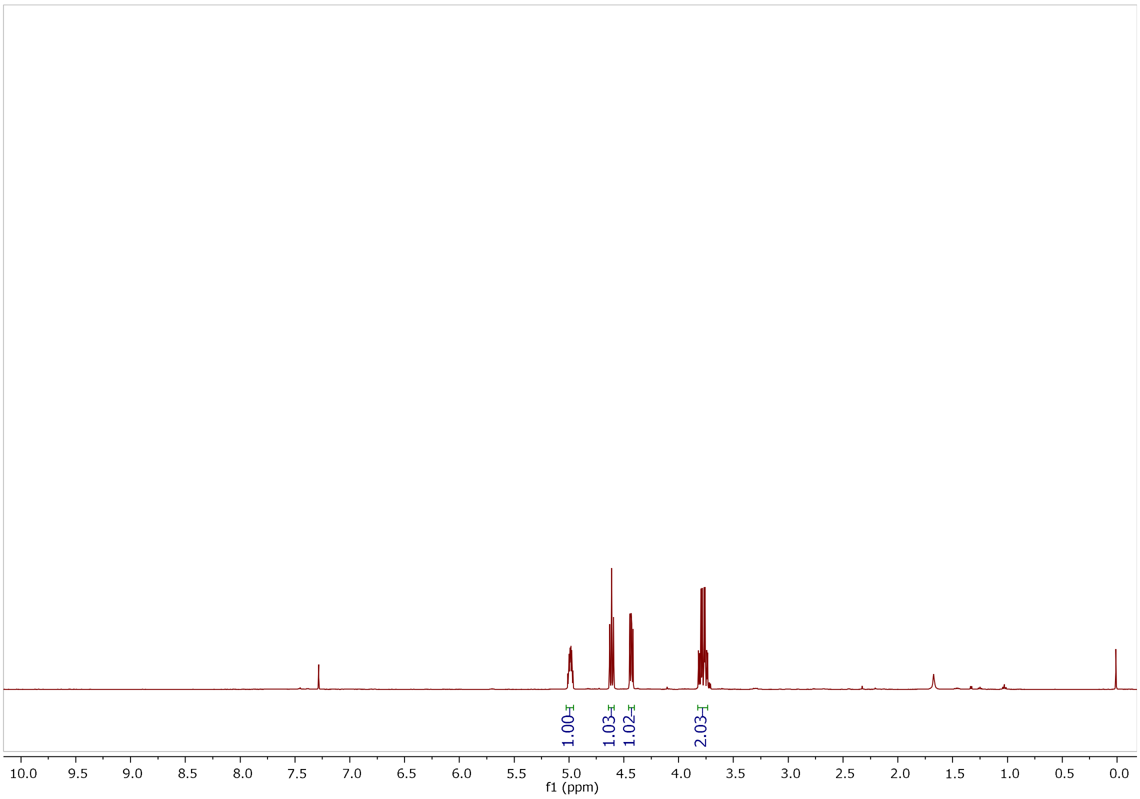

Figure S32. ^{1}H NMR spectrum of the reaction mixture using catalyst **13** and **ECH** (Figure 4) (500 MHz, $CDCl_3$).

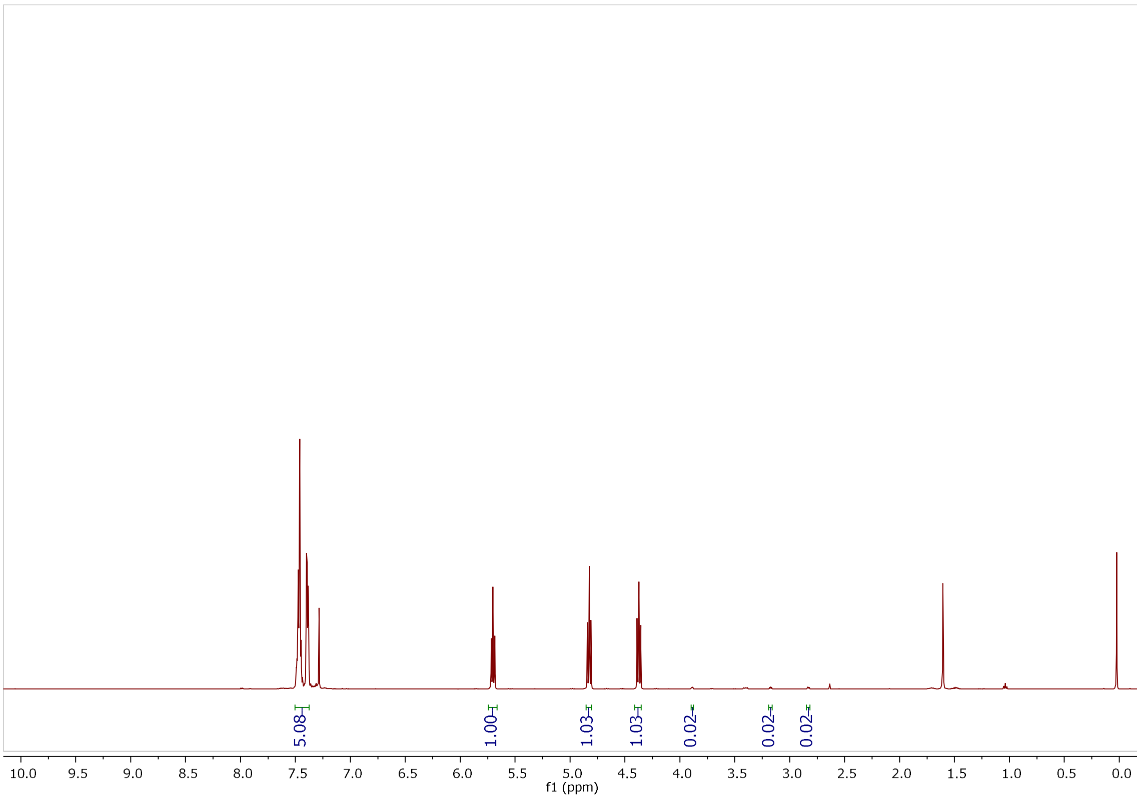

Figure S33. ^{1}H NMR spectrum of the reaction mixture using catalyst **13** and **SO** (Figure 4) (500 MHz, $CDCl_3$).

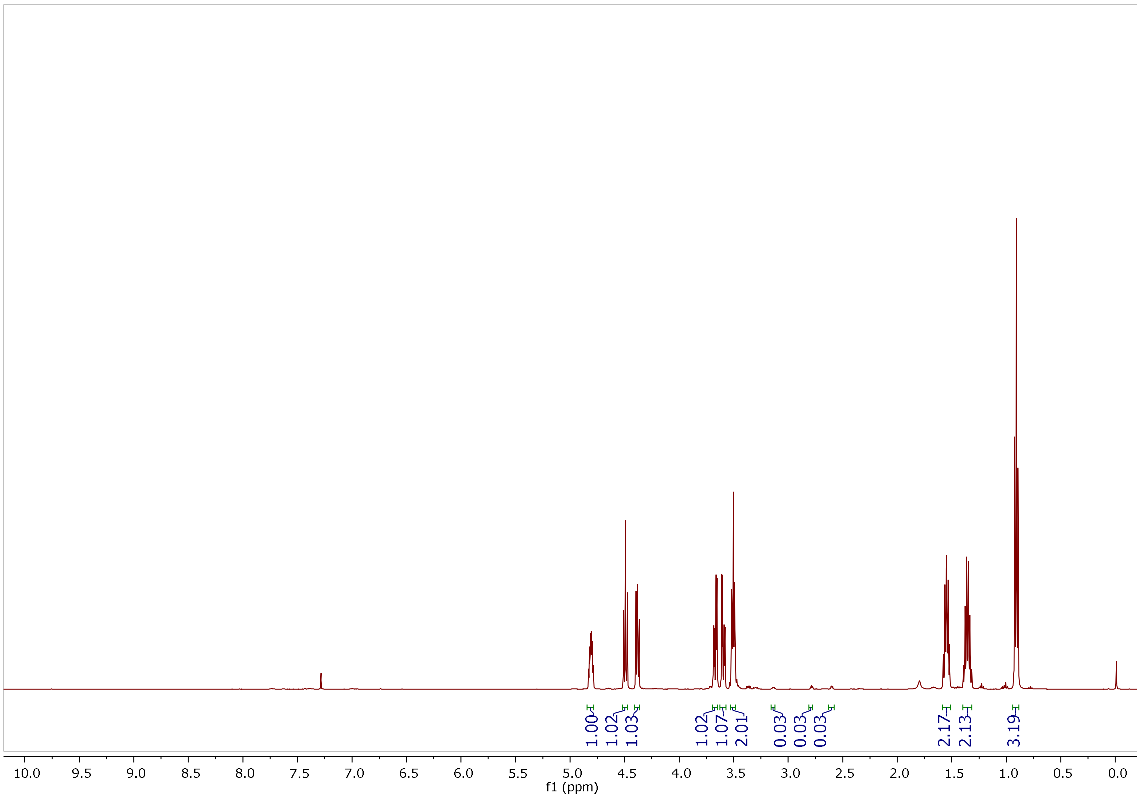

Figure S34. ^{1}H NMR spectrum of the reaction mixture using catalyst **13** and **BGE** (Figure 4) (500 MHz, $CDCl_3$).

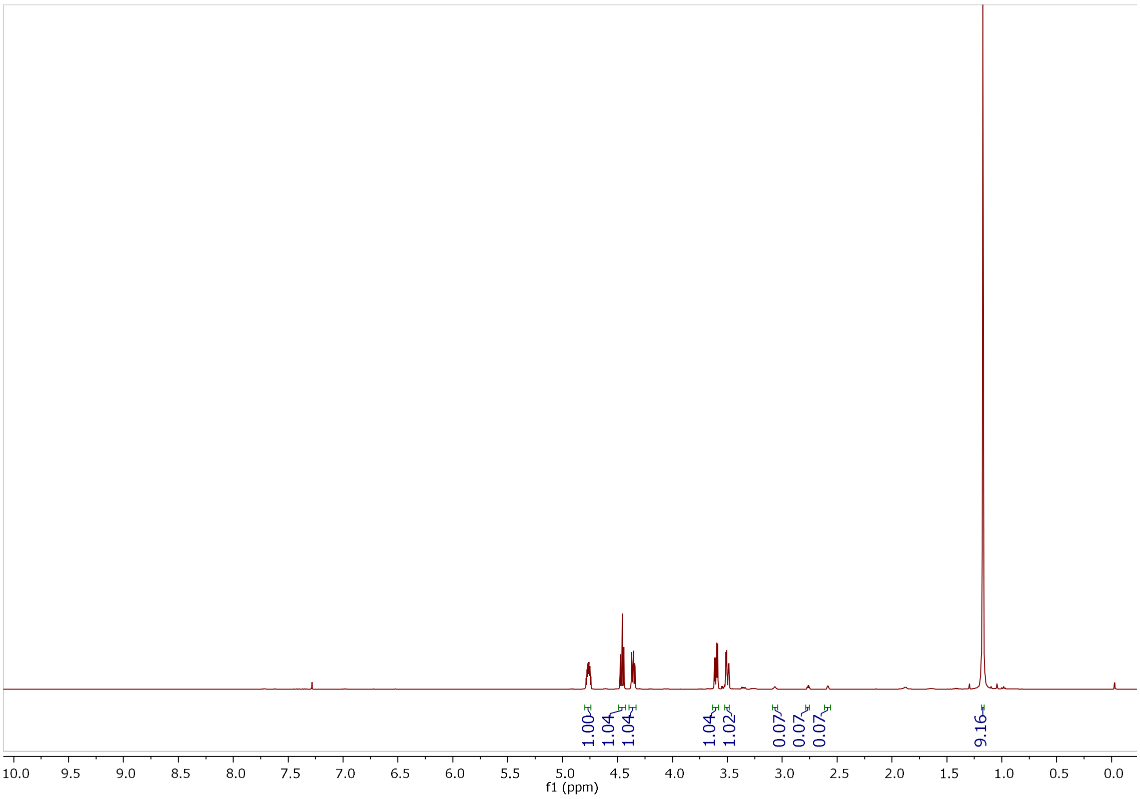

Figure S35. ^{1}H NMR spectrum of the reaction mixture using catalyst **13** and **TBGE** (Figure 4) (500 MHz, $CDCl_3$).

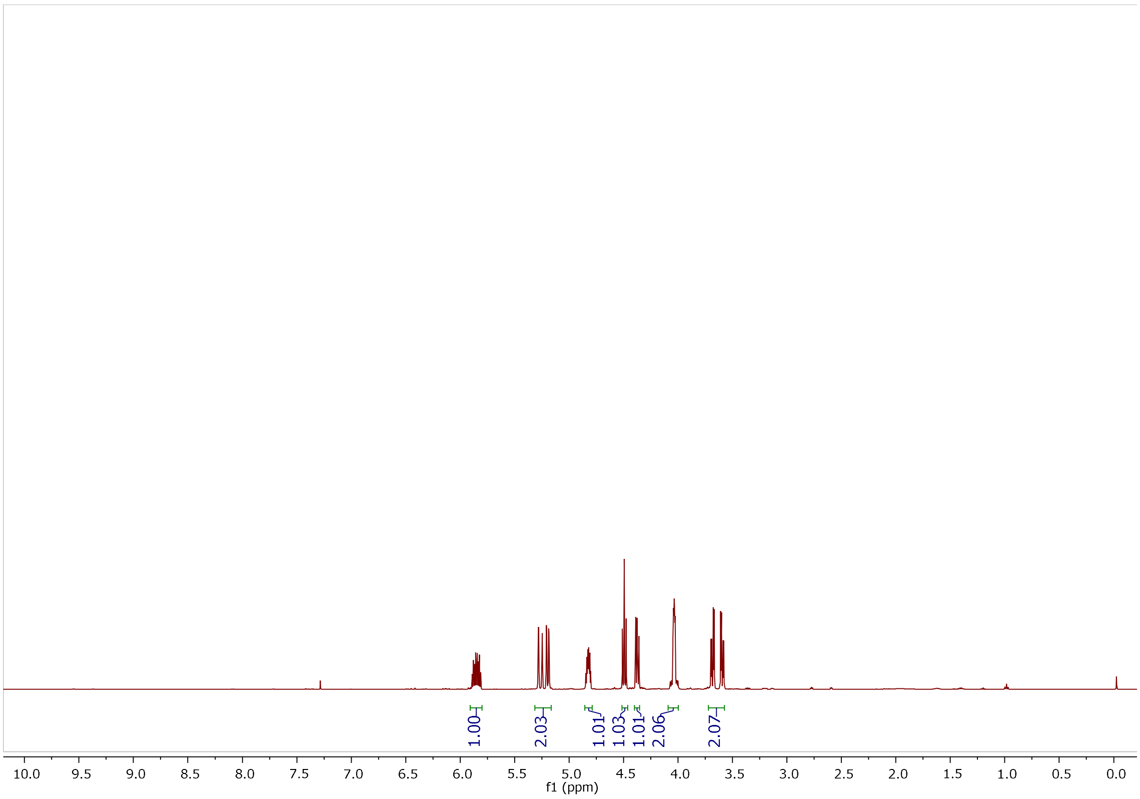

Figure S36. ^1H NMR spectrum of the reaction mixture using catalyst **13** and **AGE** (Figure 4) (500 MHz, $CDCl_3$).

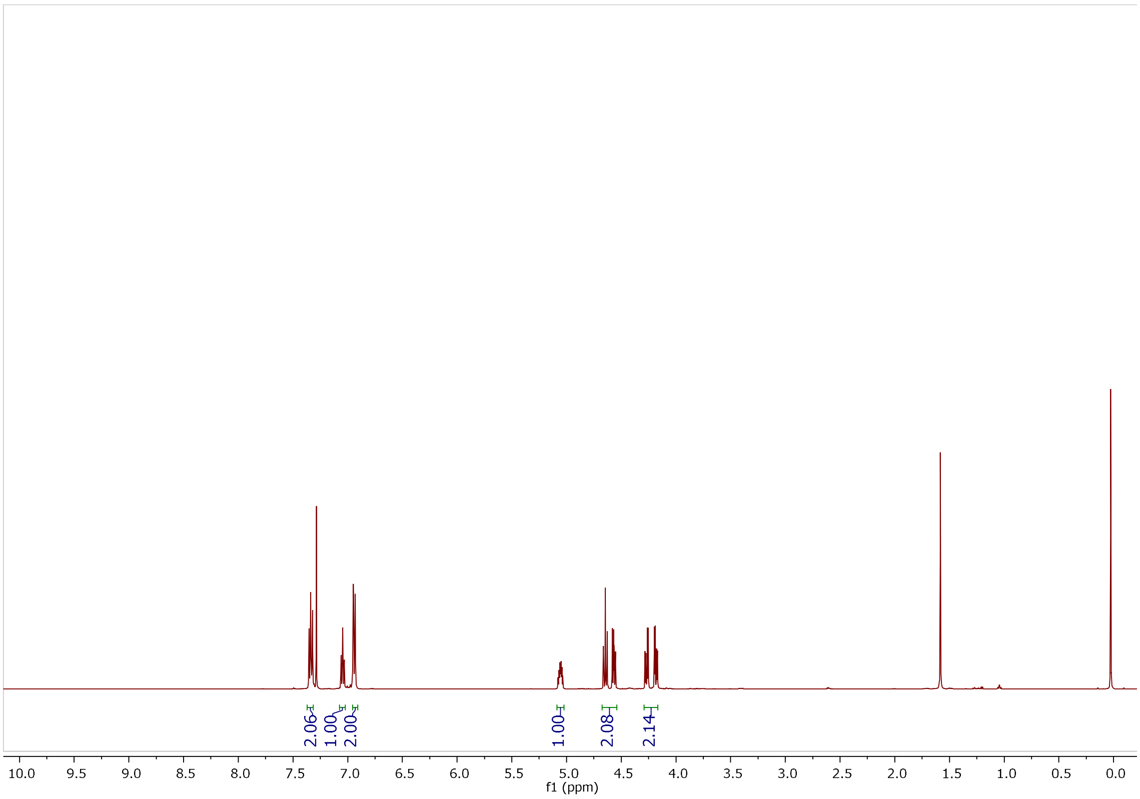

Figure S37. ^1H NMR spectrum of the reaction mixture using catalyst **13** and **EPP** (Figure 4) (500 MHz, $CDCl_3$).

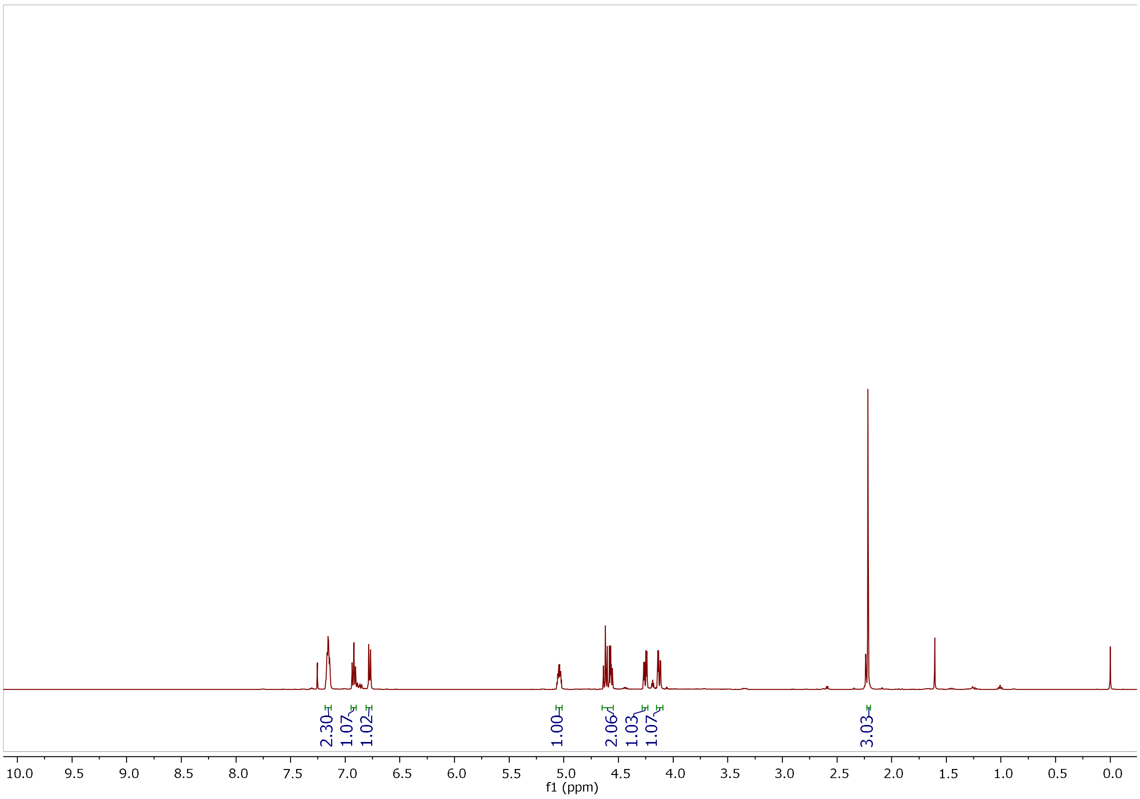

Figure S38. ^{1}H NMR spectrum of the reaction mixture using catalyst **13** and **GME** (Figure 4) (500 MHz, $CDCl_3$).

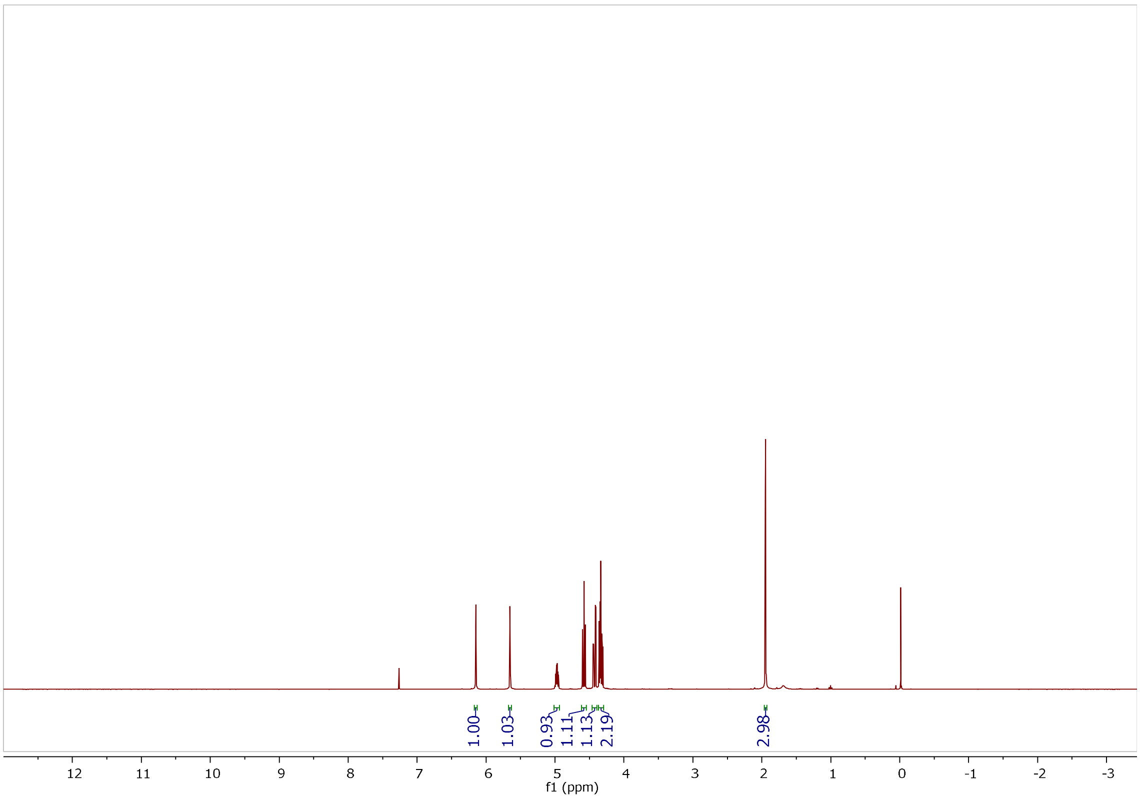

Figure S39. ^{1}H NMR spectrum of the reaction mixture using catalyst **13** and **ACR** in 3 h. (Figure 4) (500 MHz, $CDCl_3$).

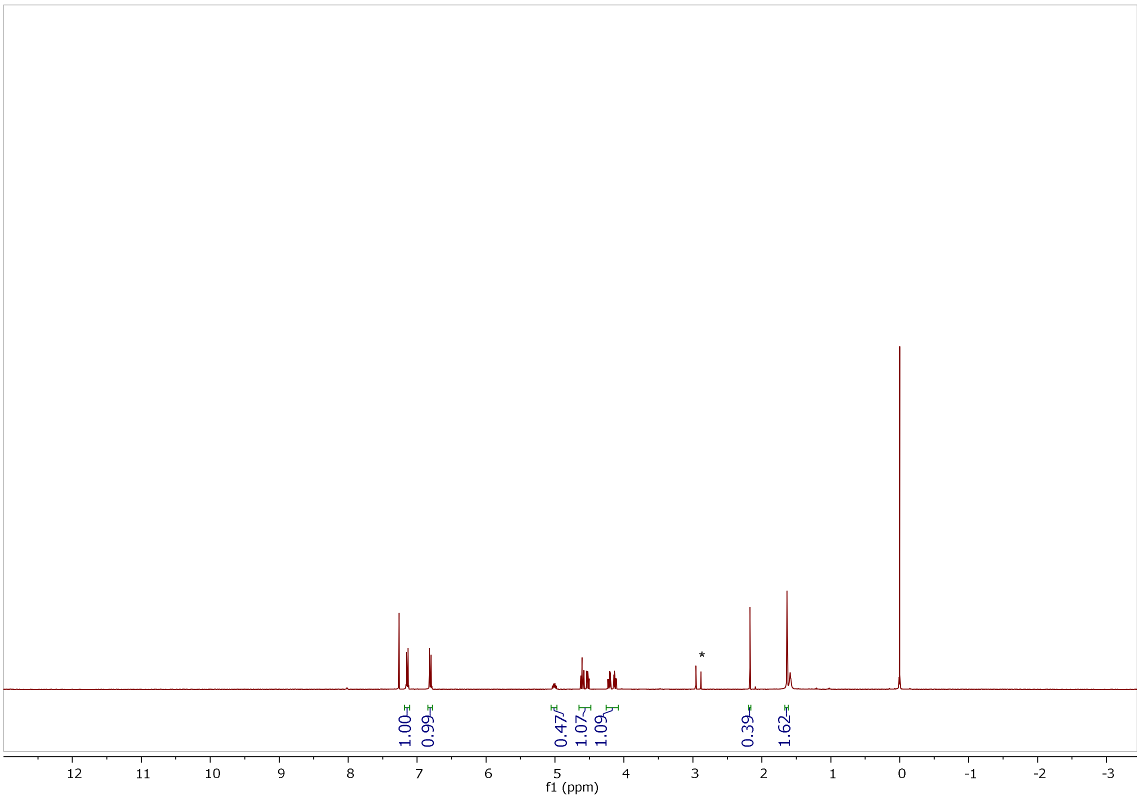

Figure S40. ^{1}H NMR spectrum of the reaction mixture using catalyst **13** and **BIS-A** in 3 h. (Figure 4) (500 MHz, $CDCl_3$). *residual solvent.

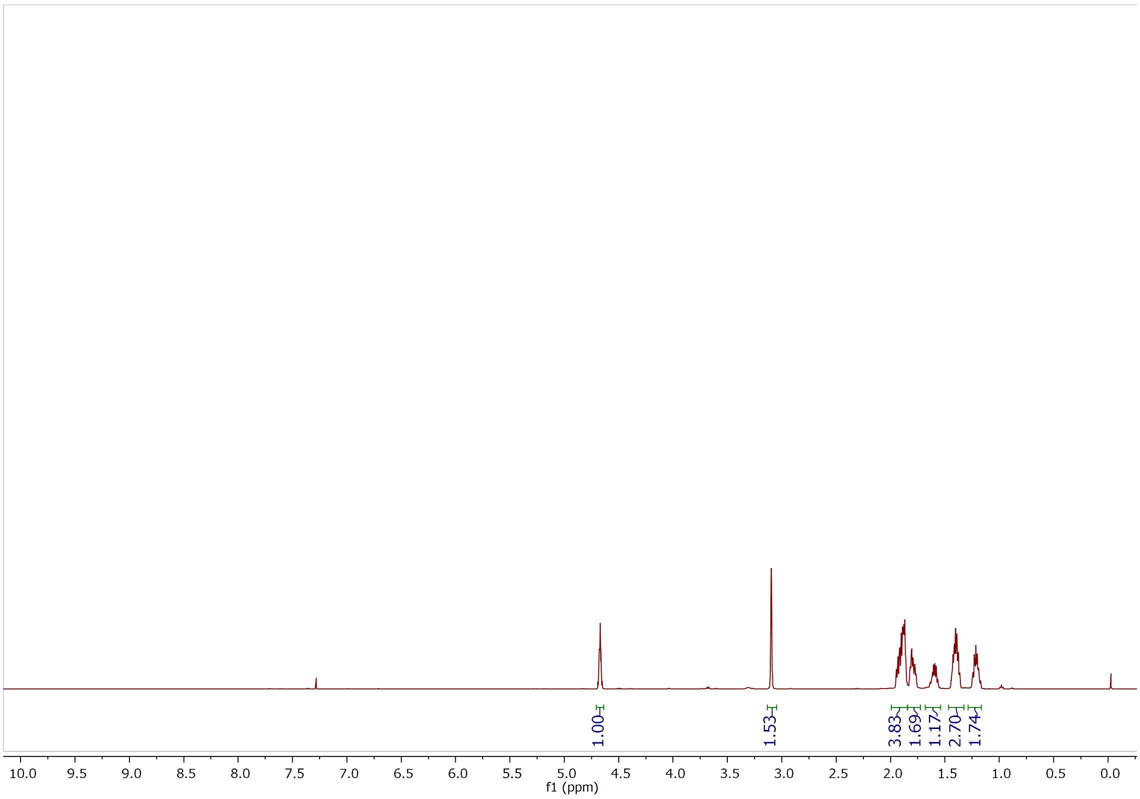

Figure S41. ^{1}H NMR spectrum of the reaction mixture using catalyst **13** and **CO** in 3 h. (Figure 4) (500 MHz, $CDCl_3$).

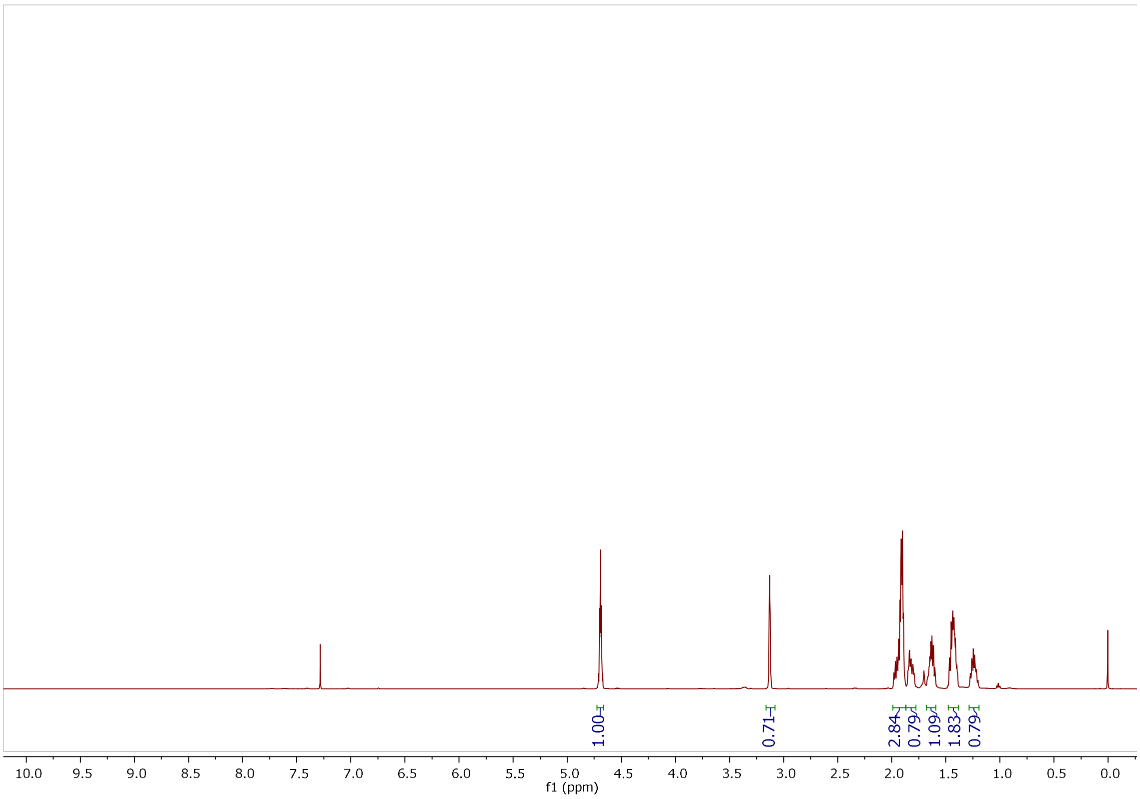

Figure S42. ^{1}H NMR spectrum of the reaction mixture using catalyst **13** and **CO** in 6 h. (Figure 4) (500 MHz, $CDCl_3$).

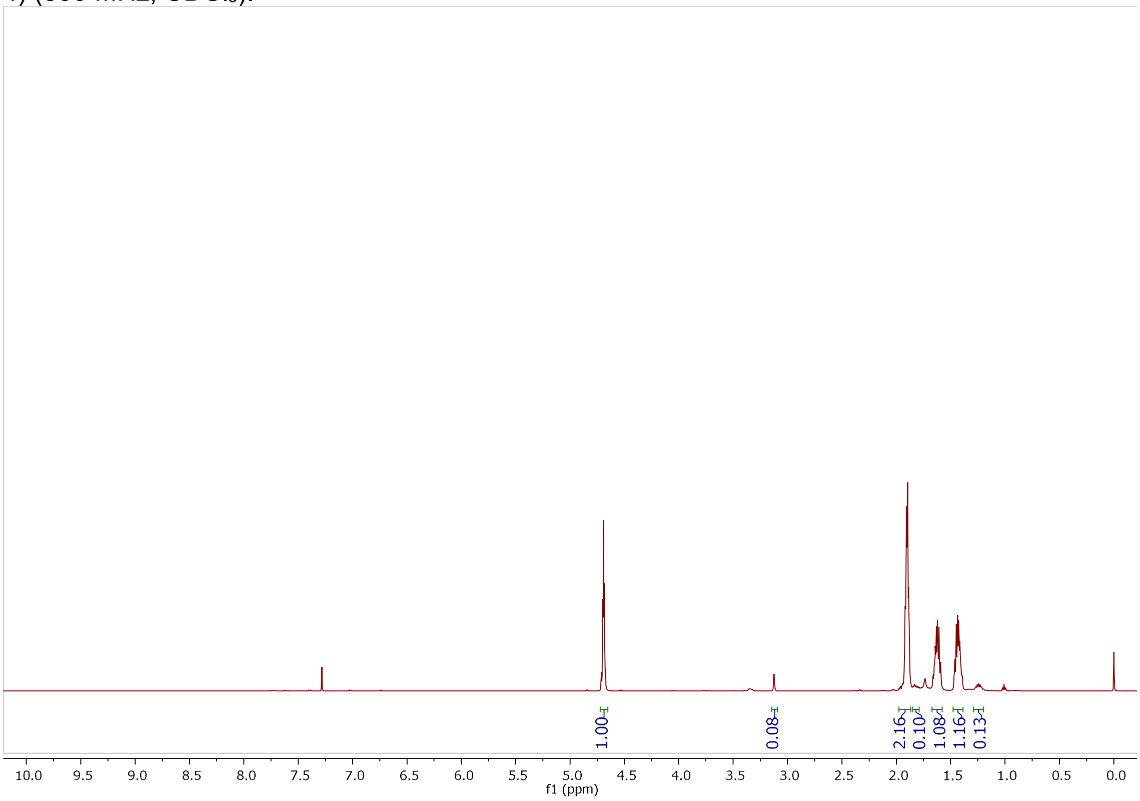

Figure S43. ^{1}H NMR spectrum of the reaction mixture using catalyst **13** and **CO** in 24 h. (Figure 4) (500 MHz, $CDCl_3$).

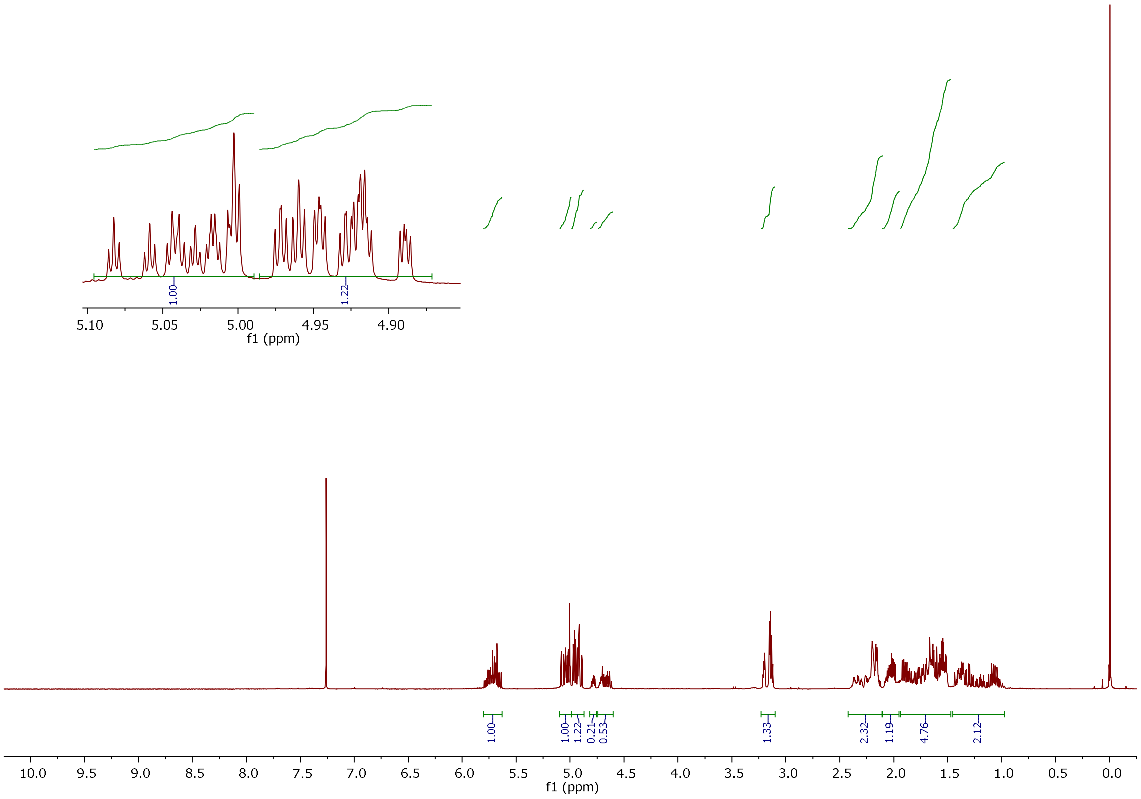

Figure S44. ^{1}H NMR spectrum of the reaction mixture using catalyst **13** and **VCO** in 3 h. (Figure 4) (500 MHz, $CDCl_3$).

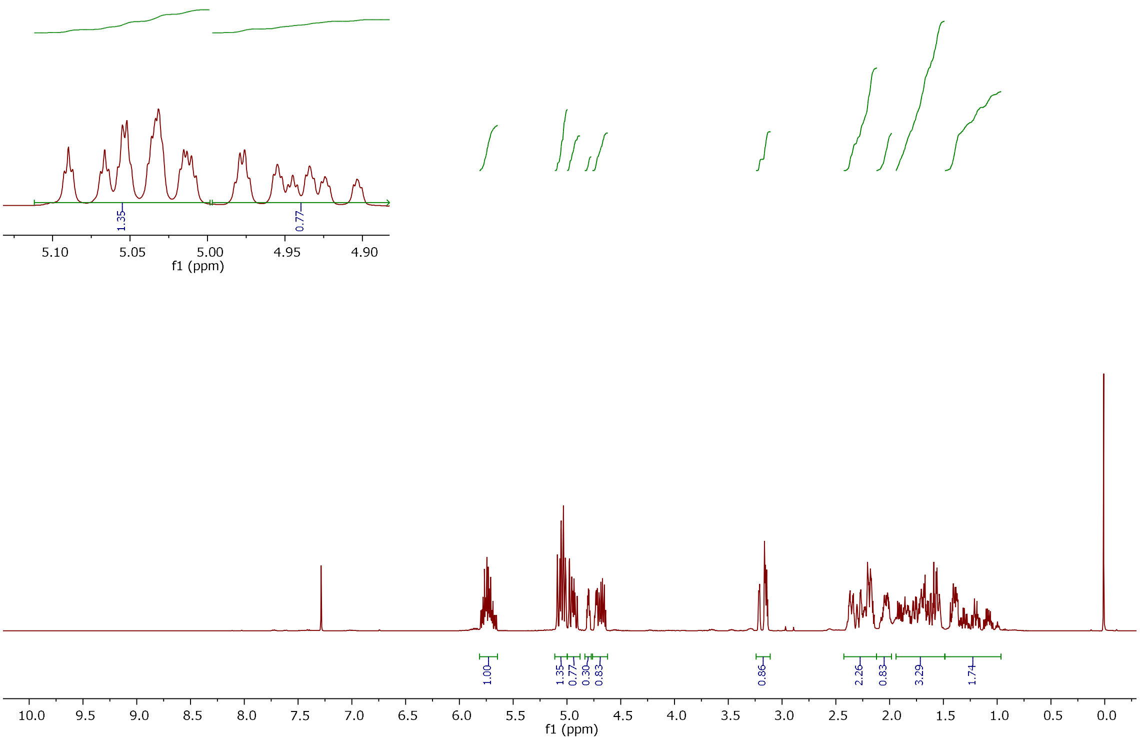

Figure S45. ^{1}H NMR spectrum of the reaction mixture using catalyst **13** and **VCO** in 6 h. (Figure 4) (500 MHz, $CDCl_3$).

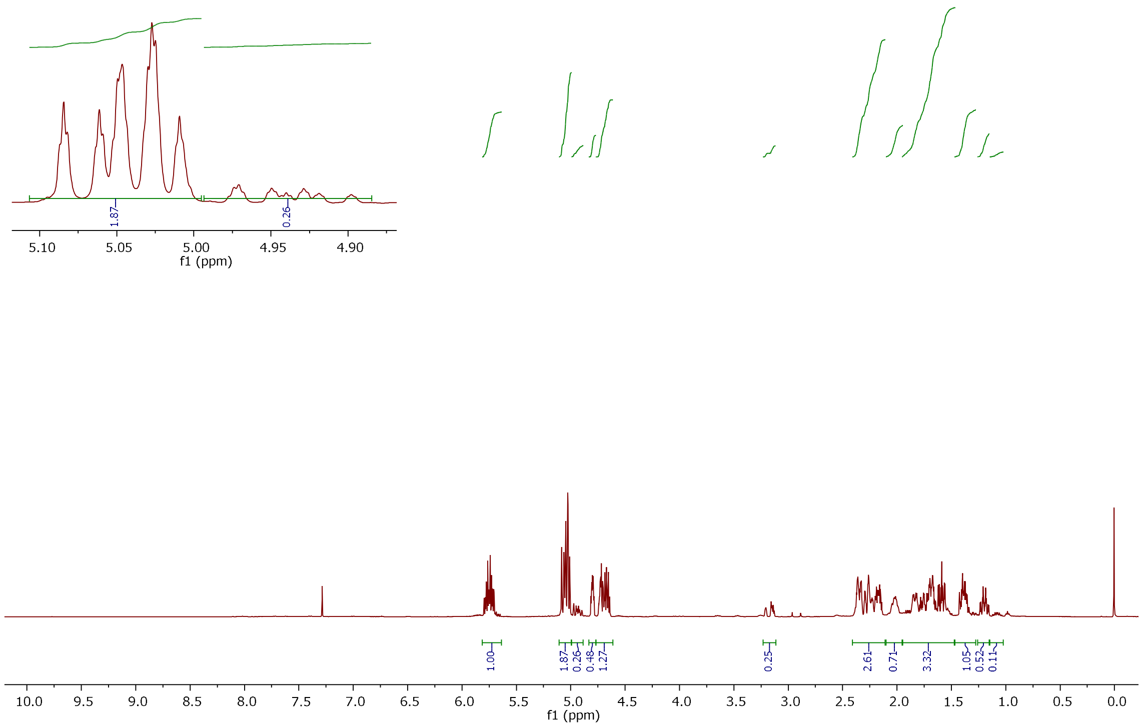

Figure S46. ^{1}H NMR spectrum of the reaction mixture using catalyst **13** and **VCO** in 24 h. (Figure 4) (500 MHz, $CDCl_3$).

5. Theoretical calculation

Here, the optimized PBEh-3c geometries are presented along with their xyz coordinates.

Reactant– **R1**
G = -1220.78387105 a.u.

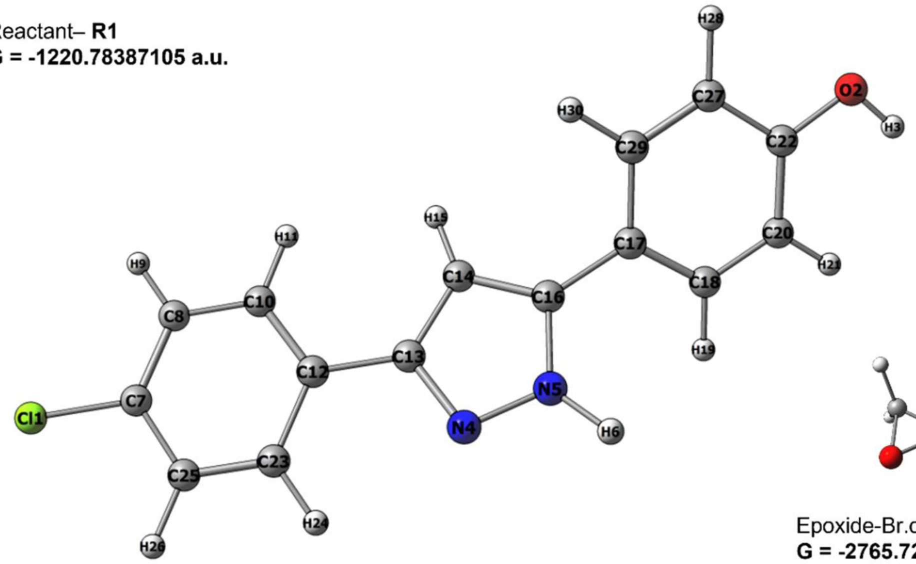

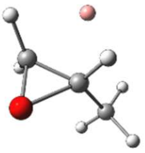

Epoxide-Br.out
G = -2765.72538121 a.u.

17	29.856447000	6.158651000	0.783009000
8	17.252342000	5.896711000	5.618790000
1	17.051605000	5.867946000	6.557634000
7	24.776598000	4.361133000	4.845364000
7	23.529956000	4.386263000	5.286554000
1	23.264764000	3.772337000	6.037126000
6	28.390891000	5.878037000	1.654805000
6	27.196000000	6.387212000	1.174786000
1	27.175764000	6.957921000	0.256721000
6	26.027120000	6.154532000	1.880116000
1	25.099765000	6.551297000	1.488004000
6	26.030068000	5.420137000	3.062951000
6	24.792709000	5.176896000	3.805105000
6	23.510848000	5.722458000	3.580980000
1	23.210450000	6.444782000	2.841098000
6	22.712318000	5.182732000	4.562978000
6	21.298082000	5.363609000	4.867975000
6	20.824732000	5.354815000	6.176910000
1	21.513817000	5.246000000	7.005593000
6	19.478198000	5.523708000	6.453571000
1	19.143429000	5.516890000	7.485169000
6	18.571854000	5.719913000	5.417061000
6	27.246629000	4.918073000	3.524358000
1	27.270025000	4.346151000	4.441878000
6	28.420800000	5.142292000	2.829465000
1	29.357176000	4.748483000	3.199677000
6	19.032746000	5.740172000	4.102991000
1	18.328306000	5.887885000	3.295314000
6	20.375055000	5.559206000	3.839394000
1	20.715705000	5.552759000	2.812125000

Epoxide-Br:

6	17.235363000	3.645257000	10.352542000
6	15.976008000	3.143716000	9.851534000
8	16.609542000	4.264975000	9.233509000
1	18.131194000	3.074492000	10.106483000
1	15.073962000	3.336898000	10.423175000
1	15.954960000	2.220151000	9.280374000
6	17.310610000	4.400960000	11.643413000
1	18.172326000	5.072218000	11.673435000
1	16.408480000	4.993000000	11.800184000
1	17.374486000	3.679373000	12.458440000
35	15.702368000	1.141339000	12.644954000

Figure S47. Optimized structure of the reactant (compound **13**) and the epoxide with its respective Gibbs energy values and its coordinates.

INT-1-epox-OH
G = -3986.52858184 a.u.

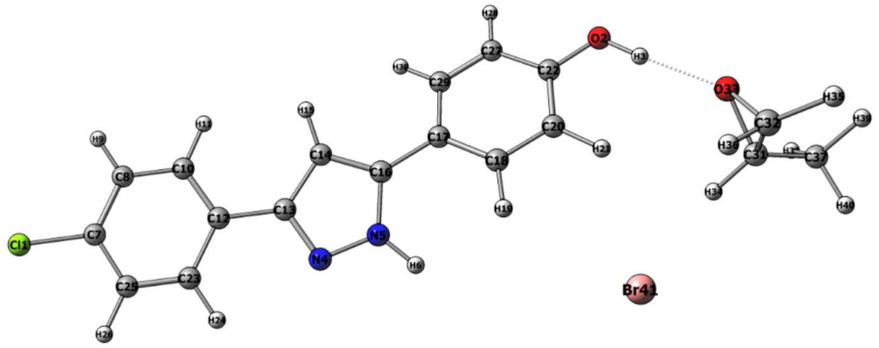

17	29.404100000	5.492600000	0.285900000
8	17.463600000	5.985800000	6.649600000
1	17.229600000	5.402500000	7.396200000
7	24.889500000	4.499500000	5.205900000
7	23.695200000	4.574200000	5.769100000
1	23.563800000	4.205400000	6.697400000
6	28.052000000	5.368500000	1.365700000
6	26.812600000	5.840000000	0.970100000
1	26.684300000	6.283100000	-0.007900000
6	25.738300000	5.737200000	1.838900000
1	24.774200000	6.108000000	1.516800000
6	25.878300000	5.169000000	3.103300000
6	24.744300000	5.067600000	4.020300000
6	23.419300000	5.509700000	3.836500000
1	22.987500000	5.987400000	2.973400000
6	22.758400000	5.172400000	5.000400000
6	21.380800000	5.363000000	5.430600000
6	20.850700000	4.648800000	6.504300000
1	21.412300000	3.902400000	7.057600000
6	19.553100000	4.847000000	6.925700000
1	19.224300000	4.268700000	7.780700000
6	18.729600000	5.762600000	6.276200000
6	27.139800000	4.699900000	3.474000000
1	27.267000000	4.254700000	4.451400000
6	28.221100000	4.796900000	2.617200000
1	29.192000000	4.430500000	2.921500000
6	19.245000000	6.480300000	5.193700000
1	18.615300000	7.199600000	4.685500000
6	20.551100000	6.283000000	4.787100000
1	20.936600000	6.868700000	3.961200000
6	17.228400000	3.982900000	10.048500000
6	16.934200000	2.942200000	9.077600000
8	16.687900000	4.313500000	8.767000000
1	18.285300000	4.216300000	10.169600000
1	16.038200000	2.334300000	9.186100000
1	17.776000000	2.473000000	8.575400000
6	16.343100000	4.261300000	11.224600000
1	16.329400000	5.325900000	11.463300000
1	15.316200000	3.941300000	11.043200000
1	16.718200000	3.730900000	12.100100000
35	20.650500000	2.491300000	9.494400000

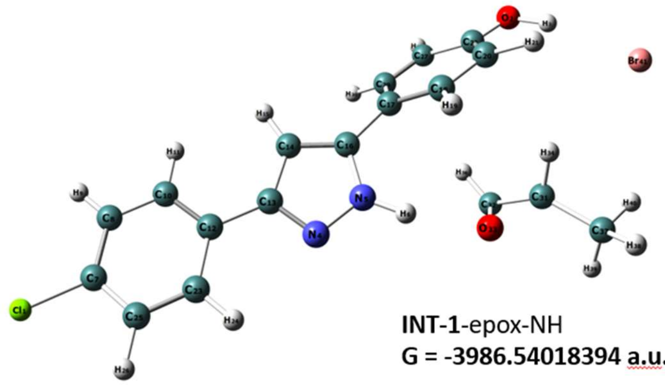

INT-1-epox-NH
G = -3986.54018394 a.u.

17	29.302800000	6.098600000	-0.083900000
8	18.265400000	5.060100000	7.828700000
1	18.254800000	4.356900000	8.527800000
7	25.268900000	4.266900000	5.010900000
7	24.125000000	4.190700000	5.666800000
1	24.088100000	3.800000000	6.604800000
6	28.049800000	5.752000000	1.064600000
6	26.760100000	6.193800000	0.826700000
1	26.530600000	6.749200000	-0.072300000
6	25.767000000	5.920200000	1.752800000
1	24.763100000	6.272600000	1.555600000
6	26.037000000	5.209800000	2.920700000
6	24.990300000	4.937500000	3.903700000
6	23.628000000	5.300400000	3.868000000
1	23.087600000	5.824500000	3.097800000
6	23.096300000	4.797000000	5.035000000
6	21.765000000	4.830900000	5.628800000
6	21.236900000	3.691900000	6.236800000
1	21.767800000	2.750600000	6.160900000
6	20.053200000	3.734700000	6.944200000
1	19.679300000	2.847200000	7.438700000
6	19.360300000	4.942900000	7.086900000
6	27.345800000	4.771200000	3.130700000
1	27.574900000	4.215600000	4.029800000
6	28.347300000	5.037400000	2.214800000
1	29.356400000	4.692800000	2.394900000
6	19.843400000	6.069800000	6.412700000
1	19.302200000	7.003400000	6.499200000
6	21.028900000	6.011800000	5.702100000
1	21.417900000	6.911300000	5.240000000
6	22.489200000	3.407100000	9.450500000
6	22.937000000	4.752700000	9.127200000
8	23.632300000	3.623400000	8.614600000
1	21.603900000	3.046600000	8.936100000
1	23.478300000	5.333800000	9.871300000
1	22.379100000	5.341700000	8.403300000
6	22.722700000	2.778100000	10.787300000
1	22.933600000	1.713000000	10.683500000
1	23.551700000	3.241400000	11.324400000
1	21.808600000	2.874600000	11.373300000
35	18.755000000	2.855600000	10.196000000

Figure S48. Optimized structures of the intermediate INT-1 considering a supermolecule approach, its Gibbs energy values and coordinates.

TS1 – imag. freq. = -475.38i cm^{-1}
G = -3986.50619802 a.u.

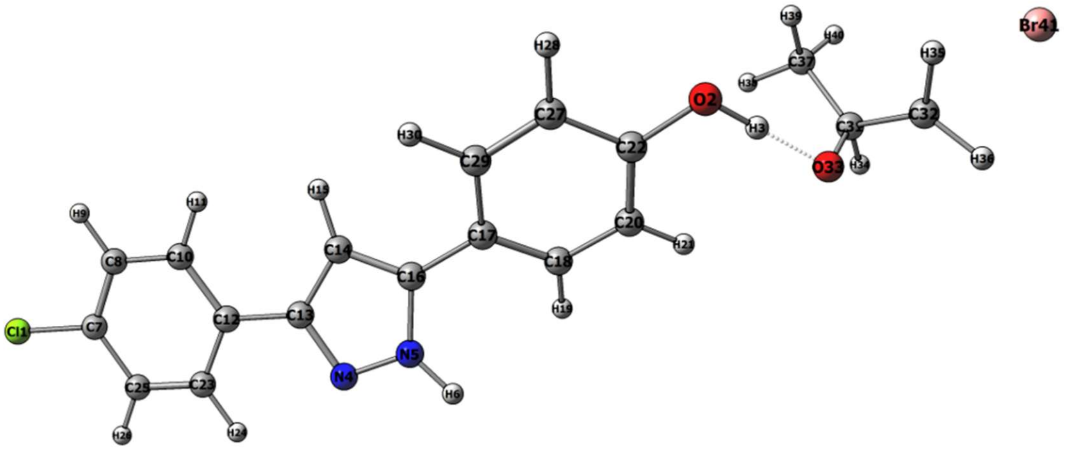

17	29.408100000	7.089700000	0.605700000
8	17.651800000	4.455300000	6.749100000
1	17.526900000	4.005300000	7.796400000
7	24.980400000	3.755300000	4.458600000
7	23.804300000	3.564200000	5.040500000
1	23.644600000	2.723900000	5.567900000
6	28.085800000	6.453100000	1.529100000
6	26.872900000	7.119000000	1.538100000
1	26.748000000	8.032500000	0.973200000
6	25.820700000	6.603700000	2.277000000
1	24.877300000	7.133400000	2.273500000
6	25.957100000	5.428800000	3.013200000
6	24.841800000	4.886200000	3.787300000
6	23.546100000	5.415400000	3.952200000
1	23.140700000	6.334800000	3.565600000
6	22.889100000	4.525000000	4.778100000
6	21.538800000	4.504500000	5.310200000
6	21.236900000	3.927600000	6.548100000
1	22.032600000	3.515200000	7.161100000
6	19.953300000	3.899400000	7.046500000
1	19.746100000	3.456900000	8.012600000
6	18.875100000	4.462500000	6.321800000
6	27.192800000	4.780100000	2.987300000
1	27.317700000	3.867700000	3.554300000
6	28.251700000	5.282700000	2.253200000
1	29.202200000	4.766700000	2.243600000
6	19.191400000	5.049600000	5.075000000
1	18.387400000	5.484500000	4.494500000
6	20.480900000	5.065800000	4.590900000
1	20.676400000	5.505600000	3.619200000
6	16.887800000	4.160300000	9.921300000
6	15.421800000	3.583200000	9.930900000
8	17.500300000	3.470200000	8.905500000
1	17.334200000	3.939300000	10.911900000
1	15.020600000	3.768600000	8.881200000
1	15.543500000	2.449700000	10.033100000
6	16.893700000	5.693300000	9.791500000
1	17.924100000	6.053900000	9.759400000
1	16.408900000	6.005000000	8.862800000
1	16.386100000	6.179200000	10.626000000
35	13.914800000	4.216700000	11.338900000

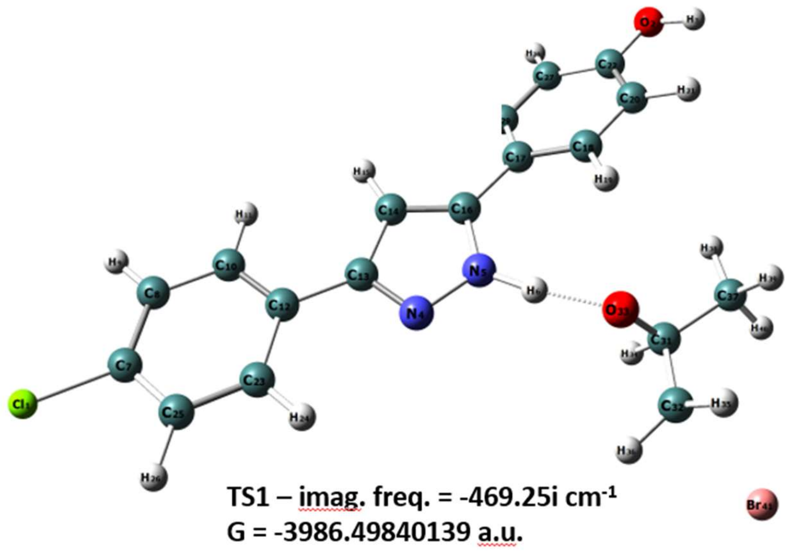

17	29.331600000	6.894500000	0.454600000
8	17.669500000	4.805600000	6.886100000
1	17.337700000	3.940200000	7.136900000
7	24.874800000	3.605100000	4.333500000
7	23.727000000	3.418100000	4.952000000
1	23.566500000	2.519800000	5.512500000
6	28.015700000	6.286300000	1.410000000
6	26.851200000	7.025400000	1.521300000
1	26.759000000	7.977400000	1.016500000
6	25.804600000	6.532400000	2.283000000
1	24.899000000	7.120400000	2.357900000
6	25.895900000	5.307300000	2.941900000
6	24.787400000	4.787100000	3.737700000
6	23.535500000	5.370800000	3.992000000
1	23.148700000	6.310000000	3.632900000
6	22.873900000	4.450700000	4.783400000
6	21.526100000	4.505100000	5.345100000
6	20.826500000	3.357800000	5.716600000
1	21.285700000	2.381900000	5.621500000
6	19.545000000	3.442300000	6.235100000
1	19.035600000	2.527300000	6.520700000
6	18.922800000	4.674600000	6.388300000
6	27.084400000	4.585400000	2.812900000
1	27.174900000	3.633500000	3.318500000
6	28.137700000	5.065600000	2.055600000
1	29.050600000	4.492300000	1.968200000
6	19.606700000	5.828700000	6.022800000
1	19.134200000	6.793700000	6.152600000
6	20.888800000	5.735600000	5.514500000
1	21.414800000	6.647000000	5.259900000
6	23.247000000	1.260100000	7.676500000
6	24.121100000	0.145900000	7.362200000
8	23.159800000	1.248900000	6.284200000
1	23.769800000	2.126700000	8.101000000
1	23.684800000	-0.808900000	7.118100000
1	25.136600000	0.336700000	7.057200000
6	21.968500000	0.984600000	8.429400000
1	21.296000000	1.842800000	8.371200000
1	21.455300000	0.115600000	8.013900000
1	22.191800000	0.780500000	9.476100000
35	24.969600000	-0.824900000	9.572400000

Figure S49. Optimized structures of the transition state TS1 with the epoxide opening, its Gibbs energy values and coordinates.

INT-2-OH
G = -3986.53860469 a.u.

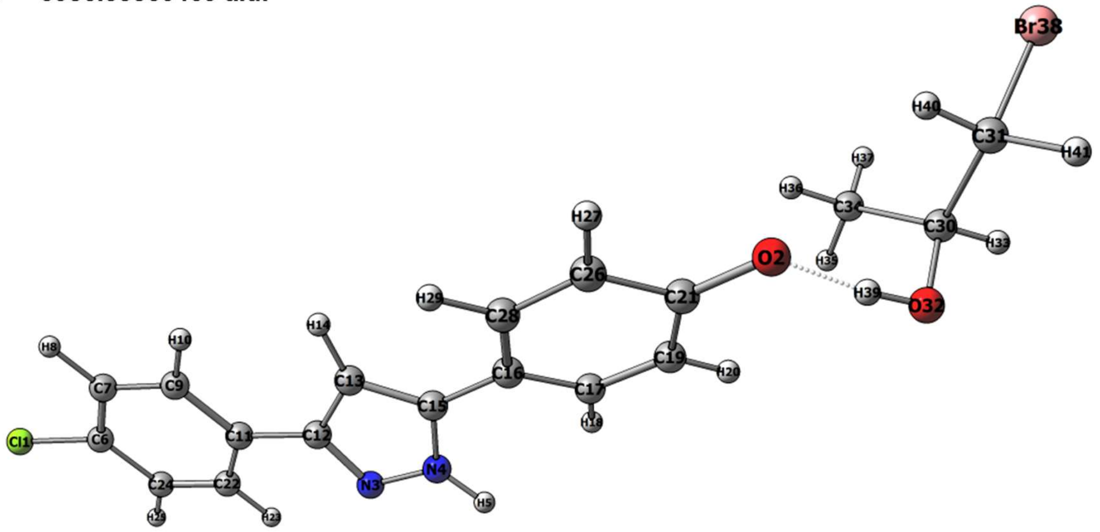

17	28.916700000	7.126300000	0.165900000
8	17.797200000	3.663500000	7.077800000
7	25.201400000	4.259600000	5.009400000
7	24.070600000	3.964200000	5.640400000
1	24.097200000	3.343800000	6.429600000
6	27.728400000	6.523400000	1.276600000
6	26.384700000	6.748300000	1.034500000
1	26.073000000	7.300200000	0.158200000
6	25.442800000	6.256100000	1.923600000
1	24.394700000	6.431600000	1.720000000
6	25.818900000	5.539500000	3.057900000
6	24.823700000	5.011700000	3.989600000
6	23.426400000	5.191700000	3.980800000
1	22.830900000	5.779900000	3.303800000
6	22.956500000	4.491300000	5.078900000
6	21.627200000	4.288000000	5.612800000
6	21.403700000	3.941800000	6.953200000
1	22.242900000	3.863300000	7.639400000
6	20.141400000	3.731100000	7.453700000
1	20.015100000	3.469800000	8.498100000
6	18.970600000	3.857200000	6.641200000
6	27.182100000	5.332800000	3.277800000
1	27.492600000	4.781700000	4.155000000
6	28.133000000	5.817000000	2.398400000
1	29.184900000	5.647300000	2.583700000
6	19.223300000	4.222900000	5.281700000
1	18.370500000	4.326500000	4.621300000
6	20.493600000	4.423900000	4.799600000
1	20.624700000	4.673500000	3.751600000
6	16.801100000	4.014000000	10.142600000
6	15.462400000	4.271100000	9.447900000
8	17.383700000	2.912000000	9.524400000
1	16.593500000	3.731800000	11.182100000
6	17.708400000	5.239200000	10.144300000
1	18.661700000	4.985700000	10.609100000
1	17.913400000	5.569400000	9.125000000
1	17.275100000	6.072900000	10.697400000
35	14.335600000	5.595500000	10.391400000
1	17.596100000	3.157100000	8.572300000
1	15.610500000	4.643400000	8.433600000
1	14.877700000	3.353100000	9.412200000

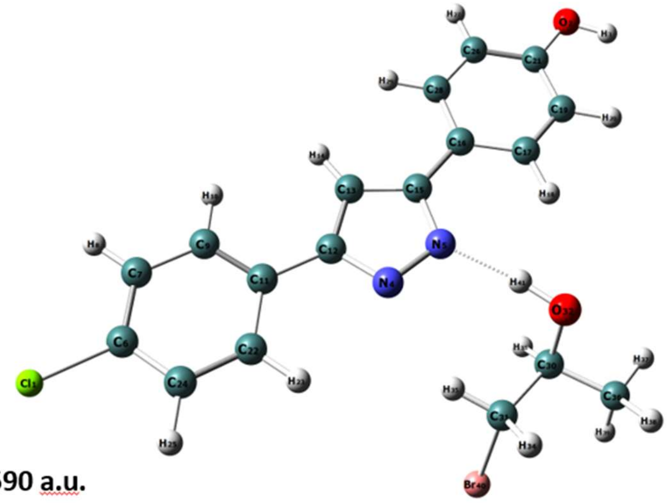

INT-2-NH
G = -3986.53367590 a.u.

17	29.692100000	5.701900000	1.069900000
8	17.129800000	5.706000000	6.165300000
1	16.855800000	5.134100000	6.885600000
7	24.489500000	3.793000000	4.963300000
7	23.267200000	3.782000000	5.453600000
6	28.203500000	5.436500000	1.931700000
6	27.082200000	6.184900000	1.618000000
1	27.127100000	6.933900000	0.838500000
6	25.901800000	5.964500000	2.308900000
1	25.031600000	6.554200000	2.050700000
6	25.808300000	5.003200000	3.318700000
6	24.568800000	4.761800000	4.040500000
6	23.333700000	5.407200000	3.933300000
1	23.063300000	6.232200000	3.293000000
6	22.534700000	4.744100000	4.859900000
6	21.136400000	4.976600000	5.208300000
6	20.508700000	4.271700000	6.238000000
1	21.065200000	3.530500000	6.795600000
6	19.182200000	4.500100000	6.565500000
1	18.735100000	3.927300000	7.373100000
6	18.434600000	5.446300000	5.875900000
6	26.962000000	4.266300000	3.610700000
1	26.914600000	3.519200000	4.391000000
6	28.146500000	4.475400000	2.929000000
1	29.024700000	3.893200000	3.173600000
6	19.038100000	6.159200000	4.848900000
1	18.464600000	6.897800000	4.302900000
6	20.363300000	5.921100000	4.528000000
1	20.806500000	6.488800000	3.719500000
6	23.980600000	2.328400000	8.312700000
6	25.346400000	2.025300000	7.694800000
8	23.015400000	1.914200000	7.393200000
1	23.925400000	3.411900000	8.496800000
1	25.473300000	0.952400000	7.543600000
1	25.458100000	2.550000000	6.743500000
6	23.737500000	1.591400000	9.618000000
1	22.727800000	1.795500000	9.976000000
1	23.829900000	0.512900000	9.472900000
1	24.439700000	1.893000000	10.393800000
35	26.853400000	2.612900000	8.832800000
1	23.042300000	2.549900000	6.623200000

Figure S50. Optimized structures of the intermediate INT-2 considering a supermolecule approach and its Gibbs energy values. Coordinates are also shown. One can be seen the proton transfer to the epoxide.

INT-2-CO2
G = -4174.73306310 a.u.

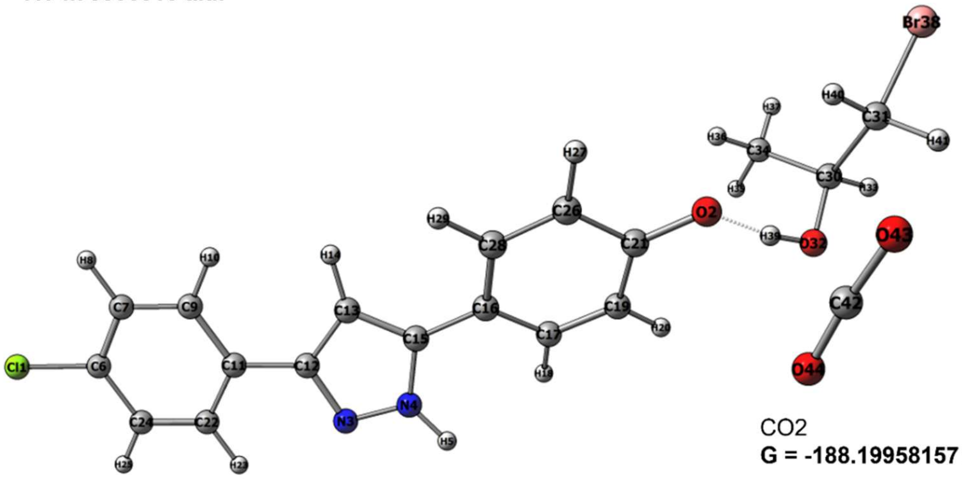

CO2
G = -188.19958157 a.u.

17	28.793900000	7.031400000	0.058500000
8	17.894500000	3.864800000	7.448700000
7	25.167800000	4.093600000	4.921900000
7	24.061300000	3.838300000	5.609000000
1	24.080000000	3.136500000	6.327300000
6	27.635600000	6.449400000	1.210900000
6	26.331100000	6.908400000	1.165700000
1	26.032400000	7.631100000	0.418800000
6	25.411100000	6.431000000	2.085200000
1	24.393100000	6.794600000	2.038500000
6	25.771500000	5.497600000	3.054600000
6	24.797000000	4.979200000	4.012900000
6	23.426900000	5.285900000	4.133000000
1	22.843900000	5.988500000	3.562200000
6	22.968600000	4.518100000	5.188700000
6	21.661700000	4.369600000	5.795400000
6	21.498400000	3.963400000	7.126700000
1	22.370800000	3.801400000	7.753600000
6	20.255000000	3.796200000	7.689500000
1	20.169400000	3.488500000	8.725000000
6	19.056100000	4.027000000	6.953000000
6	27.096400000	5.058400000	3.079700000
1	27.394000000	4.335800000	3.827200000
6	28.025000000	5.525500000	2.168100000
1	29.046700000	5.172200000	2.199400000
6	19.245600000	4.456700000	5.606400000
1	18.362700000	4.645000000	5.007300000
6	20.496500000	4.616200000	5.059200000
1	20.583500000	4.917600000	4.020600000
6	16.437200000	4.582600000	10.248300000
6	15.184700000	4.368100000	9.395700000
8	17.326600000	3.549400000	9.956000000
1	16.152900000	4.476800000	11.302100000
6	17.061600000	5.958300000	10.043400000
1	17.974700000	6.036000000	10.634300000
1	17.327700000	6.105100000	8.995600000
1	16.393500000	6.764700000	10.345600000
35	13.689900000	5.553900000	9.915500000
1	17.649400000	3.677700000	9.016500000
1	15.386700000	4.553900000	8.340500000
1	14.815000000	3.351200000	9.513600000
6	17.096700000	1.276000000	7.757000000
8	16.005000000	1.526900000	7.465400000
8	18.155300000	0.911000000	8.044100000

CO_2:

6	-1.263500000	-0.594600000	0.000000000
8	-0.108100000	-0.594600000	0.000000000
8	-2.418900000	-0.594600000	0.000000000

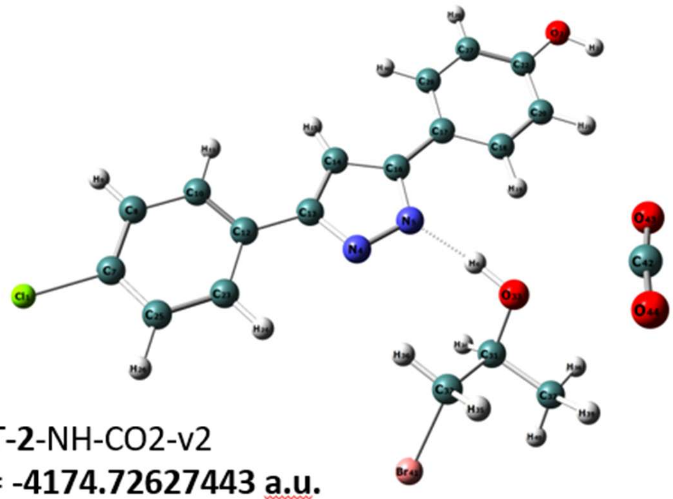

INT-2-NH-CO2-v2
G = -4174.72627443 a.u.

17	29.759600000	6.354200000	1.599500000
8	17.058900000	5.475200000	6.282600000
1	16.732200000	4.709800000	6.760500000
7	24.347600000	3.638200000	4.628700000
7	23.116400000	3.555800000	5.089000000
1	22.802700000	2.092800000	5.776600000
6	28.236800000	5.922200000	2.323100000
6	27.159300000	6.784800000	2.222400000
1	27.260600000	7.725400000	1.697600000
6	25.948700000	6.431100000	2.794900000
1	25.111500000	7.110900000	2.701300000
6	25.782700000	5.223800000	3.477800000
6	24.510900000	4.846200000	4.074200000
6	23.323300000	5.576400000	4.183700000
1	23.125400000	6.583100000	3.850800000
6	22.460000000	4.706000000	4.842400000
6	21.067200000	4.897600000	5.237000000
6	20.373600000	3.931500000	5.968500000
1	20.874800000	3.025200000	6.276300000
6	19.045400000	4.105900000	6.319900000
1	18.545000000	3.321000000	6.879600000
6	18.363900000	5.261200000	5.959800000
6	26.894500000	4.377600000	3.564600000
1	26.795200000	3.439300000	4.093600000
6	28.108400000	4.717200000	2.996100000
1	28.953200000	4.046400000	3.078100000
6	19.036000000	6.241200000	5.241400000
1	18.515600000	7.146600000	4.955300000
6	20.361600000	6.052600000	4.889400000
1	20.860000000	6.826700000	4.319900000
6	23.879200000	0.982500000	6.992700000
6	24.986200000	0.637600000	5.997500000
8	22.717200000	1.204600000	6.249000000
1	24.181100000	1.891000000	7.533400000
1	24.756800000	-0.288200000	5.468500000
1	25.119800000	1.456500000	5.288200000
6	23.605400000	-0.124900000	7.995700000
1	22.756800000	0.147100000	8.624600000
1	23.362900000	-1.059400000	7.484900000
1	24.460700000	-0.307600000	8.645000000
35	26.740800000	0.375800000	6.868300000
6	20.813900000	-0.496800000	5.534100000
8	19.985600000	0.214600000	5.914500000
8	21.559800000	-1.283400000	5.129700000

Figure S51. Optimized structure of the intermediate INT2-CO2 considering a supermolecule approach with the presence of carbon dioxide and its Gibbs energy values. Coordinates are also shown.

TS2 – imag. freq. = -251.79i cm^{-1}
G = -4174.72492518 a.u.

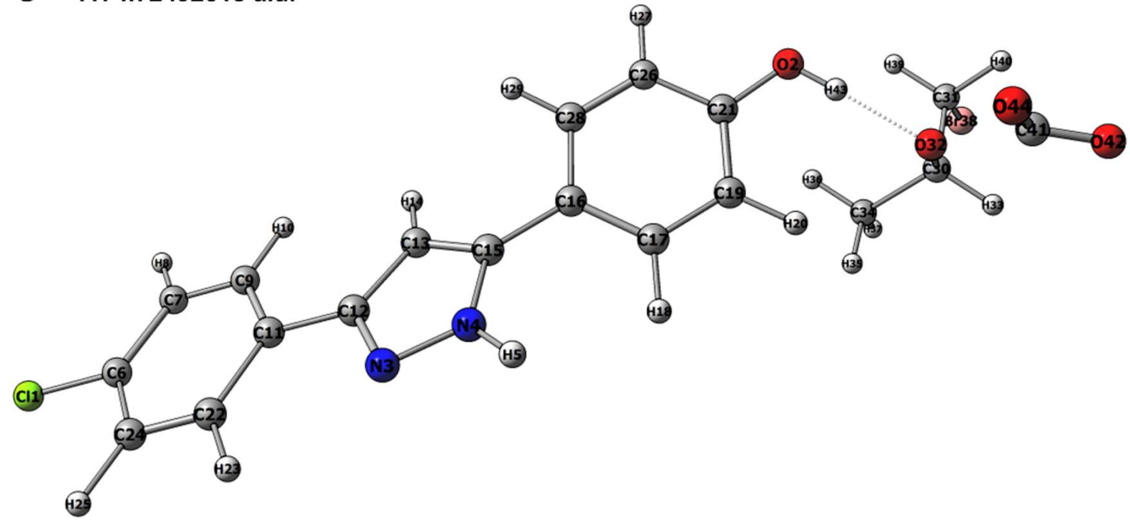

17	29.842700000	6.907800000	1.356200000
8	17.334300000	7.212400000	6.518200000
7	23.867700000	4.547900000	3.410200000
7	22.647500000	4.653100000	3.921600000
1	22.071300000	3.833600000	3.996400000
6	28.240600000	6.585100000	1.938300000
6	27.415800000	7.638100000	2.291500000
1	27.764800000	8.657800000	2.203100000
6	26.138600000	7.373300000	2.758000000
1	25.500500000	8.204500000	3.027400000
6	25.666300000	6.068100000	2.879900000
6	24.319900000	5.790000000	3.375000000
6	23.361400000	6.693800000	3.874400000
1	23.449500000	7.758000000	4.012600000
6	22.273300000	5.917300000	4.227900000
6	20.987900000	6.240900000	4.809700000
6	20.298300000	5.335800000	5.625800000
1	20.743700000	4.373100000	5.860900000
6	19.081600000	5.639400000	6.185600000
1	18.591400000	4.908200000	6.815000000
6	18.446100000	6.903600000	5.983100000
6	26.520800000	5.026500000	2.515000000
1	26.171100000	4.007100000	2.605200000
6	27.797800000	5.275900000	2.047000000
1	28.447700000	4.456900000	1.770000000
6	19.162100000	7.803500000	5.137900000
1	18.715500000	8.770100000	4.937800000
6	20.378800000	7.480600000	4.583500000
1	20.872300000	8.199200000	3.937200000
6	16.610500000	6.098400000	9.369800000
6	15.684100000	7.294600000	9.582900000
8	16.495200000	5.752400000	8.040300000
1	16.229400000	5.270200000	9.985300000
6	18.055000000	6.354600000	9.768700000
1	18.652400000	5.474900000	9.527600000
1	18.468800000	7.194800000	9.208500000
1	18.168500000	6.559400000	10.833600000
35	15.578700000	7.886200000	11.461300000
1	16.021700000	8.153100000	9.000300000
1	14.665700000	7.040300000	9.291600000
6	14.794400000	4.234900000	7.422100000
8	14.331900000	4.195400000	8.494700000
1	16.848800000	6.345500000	7.454000000
8	15.168600000	4.186600000	6.327800000

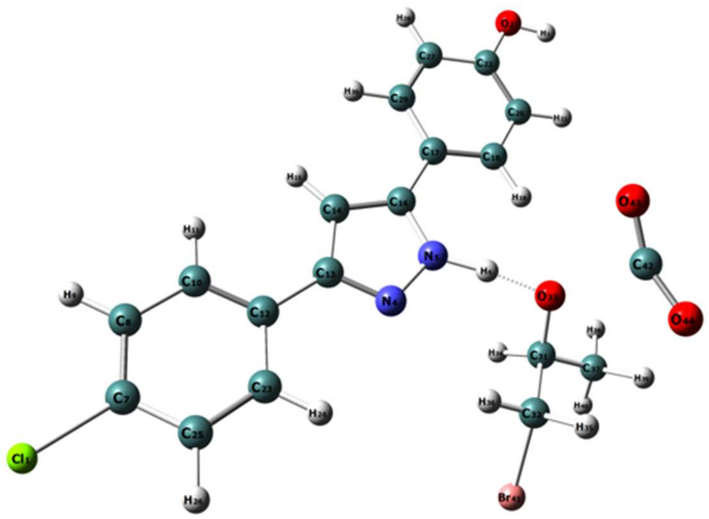

TS2-NH – imag. freq. = -235.44i cm⁻¹
G = -4174.71504578 a.u.

17	29.644300000	6.337400000	1.515400000
8	17.113300000	5.224700000	6.496500000
1	16.793400000	4.456200000	6.974500000
7	24.380300000	3.438500000	4.595800000
7	23.159600000	3.349200000	5.081000000
1	22.872100000	2.371000000	5.562400000
6	28.157500000	5.858900000	2.276600000
6	27.095400000	6.744500000	2.320900000
1	27.189200000	7.730700000	1.886800000
6	25.910100000	6.353700000	2.922500000
1	25.084400000	7.053100000	2.946500000
6	25.761700000	5.087300000	3.486900000
6	24.510500000	4.667600000	4.109400000
6	23.321000000	5.387800000	4.295600000
1	23.112900000	6.408800000	4.021100000
6	22.468900000	4.496800000	4.928500000
6	21.086200000	4.666600000	5.368000000
6	20.428500000	3.700600000	6.129700000
1	20.939300000	2.795600000	6.432500000
6	19.108700000	3.873400000	6.510500000
1	18.626900000	3.092100000	7.089900000
6	18.408800000	5.017300000	6.148700000
6	26.854800000	4.218400000	3.432700000
1	26.762400000	3.234000000	3.871200000
6	28.043300000	4.594700000	2.833900000
1	28.877700000	3.907300000	2.801800000
6	19.051400000	5.994300000	5.398200000
1	18.513500000	6.887500000	5.107200000
6	20.367600000	5.810600000	5.016100000
1	20.842500000	6.575800000	4.415500000
6	23.714700000	0.903900000	7.084100000
6	24.848200000	0.346200000	6.217500000
8	22.647800000	1.175900000	6.244000000
1	24.088200000	1.842500000	7.532300000
1	24.590700000	-0.622400000	5.792900000
1	25.087700000	1.051700000	5.423000000
6	23.323400000	-0.018500000	8.236000000
1	22.456200000	0.399800000	8.749500000
1	23.057400000	-1.014100000	7.881700000
1	24.126000000	-0.121300000	8.967300000
35	26.546300000	0.081000000	7.212500000
6	21.502200000	-0.232600000	5.341500000
8	20.619600000	0.449500000	4.982100000
8	22.092600000	-1.240400000	5.474800000

Figure S52. Optimized structures of the transition state TS2 with proton return back to oxygen of the hydroxyl group and the interaction (bending) of the CO_2. Coordinates are also shown.

INT-3-OH
G = -4174.73707047 a.u.

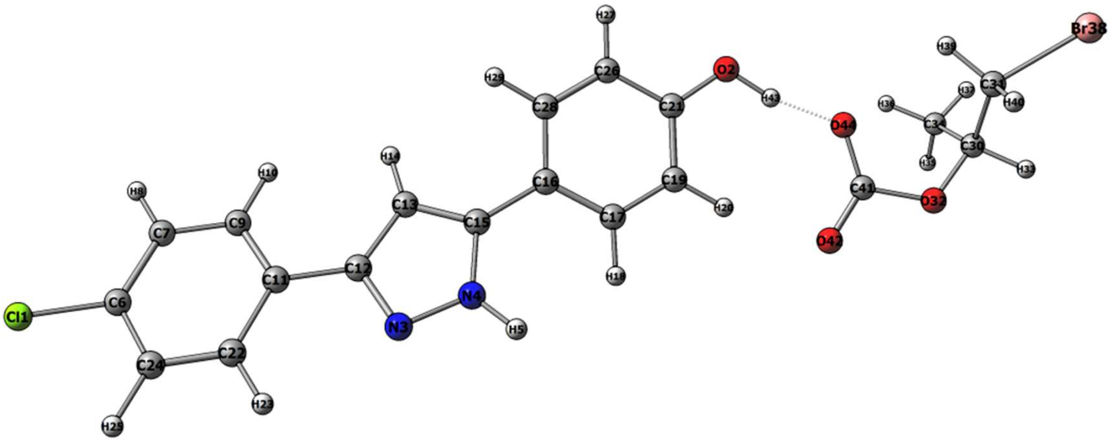

17	29.604100000	6.193800000	0.271100000
8	18.037900000	7.418000000	7.153300000
7	24.254700000	4.237900000	3.887200000
7	23.131500000	4.425400000	4.564300000
1	22.647900000	3.630800000	4.945400000
6	28.150000000	5.977000000	1.189500000
6	27.329600000	7.062800000	1.440500000
1	27.590000000	8.042300000	1.063700000
6	26.171900000	6.881900000	2.179500000
1	25.537200000	7.738000000	2.366700000
6	25.815800000	5.630100000	2.676200000
6	24.595900000	5.442200000	3.460500000
6	23.658800000	6.408000000	3.879200000
1	23.676600000	7.473100000	3.721800000
6	22.711400000	5.709700000	4.600500000
6	21.502400000	6.133700000	5.287000000
6	20.994400000	5.443400000	6.390900000
1	21.526200000	4.584800000	6.787400000
6	19.839800000	5.838600000	7.035700000
1	19.477300000	5.279400000	7.892200000
6	19.139200000	6.974100000	6.593600000
6	26.660800000	4.553100000	2.405100000
1	26.400800000	3.573700000	2.782900000
6	27.819000000	4.718700000	1.667900000
1	28.464000000	3.874100000	1.467800000
6	19.652200000	7.676200000	5.489700000
1	19.117000000	8.548700000	5.137100000
6	20.802600000	7.261200000	4.854600000
1	21.157200000	7.809000000	3.989300000
6	15.167600000	5.551900000	10.925400000
6	14.276200000	5.758600000	9.697300000
8	16.289300000	4.754900000	10.620400000
1	14.606400000	4.930100000	11.630400000
6	15.521800000	6.853900000	11.631000000
1	16.170300000	6.646500000	12.482900000
1	16.038300000	7.541000000	10.964800000
1	14.623000000	7.342200000	12.008800000
35	12.428400000	6.219600000	10.237900000
1	14.620000000	6.563300000	9.056600000
1	14.197700000	4.837600000	9.121000000
6	17.234200000	5.169100000	9.689900000
8	18.204800000	4.439600000	9.604200000
1	17.693300000	6.852900000	7.936400000
8	16.951600000	6.221500000	9.060700000

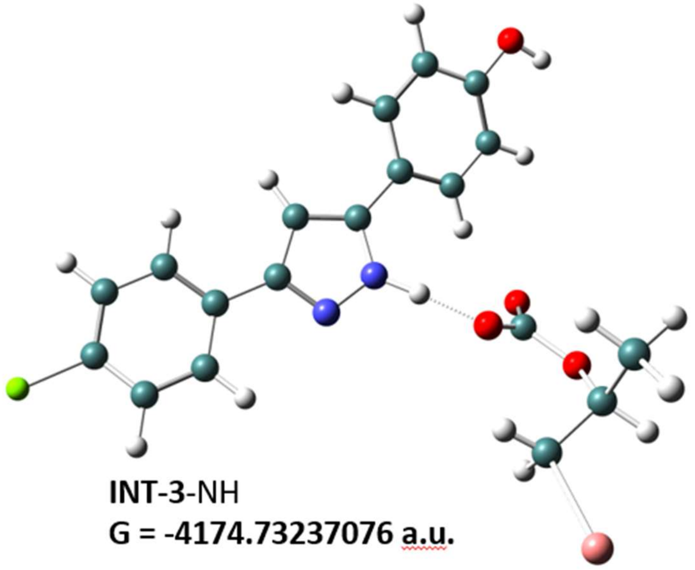

17	29.850400000	7.344400000	1.513400000
8	17.110000000	5.108800000	5.405700000
1	16.889400000	4.823000000	6.295500000
7	24.791100000	4.465900000	4.946800000
7	23.540400000	4.273500000	5.312000000
6	28.373900000	6.782100000	2.234900000
6	27.163600000	7.308400000	1.819400000
1	27.134100000	8.065000000	1.047200000
6	25.990600000	6.857100000	2.402500000
1	25.050900000	7.276500000	2.067400000
6	25.999400000	5.883500000	3.400500000
6	24.763200000	5.412400000	4.017800000
6	23.441500000	5.830200000	3.784500000
1	23.083900000	6.599500000	3.120200000
6	22.676600000	5.066700000	4.645400000
6	21.233700000	5.063200000	4.869200000
6	20.687000000	4.722500000	6.105100000
1	21.315200000	4.453000000	6.947000000
6	19.315700000	4.730900000	6.295000000
1	18.929200000	4.453800000	7.270700000
6	18.458800000	5.087600000	5.261500000
6	27.236900000	5.370400000	3.797300000
1	27.265000000	4.615300000	4.570700000
6	28.415500000	5.811100000	3.223400000
1	29.363800000	5.402200000	3.545000000
6	18.990800000	5.437300000	4.025300000
1	18.327900000	5.708000000	3.213600000
6	20.361400000	5.416600000	3.839600000
1	20.762100000	5.663900000	2.864200000
6	23.139000000	0.002800000	8.398900000
6	24.602700000	0.144800000	7.972000000
8	22.636500000	1.207500000	8.926600000
1	23.119500000	-0.673200000	9.259700000
1	24.718600000	0.561200000	6.977200000
1	25.155400000	0.750000000	8.689300000
6	22.254300000	-0.594800000	7.313400000
1	21.228900000	-0.674100000	7.676700000
1	22.254700000	0.021900000	6.417300000
1	22.591500000	-1.597800000	7.049700000
35	25.501300000	-1.621200000	7.949000000
1	23.319300000	3.516900000	6.018700000
6	22.554400000	2.353500000	8.141800000
8	21.983300000	3.284300000	8.687300000
8	23.076500000	2.281400000	7.003200000

Figure S53. Optimized structures of the intermediate INT3 considering a supermolecule approach with the carbon dioxide addition and its Gibbs energy values. Coordinates are also shown.

TS3 – Freq imag. = -450.97i cm^{-1}
G = -4174.71789758 a.u.

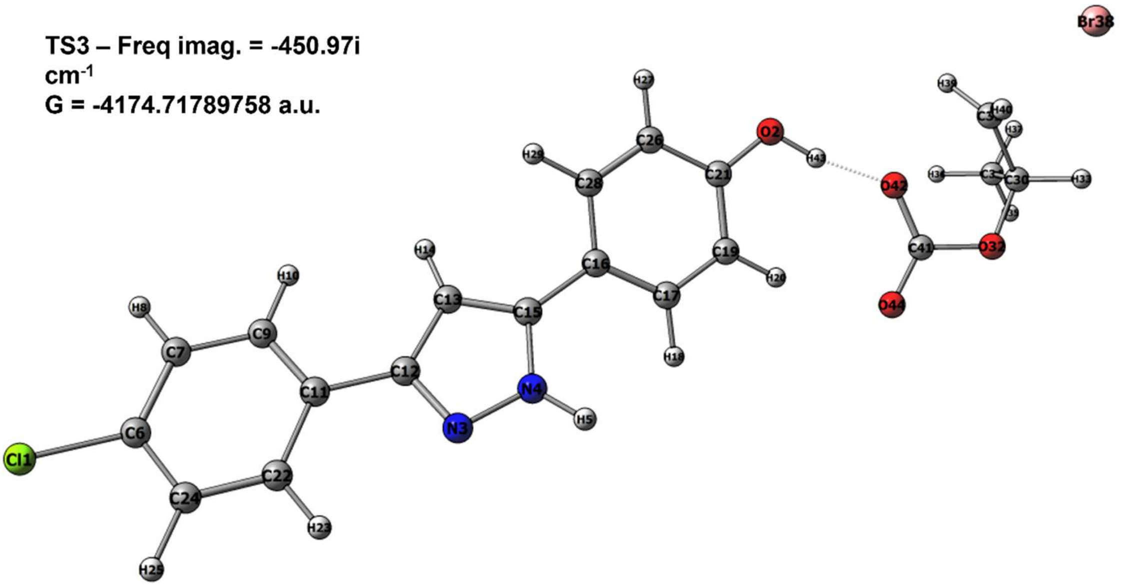

17	29.842700000	6.907800000	1.356200000
8	17.334300000	7.212400000	6.518200000
7	23.867700000	4.547900000	3.410200000
7	22.647500000	4.653100000	3.921600000
1	22.071300000	3.833600000	3.996400000
6	28.240600000	6.585100000	1.938300000
6	27.415800000	7.638100000	2.291500000
1	27.764800000	8.657800000	2.203100000
6	26.138600000	7.373300000	2.758000000
1	25.500500000	8.204500000	3.027400000
6	25.666300000	6.068100000	2.879900000
6	24.319900000	5.790000000	3.375000000
6	23.361400000	6.693800000	3.874400000
1	23.449500000	7.758000000	4.012600000
6	22.273300000	5.917300000	4.227900000
6	20.987900000	6.240900000	4.809700000
6	20.298300000	5.335800000	5.625800000
1	20.743700000	4.373100000	5.860900000
6	19.081600000	5.639400000	6.185600000
1	18.591400000	4.908200000	6.815000000
6	18.446100000	6.903600000	5.983100000
6	26.520800000	5.026500000	2.515000000
1	26.171100000	4.007100000	2.605200000
6	27.797800000	5.275900000	2.047000000
1	28.447700000	4.456900000	1.770000000
6	19.162100000	7.803500000	5.137900000
1	18.715500000	8.770100000	4.937800000
6	20.378800000	7.480600000	4.583500000
1	20.872300000	8.199200000	3.937200000
6	16.610500000	6.098400000	9.369800000
6	15.684100000	7.294600000	9.582900000
8	16.495200000	5.752400000	8.040300000
1	16.229400000	5.270200000	9.985300000
6	18.055000000	6.354600000	9.768700000
1	18.652400000	5.474900000	9.527600000
1	18.468800000	7.194800000	9.208500000
1	18.168500000	6.559400000	10.833600000
35	15.578700000	7.886200000	11.461300000
1	16.021700000	8.153100000	9.000300000
1	14.665700000	7.040300000	9.291600000
6	14.794400000	4.234900000	7.422100000
8	14.331900000	4.195400000	8.494700000
1	16.848800000	6.345500000	7.454000000
8	15.168600000	4.186600000	6.327800000

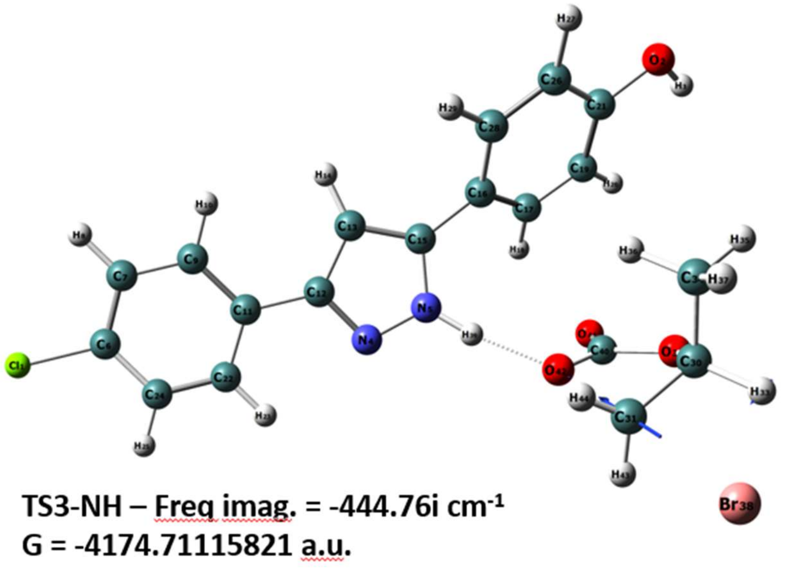

17	29.354200000	7.721000000	0.885000000
8	17.609700000	3.832400000	6.437300000
1	17.519700000	3.785000000	7.392300000
7	25.142600000	4.685700000	5.210800000
7	23.967700000	4.360600000	5.711200000
6	28.058600000	7.053400000	1.826800000
6	26.747600000	7.295700000	1.456500000
1	26.529300000	7.897600000	0.585000000
6	25.718600000	6.759300000	2.212900000
1	24.697900000	6.955900000	1.911900000
6	25.973200000	5.981700000	3.341100000
6	24.885000000	5.423100000	4.140100000
6	23.495600000	5.559400000	3.955600000
1	22.964600000	6.115800000	3.201400000
6	22.934200000	4.855900000	4.999700000
6	21.543100000	4.629800000	5.380300000
6	21.164000000	4.643400000	6.719300000
1	21.888000000	4.857600000	7.493300000
6	19.860000000	4.373600000	7.092600000
1	19.615800000	4.357700000	8.149400000
6	18.897700000	4.107100000	6.126200000
6	27.305800000	5.753400000	3.689800000
1	27.522900000	5.151100000	4.561500000
6	28.343100000	6.282400000	2.943400000
1	29.369600000	6.095600000	3.228300000
6	19.256000000	4.114300000	4.781800000
1	18.509600000	3.894600000	4.029600000
6	20.567100000	4.366300000	4.420600000
1	20.845500000	4.328200000	3.374900000
6	22.009800000	0.445400000	7.714400000
6	23.401200000	0.472400000	7.108700000
8	21.936700000	1.361100000	8.798100000
1	21.832900000	-0.533500000	8.154800000
6	20.943800000	0.747800000	6.676300000
1	19.954000000	0.748400000	7.132800000
1	21.104400000	1.723600000	6.215800000
1	20.962300000	-0.012300000	5.896300000
35	23.449600000	-1.853000000	6.296600000
1	23.890700000	3.714700000	6.513700000
6	22.673400000	2.489700000	8.613300000
8	22.507000000	3.424100000	9.365600000
8	23.482300000	2.398100000	7.625200000
1	24.266900000	0.264400000	7.716700000
1	23.568500000	0.773000000	6.089100000

Figure S54. Optimized structures of the transition state TS3 with the releasing of the bromine and ring closure. Coordinates are also shown.

Final Product
G = -4174.74748276 a.u.

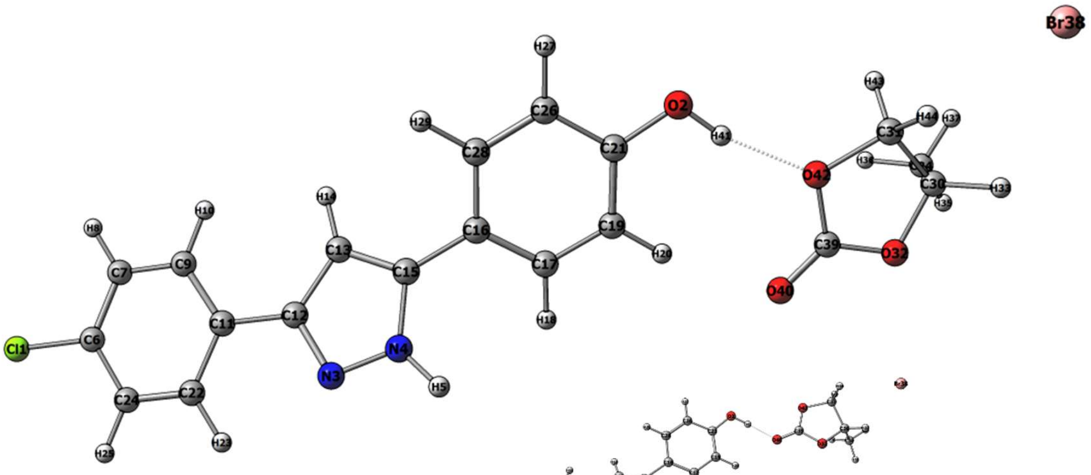

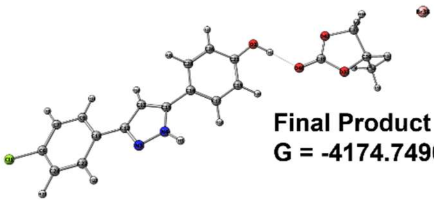

Final Product – lowest energy
G = -4174.74962244 a.u.

17	28.926500000	6.497900000	0.136000000
8	18.036600000	6.870100000	8.119900000
7	24.112500000	4.126600000	4.230700000
7	23.046000000	4.226900000	5.009300000
1	22.649600000	3.392000000	5.404400000
6	27.598400000	6.169500000	1.198000000
6	26.675600000	7.164500000	1.470400000
1	26.779600000	8.142600000	1.021400000
6	25.615100000	6.893300000	2.319200000
1	24.895600000	7.677200000	2.516400000
6	25.458700000	5.639900000	2.906100000
6	24.334000000	5.356000000	3.797400000
6	23.375600000	6.250500000	4.316600000
1	23.314500000	7.317100000	4.181700000
6	22.547200000	5.479800000	5.104200000
6	21.381200000	5.817200000	5.908400000
6	21.017900000	5.073500000	7.030400000
1	21.626400000	4.236100000	7.353000000
6	19.904300000	5.392700000	7.785200000
1	19.650400000	4.793200000	8.649800000
6	19.113100000	6.494900000	7.442600000
6	26.404100000	4.656000000	2.614600000
1	26.301600000	3.676800000	3.062200000
6	27.467600000	4.912800000	1.768600000
1	28.193400000	4.140300000	1.554300000
6	19.477900000	7.252100000	6.323300000
1	18.868400000	8.102100000	6.045500000
6	20.584400000	6.913500000	5.574000000
1	20.827400000	7.501100000	4.697200000
6	15.036000000	4.105800000	12.246700000
6	14.400000000	5.289300000	11.522800000
8	16.243600000	3.897000000	11.462300000
1	14.415400000	3.217100000	12.166800000
6	15.398500000	4.375800000	13.686300000
1	15.975200000	3.552500000	14.108100000
1	15.978200000	5.295600000	13.793700000
1	14.466400000	4.471600000	14.242500000
35	11.781800000	4.122700000	13.104900000
6	16.488100000	4.961900000	10.732700000
8	17.496300000	5.106500000	10.081500000
1	17.828100000	6.259200000	8.861800000
8	15.517300000	5.847800000	10.790400000
1	13.993700000	6.040600000	12.192600000
1	13.620400000	4.990500000	10.824800000

Final Product-NH
G = -4174.74335320 a.u.

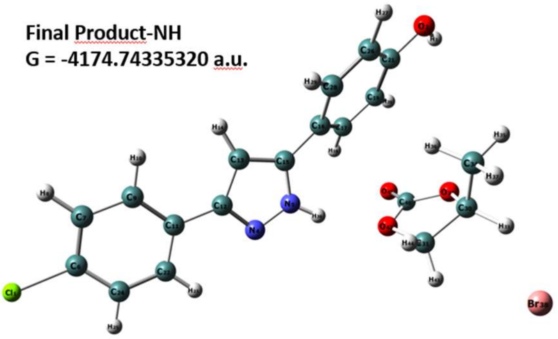

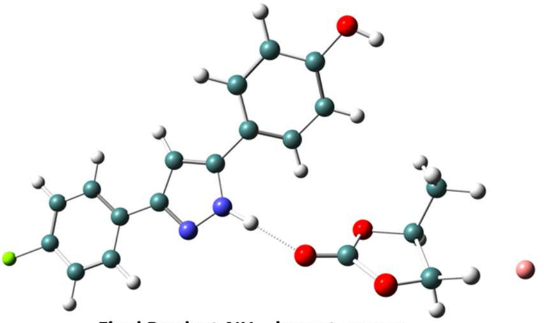

Final Product-NH – lowest energy
G = -4174.74970684 a.u.

17	27.458800000	10.874800000	0.793900000
8	20.534600000	0.800900000	6.594300000
1	20.913400000	0.229500000	7.267200000
7	26.423800000	4.939800000	3.822900000
7	25.714400000	3.985800000	4.392900000
6	26.901500000	9.429800000	1.573800000
6	25.552600000	9.266400000	1.836700000
1	24.845300000	10.035500000	1.558400000
6	25.118000000	8.108000000	2.460400000
1	24.060500000	7.992500000	2.659900000
6	26.010200000	7.104200000	2.830200000
6	25.555100000	5.880200000	3.486700000
6	24.250100000	5.501100000	3.854600000
1	23.333300000	6.056400000	3.746700000
6	24.393500000	4.261400000	4.442100000
6	23.405800000	3.360800000	5.026400000
6	23.734300000	2.490600000	6.062600000
1	24.732000000	2.481300000	6.478300000
6	22.792700000	1.627800000	6.595100000
1	23.089900000	0.956800000	7.393800000
6	21.490100000	1.624600000	6.111100000
6	27.364900000	7.296700000	2.552900000
1	28.072000000	6.527800000	2.832400000
6	27.812200000	8.447200000	1.930300000
1	28.865100000	8.579700000	1.722300000
6	21.141700000	2.499600000	5.086400000
1	20.130200000	2.495700000	4.702000000
6	22.093600000	3.347300000	4.552800000
1	21.816300000	3.999400000	3.734400000
6	26.655200000	-0.531100000	7.808200000
6	27.677100000	-1.277200000	6.951800000
8	26.558000000	0.739200000	7.113800000
1	27.041200000	-0.351500000	8.808700000
6	25.298600000	-1.189000000	7.878100000
1	24.589800000	-0.566100000	8.426100000
1	24.891700000	-1.391900000	6.884600000
1	25.420600000	-2.127200000	8.419500000
35	27.814700000	-3.057700000	9.721700000
1	26.180000000	3.124700000	4.683600000
6	27.084700000	0.638100000	5.903600000
8	27.028100000	1.501800000	5.069200000
8	27.681100000	-0.522600000	5.720900000
1	28.673800000	-1.274200000	7.388600000
1	27.396100000	-2.305800000	6.745200000

Figure S55. Optimized structures of the final structure (products) also considering a supermolecule approach with the two isomers found. Coordinates for the most stable geometries are shown.

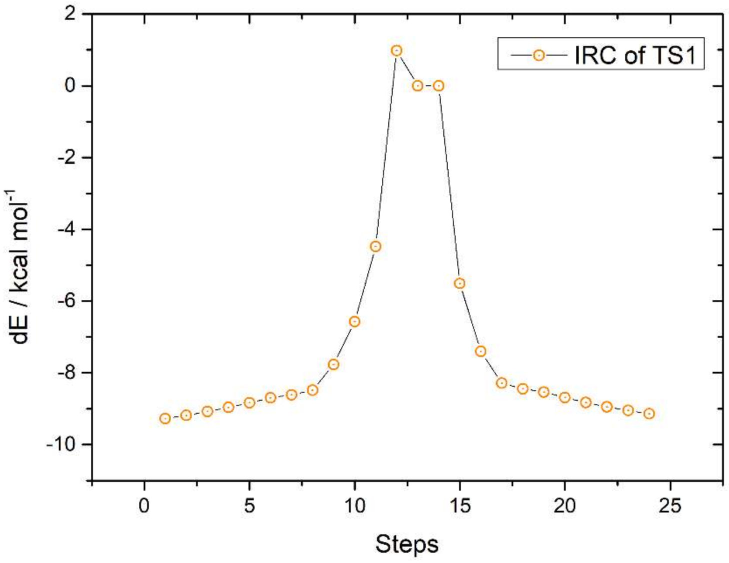

Figure S56. IRC (intrinsic reaction coordinate) plot for the first transition state (TS1-OH).

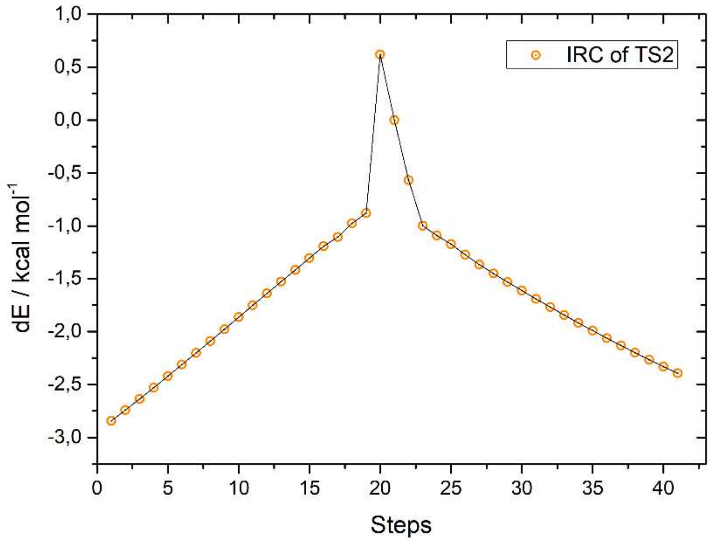

Figure S57. IRC (intrinsic reaction coordinate) plot for the second transition state (TS2-OH). It was found an imaginary frequency of 251.79i cm^{-1} (TS2-OH) and 235.44i cm^{-1} (TS2-NH).

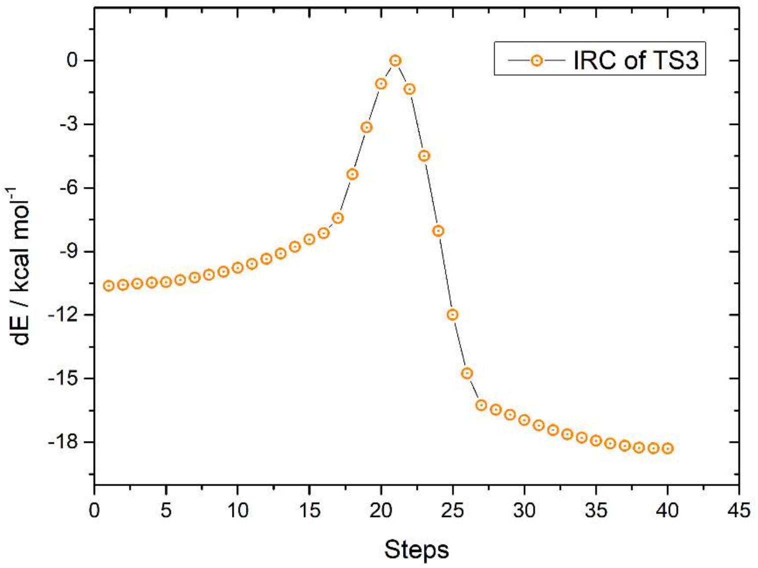

Figure S58. IRC (intrinsic reaction coordinate) plot for the third transition state (TS3-OH). TS3-OH with imaginary frequency of 450.97i cm-1; TS3-NH with imaginary frequency of 444.76i cm^{-1}.

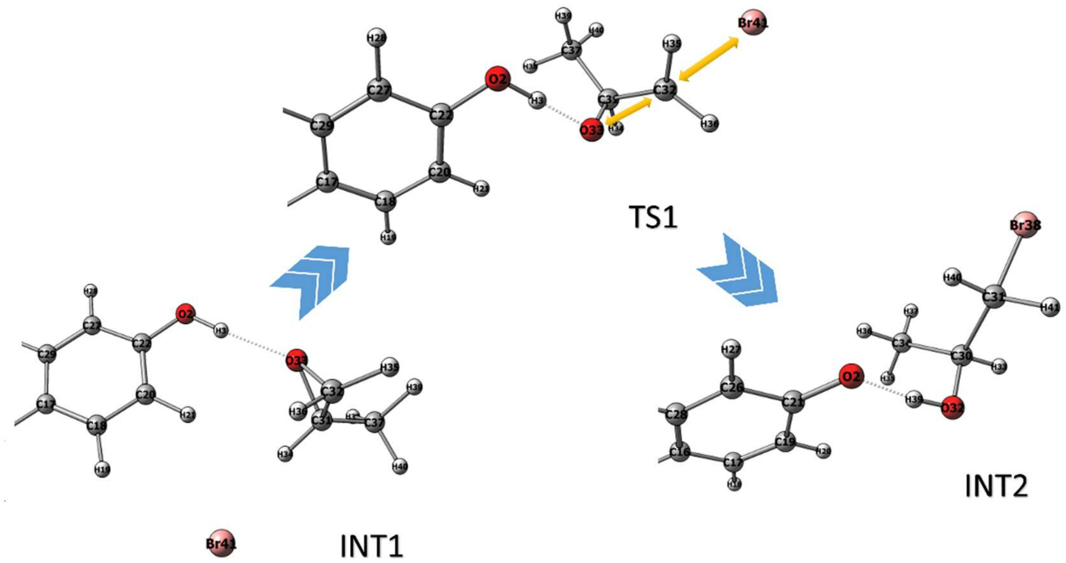

Figure S59. Optimized structures of the species directly involved in the first energy barrier. The yellow doubled arrows in TS1 represent the characteristic vibrational mode illustrating the epoxide opening. TS1 has been characterized by one imaginary frequency of 475.38i cm^{-1} (TS1-OH) and 469.25i cm^{-1} (TS1-NH).

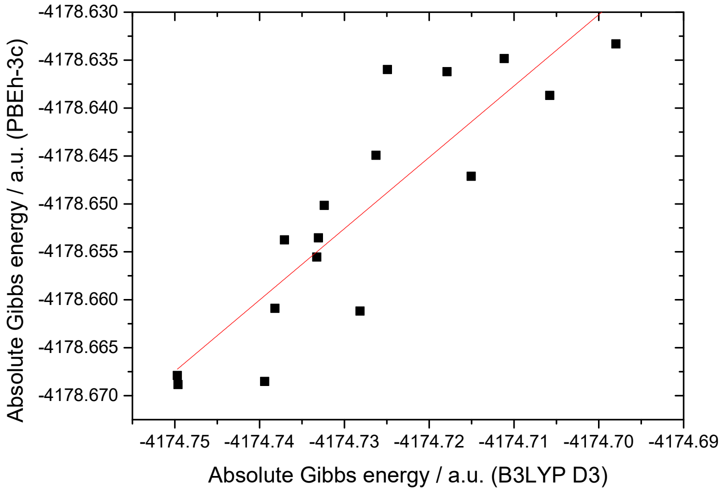

Figure S60. Correlation analysis between Gibbs free energy for PBEh-3c *vs*. B3LYP D3. The corresponding linear equation is y = -1072.01 + 0.744x.

Table S3 provides the main bond lengths for the atoms involved in the catalytic reaction. Following each column of data, it is possible to understand the whole history of what is involved in the catalytic reaction. For example, the bond length O2-H6 (OH site) and N5-H6 (NH site) show the proton transfer to O33 (epoxide, second column) and return to its original atom, which has already been seen in Figure 8b. Another important feature to highlight is the approach and subsequent addition of the CO2 molecule. In this case, the data related to C42 and O44 (two last columns) must be emphasized. That proton H6 now interacts with the oxygen atom originally from carbon dioxide which is now forming the final carbonate product. Basically, all the data presented in Table S3 are quite similar for both pathways, NH and OH.

Table S3. Electronic energies for all species.

Species	MP2 Total Energy	Electronic Energy	MP2 Total Energy+CO2	Electronic Energy+CO2	Percentage difference
INT-1-NH	-3986,726203	-3986,815176	-4175,034929	-4175,006378	2,855
INT-1-OH	-3986,716223	-3986,802120	-4175,024948	-4174,993323	3,163
TS1-OH	-3986,691353	-3986,777839	-4175,000078	-4174,969042	3,104
TS1-NH	-3986,686918	-3986,769986	-4174,995643	-4174,961188	3,445
INT-2-OH	-3986,719423	-3986,813050	-4175,028148	-4175,004252	2,390
INT-2-NH	-3986,721720	-3986,807658	-4175,030446	-4174,998860	3,159
INT-2-NH-CO2	-4175,037417	-4175,009195	-4175,037417	-4175,009195	2,822
INT-2-OH-CO2	-4175,037058	-4175,015031	-4175,037058	-4175,015031	2,203
TS2-OH	-4175,012964	-4175,006295	-4175,012964	-4175,006295	0,667
TS2-NH	-4175,023975	-4174,996925	-4175,023975	-4174,996925	2,705
INT-3-NH	-4175,038295	-4175,019382	-4175,038295	-4175,019382	1,891
INT-3-OH	-4175,038563	-4175,025734	-4175,038563	-4175,025734	1,283
TS3-NH	-4175,014946	-4174,999326	-4175,014946	-4174,999326	1,562
TS3-OH	-4175,012900	-4175,005204	-4175,012900	-4175,005204	0,770
Product NH-final	-4175,054793	-4175,038326	-4175,054793	-4175,038326	1,647
Product OH-final	-4175,054124	-4175,040414	-4175,054124	-4175,040414	1,371
